# Occurrence of Harmful Cyanobacteria in Drinking Water from a Severely Drought-Impacted Semi-arid Region

**DOI:** 10.3389/fmicb.2018.00176

**Published:** 2018-02-28

**Authors:** Juline M. Walter, Fabyano A. C. Lopes, Mônica Lopes-Ferreira, Lívia M. Vidal, Luciana Leomil, Fabiana Melo, Girlene S. de Azevedo, Rossandra M. S. Oliveira, Alba J. Medeiros, Adriana S. O. Melo, Carlos E. De Rezende, Amilcar Tanuri, Fabiano L. Thompson

**Affiliations:** ^1^Laboratory of Microbiology, Institute of Biology, and SAGE-COPPE, Federal University of Rio de Janeiro, Rio de Janeiro, Brazil; ^2^Immunoregulation Unit, Special Laboratory of Applied Toxinology, Butantan Institute, São Paulo, Brazil; ^3^Instituto de Pesquisa Professor Joaquim Amorim Neto, Campina Grande, Brazil; ^4^Secretaria de Saúde de Campina Grande, Campina Grande, Brazil; ^5^Laboratory of Environmental Sciences, State University of Northern Rio de Janeiro, Campos dos Goytacazes, Brazil; ^6^Laboratory of Virology, Institute of Biology, Federal University of Rio de Janeiro, Rio de Janeiro, Brazil

**Keywords:** cyanotoxins, zebrafish, metagenomics, public healthy, eutrophication

## Abstract

Harmful cyanobacterial blooms have become increasingly common in freshwater ecosystems in recent decades, mainly due to eutrophication and climate change. Water becomes unreliable for human consumption. Here, we report a comprehensive study carried out to investigate the water quality of several Campina Grande reservoirs. Our approach included metagenomics, microbial abundance quantification, ELISA test for three cyanotoxins (microcystin, nodularins, and cylindrospermopsin), and *in vivo* ecotoxicological tests with zebrafish embryos. Cytometry analysis showed high cyanobacterial abundance, while metagenomics identified an average of 10.6% of cyanobacterial sequences, and demonstrated the presence of *Microcystis*, *Cylindrospermopsis*, and toxin coding genes in all ponds. Zebrafish embryos reared with pond water had high mortality and diverse malformations. Among the ponds analyzed, Araçagi showed the highest lethality (an average of 62.9 ± 0.8%), followed by Boqueirão (lethality average of 62.5 ± 0.8%). Here, we demonstrate that water from ponds undergoing extremely drought conditions have an abundance of potentially harmful cyanobacteria and their toxins. Our findings are consistent with a scenario in which polluted drinking water poses a great risk to human health.

## Introduction

Worldwide, approximately 884 million people lack access to clean drinking water, the majority of which reside in semi-arid poor geographic areas of sub-Saharan Africa and Asia (e.g., Afghanistan and Pakistan)^1^. In Latin America, some regions, such as in Northeastern Brazil, still rely on natural or man-made shallow standing water ponds. Extremely harsh climatic conditions and drought have resulted in dry-out of these water bodies. The current drought period began 6 years ago and is considered the worst of at least 10 large droughts in the last century (Martins et al., 2015). The Boqueirão pond, which has a capacity of approximately 411 million m^3^, experienced approximately 97% volume decrease in March 2017 (Brazil, 2017). Boqueirão experiences high temperatures and high turbidity throughout the year, which is associated with a constant state of eutrophication through nutrient inputs. This pond supplies water to 1 million people throughout 19 cities. Despite the need to remove harmful cyanobacteria and toxins and perform clarification (coagulation and flocculation), disinfection, and pH correction as preconized by Resolution 357/05 from the Brazilian National Environment Council (CONAMA) (Brazil National Environment Council/Conselho Nacional de Meio Ambiente - CONAMA, 2005), pond water is transported by means of water trucks to nearby cities households as drinking water without any treatment. The extremely poor water governance is the consequence of a complex context.

Water governance is a conceptual framework that comprises different aspects, including (i) how (federal, state, and municipality) institutions operate, (ii) how (federal, state, and municipality) regulations affect political actions and societal concerns through formal and informal instruments, and (iii) how the above mentioned (i) and (ii) enable practical management tools to be applied (Tortajada, 2010; UNDP, 2010). A recent study modeled political, economic, social, and environmental variables that impact water sector performance in Brazil (Kayser et al., 2015). It became clear from this study that simple actions such as coordination and data sharing between ministries that deal with drinking water services, monitoring and enforcement of water quality laws, and sufficient technical capacity to improve administrative and technical management of water services at the local level could improve dramatically water governance in Brazil. Relevant technical aspects to improve water governance include the monitoring of water quality by means of (in)organic chemistry analysis, toxicological tests, and metagenomics.

The combination of high nutrient loads and high temperature promotes the formation of toxic cyanobacterial blooms in freshwater bodies (Paerl and Huisman, 2008). The occurrence of cyanobacterial blooms in aquatic ecosystems has increased in extension and frequency and is becoming a potential threat to both human and ecosystem health worldwide (Sangolkar et al., 2009; Paerl and Otten, 2013). Even developed countries may face severe water shortages (Qin et al., 2010; Tanber, 2014). In 2007, a massive *Microcystis* bloom in the Lake Taihu, China, affected approximately 10 million people, of which more than 2 million have the water supply cut off for at least a week (Qin et al., 2010). High nitrogen and phosphorus levels control the development of blooms (Graham et al., 2004; Rinta-Kanto et al., 2009; Orihel et al., 2012; Berry et al., 2017), and the balance between these nutrients promotes a shift from non-toxic to toxic cyanobacterial species (Gobler et al., 2016). The most frequently detected and widespread cyanotoxins in freshwater are hepatotoxins (i.e., microcystins, nodularins, and cylindrospermopsins), which could bioaccumulate (Christen et al., 2013; Li et al., 2013; Cai et al., 2015a,b; Zhao et al., 2015). Severe human poisoning by cylindrospermopsins was first recorded in Australia (Byth, 1980) and England (Turner et al., 1990). In Brazil, the first documented combined microcystin and cylindrospermopsin poisoning episode occurred in Caruaru city in 1996, after a stronger drought, causing 76 deaths at a hemodialysis clinic (Pouria et al., 1998; Carmichael et al., 2001; Azevedo et al., 2002). Previous studies have shown that *Microcystis* may be found in ponds of Northeastern Brazil (Bouvy et al., 2000; Huszar et al., 2000; Vasconcelos et al., 2011). Microcystin is known to be produced by *Microcystis*, *Anabaena*, *Planktothrix*, and *Nostoc* toxic species (Sivonen and Jones, 1999; Hotto et al., 2007; Dittmann et al., 2013), which harbor microcystin synthetase (*mcy*ABCD) gene clusters, directly involved in the microcystin production, and bi-directionally transcribed central promoters (*mcy*A/D) (Kaebernick et al., 2002). Microcystin and other cyanotoxins are known to produce malformations and killing zebrafish (*Danio rerio*) (Dao et al., 2013; Pavagadhi et al., 2013) and medaka embryos (*Oryzias latipes*) (Adámek et al., 2011) in controlled laboratory conditions.

The aim of the present study was to analyze the water quality and the toxicity potential of three major ponds Araçagi, Boqueirão, and Saulo Maia, and two minor ponds Galante and Mazagão, located in the semi-arid region of Campina Grande (Paraíba, Brazil). We hypothesized that potentially harmful cyanobacteria and other microbes might be a significant component of the water making it improper for consumption as drinking water without treatment.

## Materials and Methods

### Study Area and Sample Collection

**FIGURE 1:**
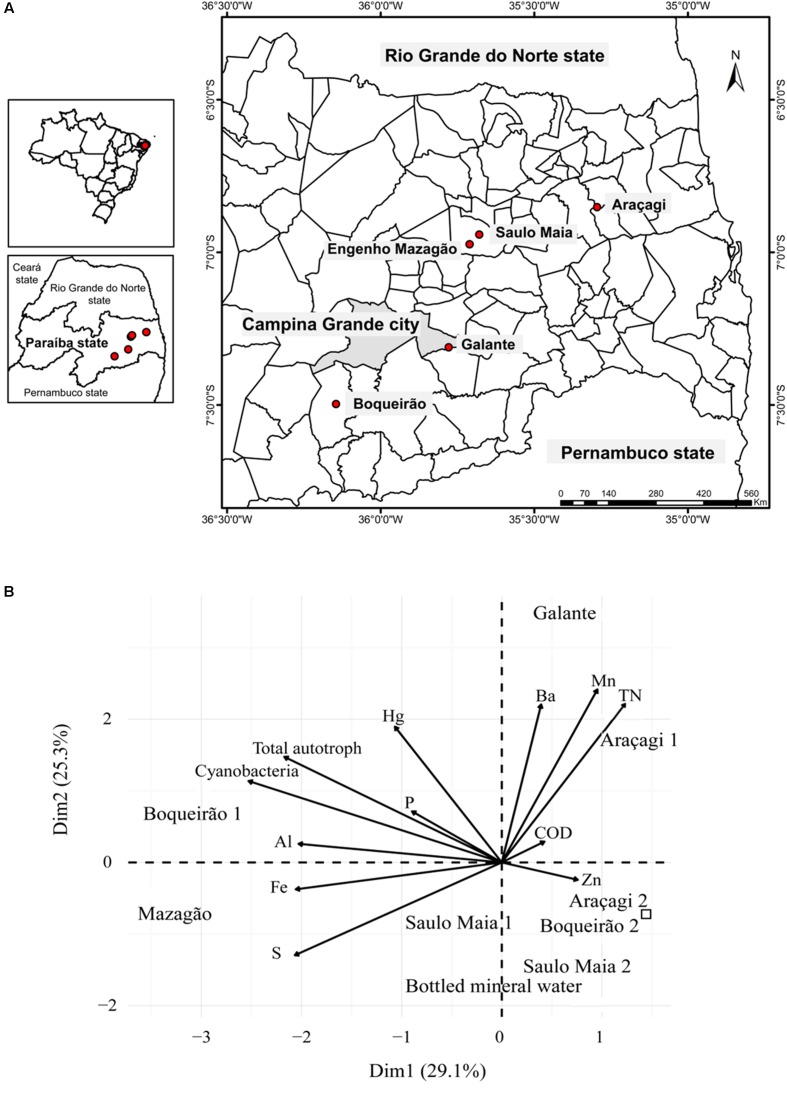
General overview of the sampling. **(A)** Position of the sampling locations. The study was carried out in ponds located in Paraíba State (Brazil), which have been affected by a persistent dry period. Filled circles denote pond sites. Water was sampled approximately 0.5 m below the water surface twice during September and October 2016. **(B)** Principal component analysis of the ponds based on chemical and biological variables showing differential features of each pond. Variables (arrows) and sampling sites (Araçagi, Boqueirǎo, Saulo Maia, Galante, and Mazagǎo). Control is represented by the square. The sampling ponds occupied different ordination space composed by PC1 and PC2.

Water samples were collected from ponds located in Campina Grande metropolitan region (Paraíba, Brazil). In total, five ponds were sampled (Supplementary Table S1). Three ponds are routinely used to human consumption: Araçagi (6°51′9.396″S/35°17′43.1982″W), Boqueirão (7°29′50.8344″S/36°8′41.3628″W), and Saulo Maia (6°56′31.2756″S/35°40′43.4418″W) (**Figure 1**). These ponds were, therefore, sampled twice between 21 and 24 September and between 29 and 31 October 2016. Galante and Mazagão are both in hard-to-reach locations and have low quantities of water (**Figure 1A**). Boqueirão is also named Epitácio Pessoa pond. Water was sampled approximately 0.5 m below the water surface, and in a 3–5-m distance from the border, totaling approximately 20 l of unfiltered water collected in each pond. In the field, water aliquots were stored immediately in the dark on wet ice for chemical analyses, total/photoautotrophic microbial counts, metagenomics, and zebrafish analyses. Three water replicates (250 ml each) were collected in sterile polyethylene bottles with the corresponding water for chemical and zebrafish analyses. For microbial counts, three 1.5-ml aliquots were dispensed into 2.0-ml cryogenic tubes, fixed with 10% paraformaldehyde and 0.5% glutaraldehyde for approximately 10 min at room temperature (22 ± 2°C), and stored in liquid nitrogen for the subsequent microbial counts. For metagenomics analyses, 2-l samples were prefiltered using a 20-μm mesh and then filtered through 0.22-μm Sterivex^TM^ Filter Units (Millipore^®^, Darmstadt, Germany), by a positive pressure using a peristaltic pump. The Sterivex^TM^ filters were stored in liquid nitrogen for further DNA extraction at Federal University of Rio de Janeiro (UFRJ).

Sampling was performed under the Brazilian Environmental Agency, Instituto Chico Mendes de Conservação da Biodiversidade (ICMBio), under SISBIO License No. 21811–1.

### Physical and Chemical Analyses

Measurements of total nitrogen were determined using a potassium persulfate digestion methodology (Grasshoff et al., 1999). Dissolved oxygen concentration (DOC) was analyzed as described previously (Rezende et al., 2010). Quantification of trace elements was carried out by inductively coupled plasma optical emission spectrometry (ICP-OES; Varian Liberty-Series II) using a procedure based on the Method 3052 (US Environmental Protection Agency) modified by Marques et al. (2011). Water samples were used to determine concentrations of the following elements: Al, As, Ba, Cd, Co, Cr, Cu, Fe, Hg, Mn, Ni, P, Pb, S, Se, Ti, and Zn. Measurements were carried out in triplicate for each sample and a coefficient of variation between replicates <10% was considered satisfactory. DOC, pH, and temperature measurements were performed *in situ* using a multiparameter sensor model D-22 (Digimed, São Paulo, Brazil) (Supplementary Table S1).

### Microbial Abundance

Microbial abundance in water samples was determined by flow cytometry, with an Accuri C6 flow cytometer (Becton Dickinson Biosciences, Franklin Lakes, NJ, United States) equipped with a blue laser beam set to 488 nm and with the original filter set-up. The total microbial cell counts were determined on samples stained with SYBR^®^ Green I (Invitrogen, Carlsbad, CA, United States) and the photoautotrophic counts were determined in unstained samples. Bead solutions were used to adjust and calibrate the flow rate increase, and used as an indicator of correct fluorescence analysis. One-way analysis of variance (ANOVA) with a Bonferroni–Holm *post hoc* test correction for multiple comparisons was performed to find differences in microbial abundance between time points and ponds. For this test, an alpha ≤ 0.05 indicated statistical significance. Cytometry was carried out in the Laboratory of Microbiology (UFRJ, Rio de Janeiro, Brazil).

### *In Vivo* Experiments with Zebrafish Embryos

Wild-type embryo zebrafish (*D. rerio*) was maintained in standard laboratory conditions at Butantan Institute (São Paulo, Brazil). Zebrafish rearing conditions were as follow: temperature (28 ± 1°C), pH (7.0 ± 0.1), and photoperiod (14:10 h light:dark). Water purified by reverse osmosis was supplemented with 0.6% Instant Ocean salt. Each treatment had 20 embryos reared in 2 ml of E2 medium supplemented with 50 μl of pond water. Experiments were repeated in three independent time periods. Each independent experiment included three negative controls (standard medium). Unaffected, malformed (i.e., teratogenicity), and dead zebrafish embryos were reported daily. Teratogenicity encompasses malformations (i.e., any deviation of normal development) that include heart edema, spine, yolk and mouth deformation, and absence of pigmentation. Malformations were analyzed under a stereomicroscope and the observations of morphological endpoints were conducted as described by Nagel (2002). Standard length of embryos was defined by OECD guidelines 236 (OECD, 2013). Statistical analyzes were performed with Student’s paired *t*-test. *P*-values of ≤5% were considered statistically significant.

All the procedures involving animals were carried out in accordance with the guidelines provided by the Animal Ethics Committee of the Butantan Institute, Brazil.

### DNA Extraction and Shotgun Metagenomic Sequencing

Total DNA was extracted and purified by the NucleoSpin^®^ Tissue Kit (Macherey-Nagel, Düren, Germany), using a modified protocol to complete the lysis in the Sterivex^TM^ filters. Briefly, we used proteinase K (20 mg ml^-1^) together with SDS (20%), instead of the manufacture’s buffer T1. Metagenomic DNA libraries were prepared with the Nextera XT DNA Library Preparation Kit (Illumina, San Diego, CA, United States) and 2 × 300-bp paired-end sequencing was performed on a MiSeq machine (Illumina, San Diego, CA, United States), according to the manufacturer’s instructions. The metagenomic sequencing was carried out in the Laboratory of Microbiology (UFRJ, Rio de Janeiro, Brazil). A total of 8.34 million reads (raw sequences) were generated by Illumina MiSeq sequencing from all pond samples (Supplementary Table S3).

### Pre-processing and Metagenomic Analysis

The paired-end merging was performed using PEAR v.0.9.6 (Zhang et al., 2014) with default parameters (minimum overlap size, 10; minimum possible length of the assembled sequences, 50; *p*-value, 0.01). Quality analysis was performed using Prinseq-lite v.0.20.4 (Schmieder and Edwards, 2011) with the following parameters: minimum sequence length, 75; minimum mean quality score, 30; maximum percentage of Ns, 1; trim 20 nucleotides from left; and trim 20 nucleotides from right. Metagenomes were aligned against the NCBI non-redundant protein sequences (nr) database^2^ (October 2016) using DIAMOND (version 0.7.1) (Buchfink et al., 2015) with default parameters. DNA sequences were assigned to a taxon ID based on the NCBI taxonomy. Functional annotation was obtained with SEED (Overbeek et al., 2005) and COG database (Tatusov et al., 2000).

Principal component analysis (PCA) of physicochemical parameters and microbial abundance was performed using a correlation matrix with FactoMineR (Lê et al., 2008) and factoextra (Kassambara, 2015) packages in R statistical software (R Development Core Team, 2016). Factoextra package was used to visualize the results from PCA through ggplot2 (Wickham, 2009). We used PCA to characterize the sampling sites and to identify the environmental parameters that contributed to the differences among sites. Non-ribosomal peptide synthetase genes (COG 1020) were obtained using the NCBI’s reference sequence (RefSeq) database^3^ (Pruitt et al., 2007) and BLASTX (Altschul et al., 1990).

### Cyanotoxin Determination by ELISA Technique

The analyses of the water samples for detection of microcystins, nodularin, and cylindrospermopsin were performed using the commercially available enzyme-linked immunosorbent assay (ELISA) kits: Microcystin ELISA Plate Kit and Cylindrospermopsin ELISA Plate Kit (Abraxis Inc., Warminster, PA, United States), according to the manufacturer’s protocol (Abraxis, 2016a,b). ELISA is a quantitative and competitive immunosorbent assay that allows the congener-independent presence of each toxin in water samples. Frozen water samples were thawed, re-frozen, and thawed again prior to ELISA analysis, using aliquots of 100 μl. Absorbances were read using a microplate ELISA spectrophotometer (BioTek Instruments, Inc., Winooski, VT, United States), and the standard curves were constructed and concentrations of the extracted samples were determined from these standard curves. The limit of detection of the microcystins/nodularins ELISA is 0.10 ppb (μg l^-1^), while for cylindrospermopsin is 0.040 ppb (μg l^-1^). ELISA was performed with Araçagi, Saulo Maia, and Mazagão water samples. ELISA analyses were carried out in the Laboratory of Virology (UFRJ).

## Results and Discussion

Chemical and biological parameters segregated the three different ponds (**Figure 1B**). Levels of total nitrogen, Mn, and Ba were higher in Araçagi, whereas Boqueirão was mainly characterized by the higher abundance of total autotrophs and cyanobacteria (Supplementary Figure S1 and Supplementary Table S2). The ponds were hypereutrophic (total phosphate > 0.1 mg l^-1^ and total nitrogen > 0.45 mg l^-1^ for all ponds). The highest phosphorus (0.55 mg l^-1^) and total nitrogen (2.43 mg l^-1^) values were found in Araçagi (Supplementary Table S2). The eutrophic condition in semi-arid regions is established by values above 0.05–0.06 mg l^-1^ of total phosphorus (Thornton and Rast, 1993), and the limit of 0.03 mg l^-1^ of total phosphorus is established by the CONAMA (Resolution 357/05) for Class II lentic environments (reservoirs) – (Brazilian classification of water bodies for human supply established by CONAMA) (Brazil National Environment Council/Conselho Nacional de Meio Ambiente - CONAMA, 2005). High levels of phosphorus and nitrogen promote the formation of blooms of non-diazotrophic cyanobacteria, such as *Microcystis*, and the concomitant production of microcystin, and other secondary metabolites, such as aeruginosin, cyanopeptolin, and protease inhibitors (Gobler et al., 2016; Harke et al., 2016). Protease inhibitors discourage zooplankton grazing, facilitating bloom proliferation (Agrawal et al., 2005; Gobler et al., 2007). The measured nutrient loads clearly demonstrate that ponds are under a severe eutrophication process, possibly conditioned by both climatic factors (e.g., drought) and local pollution. The resulting high loads of nutrients (e.g., phosphorus) may promote the formation of potentially toxic cyanobacterial blooms. However, the mechanisms underlying cyanobacterial bloom formation and the massive toxins production remain to be further investigated (Paerl et al., 2011; Harke et al., 2016; Li et al., 2016).

A total of 8.34 million reads (raw sequences) were obtained for all ponds (Supplementary Table S3). Approximately 1.9 × 10^6^ sequences were annotated. Bacteria domain contributed an average of 81% of the sequences annotated, ranging from 80.6% (Araçagi, total *N* = 397,361), 80.1% (Saulo Maia, total *N* = 223,287), to 77.1% (Boqueirão, total *N* = 382,168) for the major ponds; and 82.8% (*N* = 238,497) to 77.4% (*N* = 207,391) for Galante and Mazagão ponds, respectively. A total of 29 distinct bacterial phyla and 3 candidate bacterial phyla were identified within all samples analyzed. Proteobacteria was the most abundant and largest phylum in all ponds, followed by the unclassified bacteria, Actinobacteria, Bacteroidetes, and Cyanobacteria (Supplementary Figure S2). The phylum Proteobacteria accounted for an average of 34.9% for the three major ponds, ranging from 27.8% (Boqueirão) to 43.8% (Saulo Maia) (Supplementary Figure S2). Bacteroidetes accounted for an average of 12.1%, ranging from 9.3% for Araçagi to 15.9% for Boqueirão, and 10.9% for Saulo Maia. Considering the total of Betaproteobacteria class sequences, the orders *Burkholderiales* (an average of 20.7%) and *Methylophilales* (an average of 5.8%) were the most abundant, and they are recently suggested to be more important in microcystin degradation than *Sphingomonadales*, an Alphaproteobacteria (Mou et al., 2013).

**FIGURE 2:**
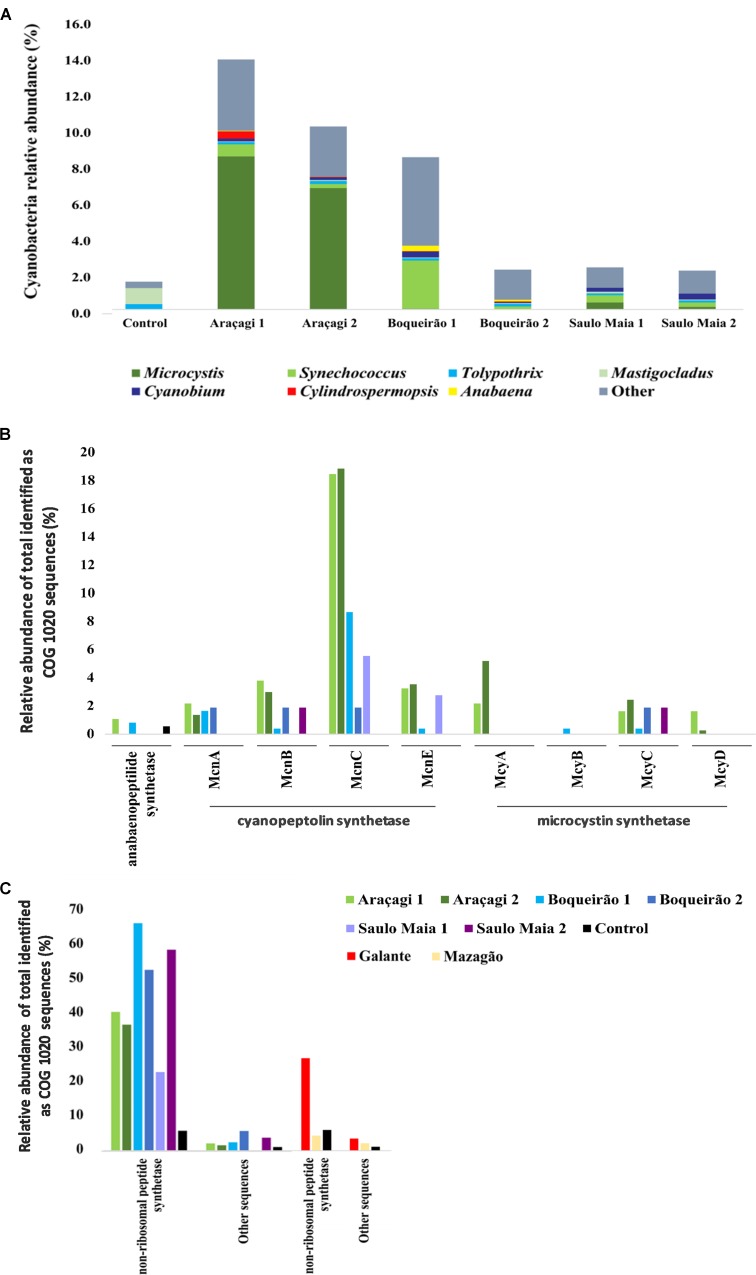
Abundance of sequences related to Cyanobacteria and cyanotoxins. **(A)** The major cyanobacterial genera found in the metagenomes of Araçagi, Boqueirão, and Saulo Maia ponds. **(B,C)** Abundance and distribution of genes involved in the biosynthesis of cyanobacterial toxins found in the metagenomes Values correspond to the relative abundance for the total identified clusters of orthologous groups of proteins sequences (COGs), corresponding to COG 1020 sequences. Microcystin (*mcy*) and cyanopeptolin (*mcn*) synthetase gene clusters were found, as well Anabaenopeptilide/-peptins gene cluster (*apd*) **(B)** and non-ribosomal peptide synthase genes and other genes potentially involved in the biosynthetic pathways for toxins production **(C)**. Araçagi, Boqueirão, and Saulo Maia major ponds presented *mcy*, *mcn*, *apd*, other, and non-ribosomal peptide synthase genes. COG proteins assigned to Anabaenopeptilide/-peptins gene cluster were all annotated as *Anabaena* sp. 90, whereas *Microcystis aeruginosa* was related to cyanopeptolin synthetase gene cluster for all ponds (exception *Planktothrix* spp. for McnC in Boqueirão 1). *M. aeruginosa* was also related to microcystin synthetase gene cluster in all ponds. For non-ribosomal peptide synthetase, the major sequences were related to *M. aeruginosa*, *Nodularia spumigena*, *Aphanizomenon flos-aquae*, *Nostoc* spp., *Microcystis panniformis*, *Planktothrix* spp., *Anabaena* spp., *Fischerella* spp., *Scytonema* spp., and *Chroococcidiopsis thermalis*. Control, bottled mineral water.

The taxonomic assignments of the metagenomic sequences revealed that cyanobacteria contributed an average of 10.6%, ranging from 3.7% in Saulo Maia to 16.2% in Araçagi. Cyanobacteria counts ranged from 2.10 × 10^4^ to 4.46 × 10^5^ cell ml^-1^. Accordingly, Boqueirão pond had the highest cyanobacteria counts and can be classified as Class III, possibly requiring advanced water treatment (CONAMA; Brazil National Environment Council/Conselho Nacional de Meio Ambiente - CONAMA, 2005). *Microcystis* metagenomic sequence counts were approximately 100- and 6-fold more abundant (*p* < 0.01) in Araçagi than in Boqueirão and Saulo Maia, respectively. While the most abundant cyanobacteria belonged to the genus *Microcystis* in Araçagi (an average of 57.6 ± 4.08% of the total cyanobacterial sequences; *N* = 37,204), in Boqueirão, the community was more diverse: *Synechococcus* (an average of 13.8 ± 8.8%, *N* = 9,456), *Anabaena* (an average of 3 ± 0.4%, *N* = 1,219), *Cyanobium* (an average of 2.3 ± 0.45%, *N* = 1,210), and *Cylindrospermopsis* (an average of 0.6%, *N* = 127). Unclassified cyanobacteria are also an abundant group in the ponds, accounting for an average of 34.1 ± 12.4% (*N* = 15,483) in Boqueirão, 29 ± 1.8% (*N* = 2,429) in Saulo Maia, and 20.1 ± 3.64% (*N* = 12,978) in Araçagi, considering the total of Cyanobacteria. COG functional annotation of metagenomic sequences revealed gene sequences related to toxin production in all ponds, including cyanopeptolin synthetase (*mcn*), microcystin synthetase (*mcy*), and non-ribosomal peptide synthase genes (*N* = 688, ranging from 280 in Araçagi to 11 in Saulo Maia; **Figures 2B,C**; Supplementary Table S4). Not all *mcn* and *mcy* produce toxins, although their presence in the metagenomes hints to the toxicity potential of pond waters. To evaluate the potential toxicity of pond water, we went further and investigated the presence of toxins by ELISA and tested water toxicity in a standard zebrafish embryo model under controlled laboratory conditions.

Microcystins, nodularins, and cylindrospermopsins were detected by ELISA in small concentrations in all samples (Supplementary Table S5). Araçagi had the highest toxin concentration (0.5 ± 0.2 μg l^-1^ microcystins/nodularins and 0.08 μg l^-1^ for cylindrospermopsin). Toxin levels were similar to those obtained in previous studies conducted in Rio Grande do Norte (Fonseca et al., 2015), and in Pernambuco (Piccin-Santos and Bittencourt-Oliveira, 2012). These levels were below the threshold for human consumption (<1 μg l^-1^), proposed by the World Health Organization (Chorus and Bartram, 1999), and followed by the Brazilian Ministry of Health (Ordinance 2914/2011) (Brazil Ministry of Health/Ministério da Saúde, 2011). Given that ELISA kits used cross-react only against eight microcystin congeners/isoforms (Microcystin-LA, Microcystin-LF, Microcystin-LR, Microcystin-LW, Microcystin-LY, Microcystin-RR, and Microcystin-YR), and one for nodularin (Nodularin-R), the values obtained here could be underestimates of the real toxin concentrations in the pond waters. There is an estimative of more than 80 other microcystin congeners being largely disregarded in ready-to-use ELISA kits (Dietrich and Hoeger, 2005). In addition, the sample treatment, preparation, and storage prior to cyanotoxins analysis could also affect results, leading, for example, to low recoveries of microcystin concentrations in the order of 40–70% (Kamp et al., 2016). Nevertheless, the detection of toxins in the Campina Grande ponds hints to the risk of water consumption prior treatment and the need for reliable water quality monitoring programs in this region. The need for a reliable governance program is even more evident by the zebrafish toxicological results obtained here.

**FIGURE 3:**
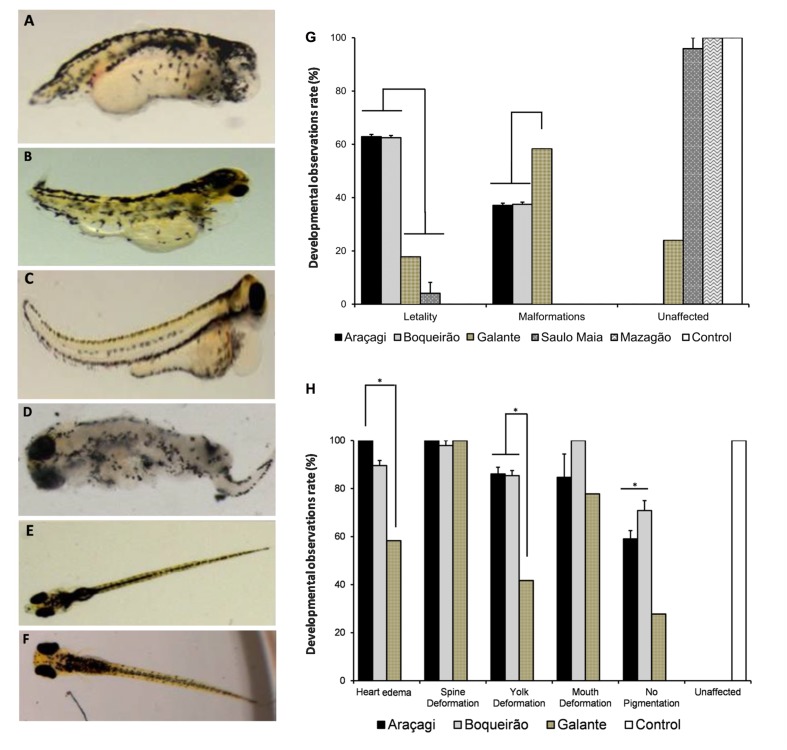
Environmental water ponds induce different developmental defects in zebrafish. Teratogenic effects were observed at 96 and 120 hour post-fertilization (hpf) (*N* = 60 embryos for each treatment). **(A,B)** Developmental delays; malformations of the head, mouth, jaw, spine, and tail; and heart edema were observed in zebrafish treated with Araçagi water samples. **(C,D)** Developmental delays and malformations of the spine, tail, tail curving, heart edema, and deterioration were observed in zebrafish treated with Boqueirão water samples. **(E,F)** Normal development observed in zebrafish controls. **(G)** Developmental observations rate in zebrafish. Unaffected and teratogenic rates observed in embryos treated with environmental water samples. Most embryos from Saulo Maia were unaffected, while most embryos from Araçagi and Boqueirão presented lethality, followed by different malformations. **(H)** Developmental observation rate of teratogenic effects in Araçagi and Boqueirão.

The zebrafish embryo lethality rate was significantly higher (*p* < 0.01) in Araçagi (62.9 ± 0.8%) and Boqueirão (62.5 ± 0.8%) than in Saulo Maia waters (8.2 ± 1.0%) (**Figure 3**). Zebrafish malformations were found in Boqueirão (37.5 ± 0.8%) and Araçagi (37.1 ± 0.8%). Water from Saulo Maia did not result in zebrafish malformations (**Figure 3G**). Whereas 100% of the embryos presented heart edema and spine deformation when reared with Araçagi water; 100% of the embryos presented mouth deformations when reared with Boqueirão water (**Figure 3**). Water quality monitoring programs that evaluate toxin presence and toxicity are lacking in the semi-arid region studied here. Araçagi and Boqueirão waters were the most lethal and toxic to zebrafish (**Figures 3A,B** and **C,D** respectively), whereas the controls did not show any evidence of lethality and toxicity (**Figures 3E,F**). The observed diverse malformations and killing in the present study are in agreement with previous studies (Adámek et al., 2011; Dao et al., 2013; Pavagadhi et al., 2013). The observed effects include a variety of toxic cellular actions typical of microcystins, e.g., DNA damage, mitochondria dysfunction, endoplasmic reticulum disturbance, and cell cycle deregulation, all contributing to apoptosis/programmed cell death of hepatocytes as well as many other cell types (Chen and Xie, 2016) (Supplementary Figure S3). Microcystins have been implicated in neurotoxicity, hepatotoxicity, and damage to reproductive organs (Lone et al., 2015; Máthé, 2016; Valério et al., 2017). These cyanotoxins are potent inhibitors of protein phosphatases (phosphatase 1 and phosphatase 2A), which are key regulators of embryonic development, leading to changes in mRNA levels of genes that induce oxidative stress (endoplasmic reticulum stress) involving reactive oxygen species (ROS) generation in zebrafish (Faltermann et al., 2016).

## Concluding Remarks

Safe water supply for human consumption remains a challenge task in the northeast Brazil. Our comprehensive approach integrating metagenomics, biogeochemical analysis, and toxicity tests clearly demonstrates that the untreated pond water from semi-arid regions is not a safe source of drinking water. The toxicity and teratogenicity of pond water observed in the present study hints to possible harmful effects in human health. Finally, we highlight that reliable water quality monitoring may be an important tool to improve water management and governance.

## Author Contributions

JW, FL, ML-F, LV, LL, FM, GA, RO, AM, ASOM, CER, AT, and FT designed and planned the study. JW, FL, LV, and RO carried out the field work. JW and FL performed the bioinformatics analyses. JW, FL, ML-F, and LV compiled the data. JW, FL, ML-F, AM, ASOM, CER, AT, and FT analyzed the results. JW, FL, ML-F, and FT wrote the manuscript and all authors commented on the manuscript.

## Conflict of Interest Statement

The authors declare that the research was conducted in the absence of any commercial or financial relationships that could be construed as a potential conflict of interest. FT is a member of Frontiers Editorial Board.

## Footnotes

1 http://www.who.int/mediacentre/factsheets/fs391/en/

2 ftp://ftp.ncbi.nlm.nih.gov/blast/db/FASTA/nr.gz

3 http://www.ncbi.nlm.nih.gov/RefSeq/
