# Supplementary material for: Occurrence of Harmful Cyanobacteria in Drinking Water from a Severely Drought-Impacted Semi-arid Region
Source: Front Microbiol. 2018 Feb 28;9:176. doi: 10.3389/fmicb.2018.00176 (PMC5835534; doi:10.3389/fmicb.2018.00176)

**Supplementary Information**

**“Occurrence of harmful cyanobacteria in drinking water from a severely drought-impacted semi-arid region”**

**Supporting Figures and Tables**

**Supporting Table 1.** Details of the samples collected. Nm, not measured.

| **Samples** | **Latitude** | **Longitude** | **Temp. (°C)** | **DO (mg.L^-1^)** | **pH** |
| --- | --- | --- | --- | --- | --- |
| Araçagi | -6.8526 | -35.295 | 26.8 ± 2 | 6.39 | 6.5 |
| Boqueirão | -7.4975 | -36.145 | 25.4 ± 2 | 9.82 | 6.1 |
| Saulo Maia | -6.942 | -35.679 | 26.7 ± 2 | 7.13 | 6.5 |
| Galante | -7.3113 | -35.778 | nm | nm | nm |
| Mazagão | -6.9737 | -35.710 | 30.5 ± 2 | 8.77 | 5.5 |

**Supporting Table 2.** Chemical and biological parameters for the inland water studied. Mean (± Standard Deviation; N = 3).

| **Samples** | **Date** | **Total Autotroph**    **(cells.mL^-1^)** | | | **Cyanobacteria**    **(cells.mL^-1^)** | | | **COD** | | **TN** | | **P** | | **S** | | **Fe** | | **Al** | | **Mn** | | **Ba** | | **Hg** | |  |
| --- | --- | --- | --- | --- | --- | --- | --- | --- | --- | --- | --- | --- | --- | --- | --- | --- | --- | --- | --- | --- | --- | --- | --- | --- | --- | --- |
|  |  |  |  |  |  |  |  | **(mg.L^-1^)** | | **(mg.L^-1^)** | | **(mg.L^-1^)** | | **(mg.L^-1^)** | | **(mg.L^-1^)** | | **(mg.L^-1^)** | | **(mg.L^-1^)** | | **(mg.L^-1^)** | | **(µg.L^-1^)** | |  |
| Araçagi 1 | 21 Sept 2016 | 3.40E+05 | ± | 2.6E+04 | 1.68E+05 | ± | 1.3E+04 | | 0.8015 | | 2.431 | | 0.558 | | 0.788 | | <0.007 | | <0.007 | | 0.030 | | 0.080 | | <0.00001 | |
| Araçagi 2 | 29 Oct 2016 | 1.14E+05 | ± | 1.4E+04 | 2.32E+04 | ± | 3.7E+03 | | 21.89 | | 0.8463 | | 0.134 | | 3.807 | | <0,007 | | 0.016 | | 0.010 | | 0.116 | | <0.00001 | |
| Boqueirão 1 | 22 Sept 2016 | 4.41E+05 | ± | 6.9E+04 | 4.46E+05 | ± | 9.0E+04 | | 17.42 | | 0.4947 | | 0.326 | | 8.254 | | 0.026 | | 0.323 | | <0,003 | | 0.204 | | 0.00091 | |
| Boqueirão 2 | 30 Oct 2016 | 1.05E+05 | ± | 2.9E+03 | 2.10E+04 | ± | 8.0E+02 | | 31.84 | | 0.9403 | | 0.077 | | 7.690 | | 0.026 | | 0.048 | | <0,003 | | 0.200 | | <0.00001 | |
| Saulo Maia 1 | 23 Sept 2016 | 2.20E+05 | ± | 1.9E+04 | 1.73E+05 | ± | 1.9E+04 | | 17.86 | | 0.4646 | | 0.303 | | 2.752 | | 0.048 | | 0.050 | | <0,003 | | 0.049 | | <0.00001 | |
| Saulo Maia 2 | 31 Oct 2016 | 1.70E+05 | ± | 1.0E+04 | 9.80E+04 | ± | 9.5E+03 | | 19.61 | | 0.5965 | | 0.133 | | 2.963 | | 0.030 | | 0.045 | | <0,003 | | 0.034 | | <0.00001 | |
| Galante | 30 Oct 2016 | 4.62E+05 | ± | 2.2E+04 | 1.67E+05 | ± | 9.9E+03 | | 35.09 | | 1.742 | | 0.149 | | 1.18 | | 0.001 | | 0.028 | | 0.027 | | 0.648 | | 0.0009 | |
| Mazagão | 24 Sept 2016 | 7.64E+05 | ± | 2.5E+04 | 3.32E+05 | ± | 1.1E+05 | | 17.11 | | 0.472 | | 0.345 | | 14.294 | | 0.837 | | 0.069 | | 0.004 | | 0.070 | | 0.00016 | |

All values for Ti (mg.L^-1^) were <0.007; all values for Ni, Pb, Cr and Cu (mg.L^-1^) were <0.004; all values for Cd (mg.L^-1^) were <0.001; all values for Co (mg.L^-1^) were <0.005; all values for As and Se (mg.L^-1^) were <0.009; all values for Zn (mg.L^-1^) were <0.003 with exception of Galante (0.00404 mg.L^-1^).

**Supporting Table 3.** General features of all metagenomes. A total of 8.34 million reads (raw sequences) were obtained for all ponds. After quality control, the metagenomes contained from 146,000 to 1,310,000 reads each. Approximately 1.9×10^6^ sequences were annotated for Bacteria, Archaea, and Eukarya domains, and viruses.

| **Metagenomes** | **Before QC process** | **After QC process** | **Number of sequences assigned** | **Bacteria (number of sequences)** | **Archaea (number of sequences)** | **Eukaryota (number of sequences)** | **Viruses (number of sequences)** |
| --- | --- | --- | --- | --- | --- | --- | --- |
| Control | 7.77E+05 | 7.33E+05 | 2.84E+05 | 2.78E+05 | 7.43E+02 | 5.62E+03 | 1.86E+02 |
| Aracagi 1 | 7.70E+05 | 7.17E+05 | 1.67E+05 | 1.59E+05 | 2.26E+02 | 4.20E+03 | 3.41E+03 |
| Aracagi 2 | 1.39E+06 | 1.31E+06 | 2.65E+05 | 2.38E+05 | 3.90E+02 | 1.96E+04 | 7.27E+03 |
| Boqueirão 1 | 1.25E+06 | 1.17E+06 | 2.86E+05 | 2.68E+05 | 2.69E+02 | 5.78E+03 | 1.20E+04 |
| Boqueirão 2 | 7.07E+05 | 6.55E+05 | 1.40E+05 | 1.14E+05 | 3.07E+02 | 1.04E+04 | 1.59E+04 |
| Saulo Maia 1 | 9.72E+05 | 8.96E+05 | 2.10E+05 | 1.98E+05 | 2.40E+02 | 4.41E+03 | 7.27E+03 |
| Saulo Maia 2 | 1.54E+05 | 1.46E+05 | 2.87E+04 | 2.48E+04 | 8.00E+01 | 2.33E+03 | 1.42E+03 |
| Galante | 9.76E+05 | 8.95E+05 | 2.47E+05 | 2.38E+05 | 5.48E+02 | 4.91E+03 | 3.55E+03 |
| Mazagão | 1.35E+06 | 1.23E+06 | 2.33E+05 | 2.07E+05 | 5.65E+02 | 1.06E+04 | 1.45E+04 |
| Total | 8.34E+06 | 7.75E+06 | 1.86E+06 | 1.73E+06 | 3.37E+03 | 6.79E+04 | 6.55E+04 |

**Supporting Table 4.** List of sequences related to the biosynthesis of cyanotoxins.

**Pond Subject id Nº. Description / Organism**

Araçagi gi|640535887|ref|WP_024969393.1| 1 non-ribosomal peptide synthetase [Microcystis aeruginosa]

Araçagi gi|488839880|ref|WP_002752286.1| 1 non-ribosomal peptide synthetase [Microcystis aeruginosa]

Araçagi gi|754792453|ref|WP_042156032.1| 1 non-ribosomal peptide synthetase [Planktothrix agardhii]

Araçagi gi|652399811|ref|WP_026795613.1| 1 MULTISPECIES: non-ribosomal peptide synthetase [Planktothrix]

Araçagi gi|653003254|ref|WP_027255440.1| 1 non-ribosomal peptide synthetase [Planktothrix agardhii]

Araçagi gi|1105146240|ref|WP_071823439.1| 1 hypothetical protein [Microcystis aeruginosa]

Araçagi gi|917764583|ref|WP_052278588.1| 1 hypothetical protein [Microcystis panniformis]

Araçagi gi|1112924879|ref|WP_071995597.1| 1 hypothetical protein [Microcystis aeruginosa]

Araçagi gi|738448646|ref|WP_036399833.1| 1 McnB protein [Microcystis aeruginosa]

Araçagi gi|488874708|ref|WP_002786933.1| 1 non-ribosomal peptide synthetase [Microcystis aeruginosa]

Araçagi gi|488864797|ref|WP_002777036.1| 1 McnC protein [Microcystis aeruginosa]

Araçagi gi|488864797|ref|WP_002777036.1| 1 McnC protein [Microcystis aeruginosa]

Araçagi gi|488864797|ref|WP_002777036.1| 1 McnC protein [Microcystis aeruginosa]

Araçagi gi|488864797|ref|WP_002777036.1| 1 McnC protein [Microcystis aeruginosa]

Araçagi gi|488879470|ref|WP_002791695.1| 1 non-ribosomal peptide synthetase [Microcystis aeruginosa]

Araçagi gi|488879470|ref|WP_002791695.1| 1 non-ribosomal peptide synthetase [Microcystis aeruginosa]

Araçagi gi|488879470|ref|WP_002791695.1| 1 non-ribosomal peptide synthetase [Microcystis aeruginosa]

Araçagi gi|488879470|ref|WP_002791695.1| 1 non-ribosomal peptide synthetase [Microcystis aeruginosa]

Araçagi gi|817700114|ref|WP_046662633.1| 1 McnC protein [Microcystis aeruginosa]

Araçagi gi|817700114|ref|WP_046662633.1| 1 McnC protein [Microcystis aeruginosa]

Araçagi gi|817700114|ref|WP_046662633.1| 1 McnC protein [Microcystis aeruginosa]

Araçagi gi|817700114|ref|WP_046662633.1| 1 McnC protein [Microcystis aeruginosa]

Araçagi gi|653003249|ref|WP_027255435.1| 1 McnC protein [Planktothrix agardhii]

Araçagi gi|653003249|ref|WP_027255435.1| 1 McnC protein [Planktothrix agardhii]

Araçagi gi|653003249|ref|WP_027255435.1| 1 McnC protein [Planktothrix agardhii]

Araçagi gi|653003249|ref|WP_027255435.1| 1 McnC protein [Planktothrix agardhii]

Araçagi gi|652389981|ref|WP_026785829.1| 1 McnC protein [Planktothrix rubescens]

Araçagi gi|652389981|ref|WP_026785829.1| 1 McnC protein [Planktothrix rubescens]

Araçagi gi|652389981|ref|WP_026785829.1| 1 McnC protein [Planktothrix rubescens]

Araçagi gi|652389981|ref|WP_026785829.1| 1 McnC protein [Planktothrix rubescens]

Araçagi gi|488829433|ref|WP_002741839.1| 1 McnE protein [Microcystis aeruginosa]

Araçagi gi|640535235|ref|WP_024968867.1| 1 non-ribosomal peptide synthetase [Microcystis aeruginosa]

Araçagi gi|488838375|ref|WP_002750781.1| 1 non-ribosomal peptide synthetase [Microcystis aeruginosa]

Araçagi gi|488864795|ref|WP_002777034.1| 1 non-ribosomal peptide synthetase [Microcystis aeruginosa]

Araçagi gi|488874710|ref|WP_002786935.1| 1 non-ribosomal peptide synthetase [Microcystis aeruginosa]

Araçagi gi|501225152|ref|WP_012268170.1| 1 non-ribosomal peptide synthetase [Microcystis aeruginosa]

Araçagi gi|1002985853|ref|WP_061430380.1| 1 non-ribosomal peptide synthetase [Microcystis aeruginosa]

Araçagi gi|488864795|ref|WP_002777034.1| 1 non-ribosomal peptide synthetase [Microcystis aeruginosa]

Araçagi gi|490389196|ref|WP_004268651.1| 1 non-ribosomal peptide synthetase [Microcystis aeruginosa]

Araçagi gi|640535235|ref|WP_024968867.1| 1 non-ribosomal peptide synthetase [Microcystis aeruginosa]

Araçagi gi|763117447|ref|WP_043997361.1| 1 McyA protein [Microcystis aeruginosa]

Araçagi gi|763117447|ref|WP_043997361.1| 1 McyA protein [Microcystis aeruginosa]

Araçagi gi|738446319|ref|WP_036397508.1| 1 McyA protein [Microcystis aeruginosa]

Araçagi gi|738446319|ref|WP_036397508.1| 1 McyA protein [Microcystis aeruginosa]

Araçagi gi|488880990|ref|WP_002793215.1| 1 Microcystin synthetase A [Microcystis aeruginosa]

Araçagi gi|488880990|ref|WP_002793215.1| 1 Microcystin synthetase A [Microcystis aeruginosa]

Araçagi gi|1002987251|ref|WP_061431778.1| 1 non-ribosomal peptide synthetase [Microcystis aeruginosa]

Araçagi gi|1002987251|ref|WP_061431778.1| 1 non-ribosomal peptide synthetase [Microcystis aeruginosa]

Araçagi gi|488884831|ref|WP_002797056.1| 1 McyA protein [Microcystis aeruginosa]

Araçagi gi|488884831|ref|WP_002797056.1| 1 McyA protein [Microcystis aeruginosa]

Araçagi gi|1002987251|ref|WP_061431778.1| 1 non-ribosomal peptide synthetase [Microcystis aeruginosa]

Araçagi gi|1120049983|ref|WP_072923806.1| 1 non-ribosomal peptide synthetase [Microcystis aeruginosa]

Araçagi gi|917763913|ref|WP_052277918.1| 1 McyA protein [Microcystis panniformis]

Araçagi gi|763117447|ref|WP_043997361.1| 1 McyA protein [Microcystis aeruginosa]

Araçagi gi|738446319|ref|WP_036397508.1| 1 McyA protein [Microcystis aeruginosa]

Araçagi gi|490265524|ref|WP_004162176.1| 1 non-ribosomal peptide synthetase [Microcystis aeruginosa]

Araçagi gi|1056982475|ref|WP_068394500.1| 1 hypothetical protein [Leptolyngbya sp. NIES-3755]

Araçagi gi|501601191|ref|WP_012599719.1| 1 non-ribosomal peptide synthetase [Cyanothece sp. PCC 7424]

Araçagi gi|959923568|ref|WP_058182935.1| 1 non-ribosomal peptide synthetase [Mastigocoleus testarum]

Araçagi gi|504991553|ref|WP_015178655.1| 1 non-ribosomal peptide synthetase [Oscillatoria nigro-viridis]

Araçagi gi|740233608|ref|WP_038074805.1| 1 non-ribosomal peptide synthetase [Tolypothrix bouteillei]

Araçagi gi|740233608|ref|WP_038074805.1| 1 non-ribosomal peptide synthetase [Tolypothrix bouteillei]

Araçagi gi|515383523|ref|WP_016876762.1| 1 non-ribosomal peptide synthetase [Chlorogloeopsis fritschii]

Araçagi gi|516355639|ref|WP_017745672.1| 1 non-ribosomal peptide synthetase [Scytonema hofmannii]

Araçagi gi|516355639|ref|WP_017745672.1| 1 non-ribosomal peptide synthetase [Scytonema hofmannii]

Araçagi gi|501222817|ref|WP_012265835.1| 1 peptide synthetase [Microcystis aeruginosa]

Araçagi gi|779876898|ref|WP_045359195.1| 1 peptide synthetase [Microcystis aeruginosa]

Araçagi gi|917763686|ref|WP_052277691.1| 1 MULTISPECIES: peptide synthetase [Microcystis]

Araçagi gi|488877749|ref|WP_002789974.1| 1 peptide synthetase [Microcystis aeruginosa]

Araçagi gi|488849510|ref|WP_002761916.1| 1 peptide synthetase [Microcystis aeruginosa]

Araçagi gi|652402539|ref|WP_026798334.1| 1 non-ribosomal peptide synthetase [Planktothrix prolifica]

Araçagi gi|488893005|ref|WP_002804150.1| 1 MicD protein [Microcystis aeruginosa]

Araçagi gi|1065018842|ref|WP_069475190.1| 1 non-ribosomal peptide synthetase [Microcystis aeruginosa]

Araçagi gi|488861780|ref|WP_002774019.1| 1 MicD protein [Microcystis aeruginosa]

Araçagi gi|488839871|ref|WP_002752277.1| 1 MicD protein [Microcystis aeruginosa]

Araçagi gi|1105152502|ref|WP_071823928.1| 1 hypothetical protein [Microcystis aeruginosa]

Araçagi gi|740247273|ref|WP_038088226.1| 1 non-ribosomal peptide synthetase [Tolypothrix bouteillei]

Araçagi gi|652326501|ref|WP_026723819.1| 1 non-ribosomal peptide synthase [Fischerella sp. PCC 9431]

Araçagi gi|916863124|ref|WP_051470180.1| 1 amino acid adenylation protein [Fischerella sp. PCC 9605]

Araçagi gi|1121316969|ref|WP_073627108.1| 1 hypothetical protein [Scytonema sp. HK-05]

Araçagi gi|1121316969|ref|WP_073627108.1| 1 hypothetical protein [Scytonema sp. HK-05]

Araçagi gi|1121316969|ref|WP_073627108.1| 1 hypothetical protein [Scytonema sp. HK-05]

Araçagi gi|488829431|ref|WP_002741837.1| 1 McnC protein [Microcystis aeruginosa]

Araçagi gi|488829431|ref|WP_002741837.1| 1 McnC protein [Microcystis aeruginosa]

Araçagi gi|488829431|ref|WP_002741837.1| 1 McnC protein [Microcystis aeruginosa]

Araçagi gi|488829431|ref|WP_002741837.1| 1 McnC protein [Microcystis aeruginosa]

Araçagi gi|501225153|ref|WP_012268171.1| 1 McnC protein [Microcystis aeruginosa]

Araçagi gi|501225153|ref|WP_012268171.1| 1 McnC protein [Microcystis aeruginosa]

Araçagi gi|501225153|ref|WP_012268171.1| 1 McnC protein [Microcystis aeruginosa]

Araçagi gi|501225153|ref|WP_012268171.1| 1 McnC protein [Microcystis aeruginosa]

Araçagi gi|1119342681|ref|WP_072319556.1| 1 hypothetical protein [Microcystis aeruginosa]

Araçagi gi|1119342681|ref|WP_072319556.1| 1 hypothetical protein [Microcystis aeruginosa]

Araçagi gi|488879470|ref|WP_002791695.1| 1 non-ribosomal peptide synthetase [Microcystis aeruginosa]

Araçagi gi|488879470|ref|WP_002791695.1| 1 non-ribosomal peptide synthetase [Microcystis aeruginosa]

Araçagi gi|488879470|ref|WP_002791695.1| 1 non-ribosomal peptide synthetase [Microcystis aeruginosa]

Araçagi gi|488879470|ref|WP_002791695.1| 1 non-ribosomal peptide synthetase [Microcystis aeruginosa]

Araçagi gi|640535236|ref|WP_024968868.1| 1 McnC protein [Microcystis aeruginosa]

Araçagi gi|640535236|ref|WP_024968868.1| 1 McnC protein [Microcystis aeruginosa]

Araçagi gi|640535236|ref|WP_024968868.1| 1 McnC protein [Microcystis aeruginosa]

Araçagi gi|640535236|ref|WP_024968868.1| 1 McnC protein [Microcystis aeruginosa]

Araçagi gi|505001101|ref|WP_015188203.1| 1 non-ribosomal peptide synthetase [Gloeocapsa sp. PCC 7428]

Araçagi gi|504968607|ref|WP_015155709.1| 1 non-ribosomal peptide synthetase [Chroococcidiopsis thermalis]

Araçagi gi|501377949|ref|WP_012409515.1| 1 non-ribosomal peptide synthetase [Nostoc punctiforme]

Araçagi gi|501377949|ref|WP_012409515.1| 1 non-ribosomal peptide synthetase [Nostoc punctiforme]

Araçagi gi|501377949|ref|WP_012409515.1| 1 non-ribosomal peptide synthetase [Nostoc punctiforme]

Araçagi gi|1060047733|ref|WP_069074351.1| 1 non-ribosomal peptide synthetase [Nostoc sp. KVJ20]

Araçagi gi|1054614249|ref|WP_066382131.1| 1 hypothetical protein [Anabaena sp. CA = ATCC 33047]

Araçagi gi|490265530|ref|WP_004162182.1| 1 non-ribosomal peptide synthetase [Microcystis aeruginosa]

Araçagi gi|916599645|ref|WP_051206736.1| 1 hypothetical protein [Fischerella sp. PCC 9431]

Araçagi gi|654347244|ref|WP_027840649.1| 1 hypothetical protein [Mastigocoleus testarum]

Araçagi gi|488828768|ref|WP_002741174.1| 1 non-ribosomal peptide synthetase [Microcystis aeruginosa]

Araçagi gi|1120051153|ref|WP_072924976.1| 1 non-ribosomal peptide synthetase [Microcystis aeruginosa]

Araçagi gi|488876300|ref|WP_002788525.1| 1 non-ribosomal peptide synthetase [Microcystis aeruginosa]

Araçagi gi|488881832|ref|WP_002794057.1| 1 non-ribosomal peptide synthetase [Microcystis aeruginosa]

Araçagi gi|1065016475|ref|WP_069474085.1| 1 non-ribosomal peptide synthetase [Microcystis aeruginosa]

Araçagi gi|738448646|ref|WP_036399833.1| 1 McnB protein [Microcystis aeruginosa]

Araçagi gi|488874708|ref|WP_002786933.1| 1 non-ribosomal peptide synthetase [Microcystis aeruginosa]

Araçagi gi|1119342681|ref|WP_072319556.1| 1 hypothetical protein [Microcystis aeruginosa]

Araçagi gi|1119342681|ref|WP_072319556.1| 1 hypothetical protein [Microcystis aeruginosa]

Araçagi gi|1119342681|ref|WP_072319556.1| 1 hypothetical protein [Microcystis aeruginosa]

Araçagi gi|640537447|ref|WP_024970455.1| 1 McnB protein

Araçagi gi|763118395|ref|WP_043998309.1| 1 McnB protein

Araçagi gi|1120051178|ref|WP_072925001.1| 1 peptide synthetase [Microcystis aeruginosa]

Araçagi gi|917761359|ref|WP_052275364.1| 1 peptide synthetase [Microcystis panniformis]

Araçagi gi|488853368|ref|WP_002765679.1| 1 peptide synthetase [Microcystis aeruginosa]

Araçagi gi|488828765|ref|WP_002741171.1| 1 AMP-binding protein [Microcystis aeruginosa]

Araçagi gi|1002987897|ref|WP_061432424.1| 1 peptide synthetase [Microcystis aeruginosa]

Araçagi gi|488828768|ref|WP_002741174.1| 1 non-ribosomal peptide synthetase [Microcystis aeruginosa]

Araçagi gi|1120051153|ref|WP_072924976.1| 1 non-ribosomal peptide synthetase [Microcystis aeruginosa]

Araçagi gi|501224905|ref|WP_012267923.1| 1 non-ribosomal peptide synthetase [Microcystis aeruginosa]

Araçagi gi|1002987895|ref|WP_061432422.1| 1 non-ribosomal peptide synthetase [Microcystis aeruginosa]

Araçagi gi|488876300|ref|WP_002788525.1| 1 non-ribosomal peptide synthetase [Microcystis aeruginosa]

Araçagi gi|1094117065|ref|WP_071106564.1| 1 non-ribosomal peptide synthetase [Moorea producens]

Araçagi gi|504959837|ref|WP_015146939.1| 1 non-ribosomal peptide synthase [Oscillatoria acuminata]

Araçagi gi|493684185|ref|WP_006634319.1| 1 non-ribosomal peptide synthase [Microcoleus vaginatus]

Araçagi gi|817700115|ref|WP_046662634.1| 1 non-ribosomal peptide synthetase [Microcystis aeruginosa]

Araçagi gi|488864795|ref|WP_002777034.1| 1 non-ribosomal peptide synthetase [Microcystis aeruginosa]

Araçagi gi|501225152|ref|WP_012268170.1| 1 non-ribosomal peptide synthetase [Microcystis aeruginosa]

Araçagi gi|490389196|ref|WP_004268651.1| 1 non-ribosomal peptide synthetase [Microcystis aeruginosa]

Araçagi gi|1002985853|ref|WP_061430380.1| 1 non-ribosomal peptide synthetase [Microcystis aeruginosa]

Araçagi gi|640535888|ref|WP_024969394.1| 1 non-ribosomal peptide synthetase [Microcystis aeruginosa]

Araçagi gi|488839879|ref|WP_002752285.1| 1 non-ribosomal peptide synthetase [Microcystis aeruginosa]

Araçagi gi|488893012|ref|WP_002804157.1| 1 non-ribosomal peptide synthetase [Microcystis aeruginosa]

Araçagi gi|652996395|ref|WP_027248862.1| 1 non-ribosomal peptide synthetase [Planktothrix agardhii]

Araçagi gi|754792454|ref|WP_042156033.1| 1 non-ribosomal peptide synthetase [Planktothrix agardhii]

Araçagi gi|488829431|ref|WP_002741837.1| 1 McnC protein [Microcystis aeruginosa]

Araçagi gi|488829431|ref|WP_002741837.1| 1 McnC protein [Microcystis aeruginosa]

Araçagi gi|488829431|ref|WP_002741837.1| 1 McnC protein [Microcystis aeruginosa]

Araçagi gi|488829431|ref|WP_002741837.1| 1 McnC protein [Microcystis aeruginosa]

Araçagi gi|1002985852|ref|WP_061430379.1| 1 non-ribosomal peptide synthetase [Microcystis aeruginosa]

Araçagi gi|1002985852|ref|WP_061430379.1| 1 non-ribosomal peptide synthetase [Microcystis aeruginosa]

Araçagi gi|1002985852|ref|WP_061430379.1| 1 non-ribosomal peptide synthetase [Microcystis aeruginosa]

Araçagi gi|1002985852|ref|WP_061430379.1| 1 non-ribosomal peptide synthetase [Microcystis aeruginosa]

Araçagi gi|1120050422|ref|WP_072924245.1| 1 non-ribosomal peptide synthetase [Microcystis aeruginosa]

Araçagi gi|1120050422|ref|WP_072924245.1| 1 non-ribosomal peptide synthetase [Microcystis aeruginosa]

Araçagi gi|1120050422|ref|WP_072924245.1| 1 non-ribosomal peptide synthetase [Microcystis aeruginosa]

Araçagi gi|1120050422|ref|WP_072924245.1| 1 non-ribosomal peptide synthetase [Microcystis aeruginosa]

Araçagi gi|817700114|ref|WP_046662633.1| 1 McnC protein [Microcystis aeruginosa]

Araçagi gi|817700114|ref|WP_046662633.1| 1 McnC protein [Microcystis aeruginosa]

Araçagi gi|817700114|ref|WP_046662633.1| 1 McnC protein [Microcystis aeruginosa]

Araçagi gi|817700114|ref|WP_046662633.1| 1 McnC protein [Microcystis aeruginosa]

Araçagi gi|501225153|ref|WP_012268171.1| 1 McnC protein [Microcystis aeruginosa]

Araçagi gi|501225153|ref|WP_012268171.1| 1 McnC protein [Microcystis aeruginosa]

Araçagi gi|501225153|ref|WP_012268171.1| 1 McnC protein [Microcystis aeruginosa]

Araçagi gi|501225153|ref|WP_012268171.1| 1 McnC protein [Microcystis aeruginosa]

Araçagi gi|754792450|ref|WP_042156029.1| 1 non-ribosomal peptide synthetase [Planktothrix agardhii]

Araçagi gi|488893016|ref|WP_002804161.1| 1 non-ribosomal peptide synthetase [Microcystis aeruginosa]

Araçagi gi|652399809|ref|WP_026795611.1| 1 MULTISPECIES: non-ribosomal peptide synthetase [Planktothrix]

Araçagi gi|653003256|ref|WP_027255442.1| 1 non-ribosomal peptide synthetase [Planktothrix agardhii]

Araçagi gi|488839883|ref|WP_002752289.1| 1 non-ribosomal peptide synthetase [Microcystis aeruginosa]

Araçagi gi|1002985852|ref|WP_061430379.1| 1 non-ribosomal peptide synthetase [Microcystis aeruginosa]

Araçagi gi|1002985852|ref|WP_061430379.1| 1 non-ribosomal peptide synthetase [Microcystis aeruginosa]

Araçagi gi|1002985852|ref|WP_061430379.1| 1 non-ribosomal peptide synthetase [Microcystis aeruginosa]

Araçagi gi|1002985852|ref|WP_061430379.1| 1 non-ribosomal peptide synthetase [Microcystis aeruginosa]

Araçagi gi|501225153|ref|WP_012268171.1| 1 McnC protein [Microcystis aeruginosa]

Araçagi gi|501225153|ref|WP_012268171.1| 1 McnC protein [Microcystis aeruginosa]

Araçagi gi|501225153|ref|WP_012268171.1| 1 McnC protein [Microcystis aeruginosa]

Araçagi gi|501225153|ref|WP_012268171.1| 1 McnC protein [Microcystis aeruginosa]

Araçagi gi|488829431|ref|WP_002741837.1| 1 McnC protein [Microcystis aeruginosa]

Araçagi gi|488829431|ref|WP_002741837.1| 1 McnC protein [Microcystis aeruginosa]

Araçagi gi|488829431|ref|WP_002741837.1| 1 McnC protein [Microcystis aeruginosa]

Araçagi gi|488829431|ref|WP_002741837.1| 1 McnC protein [Microcystis aeruginosa]

Araçagi gi|640535236|ref|WP_024968868.1| 1 McnC protein [Microcystis aeruginosa]

Araçagi gi|640535236|ref|WP_024968868.1| 1 McnC protein [Microcystis aeruginosa]

Araçagi gi|640535236|ref|WP_024968868.1| 1 McnC protein [Microcystis aeruginosa]

Araçagi gi|640535236|ref|WP_024968868.1| 1 McnC protein [Microcystis aeruginosa]

Araçagi gi|817700114|ref|WP_046662633.1| 1 McnC protein [Microcystis aeruginosa]

Araçagi gi|817700114|ref|WP_046662633.1| 1 McnC protein [Microcystis aeruginosa]

Araçagi gi|817700114|ref|WP_046662633.1| 1 McnC protein [Microcystis aeruginosa]

Araçagi gi|817700114|ref|WP_046662633.1| 1 McnC protein [Microcystis aeruginosa]

Araçagi gi|1120050422|ref|WP_072924245.1| 1 non-ribosomal peptide synthetase [Microcystis aeruginosa]

Araçagi gi|1120050422|ref|WP_072924245.1| 1 non-ribosomal peptide synthetase [Microcystis aeruginosa]

Araçagi gi|1120050422|ref|WP_072924245.1| 1 non-ribosomal peptide synthetase [Microcystis aeruginosa]

Araçagi gi|1120050422|ref|WP_072924245.1| 1 non-ribosomal peptide synthetase [Microcystis aeruginosa]

Araçagi gi|817700114|ref|WP_046662633.1| 1 McnC protein [Microcystis aeruginosa]

Araçagi gi|817700114|ref|WP_046662633.1| 1 McnC protein [Microcystis aeruginosa]

Araçagi gi|817700114|ref|WP_046662633.1| 1 McnC protein [Microcystis aeruginosa]

Araçagi gi|817700114|ref|WP_046662633.1| 1 McnC protein [Microcystis aeruginosa]

Araçagi gi|1002985852|ref|WP_061430379.1| 1 non-ribosomal peptide synthetase [Microcystis aeruginosa]

Araçagi gi|1002985852|ref|WP_061430379.1| 1 non-ribosomal peptide synthetase [Microcystis aeruginosa]

Araçagi gi|1002985852|ref|WP_061430379.1| 1 non-ribosomal peptide synthetase [Microcystis aeruginosa]

Araçagi gi|1002985852|ref|WP_061430379.1| 1 non-ribosomal peptide synthetase [Microcystis aeruginosa]

Araçagi gi|488879470|ref|WP_002791695.1| 1 non-ribosomal peptide synthetase [Microcystis aeruginosa]

Araçagi gi|488879470|ref|WP_002791695.1| 1 non-ribosomal peptide synthetase [Microcystis aeruginosa]

Araçagi gi|488879470|ref|WP_002791695.1| 1 non-ribosomal peptide synthetase [Microcystis aeruginosa]

Araçagi gi|488879470|ref|WP_002791695.1| 1 non-ribosomal peptide synthetase [Microcystis aeruginosa]

Araçagi gi|488864797|ref|WP_002777036.1| 1 McnC protein [Microcystis aeruginosa]

Araçagi gi|488864797|ref|WP_002777036.1| 1 McnC protein [Microcystis aeruginosa]

Araçagi gi|488864797|ref|WP_002777036.1| 1 McnC protein [Microcystis aeruginosa]

Araçagi gi|488864797|ref|WP_002777036.1| 1 McnC protein [Microcystis aeruginosa]

Araçagi gi|488879468|ref|WP_002791693.1| 1 non-ribosomal peptide synthetase [Microcystis aeruginosa]

Araçagi gi|488874707|ref|WP_002786932.1| 1 McnA protein [Microcystis aeruginosa]

Araçagi gi|501225155|ref|WP_012268173.1| 1 McnA protein [Microcystis aeruginosa]

Araçagi gi|738448644|ref|WP_036399831.1| 1 McnA protein [Microcystis aeruginosa]

Araçagi gi|488864799|ref|WP_002777038.1| 1 non-ribosomal peptide synthetase [Microcystis aeruginosa]

Araçagi gi|640535888|ref|WP_024969394.1| 1 non-ribosomal peptide synthetase [Microcystis aeruginosa]

Araçagi gi|640535888|ref|WP_024969394.1| 1 non-ribosomal peptide synthetase [Microcystis aeruginosa]

Araçagi gi|653003253|ref|WP_027255439.1| 1 non-ribosomal peptide synthetase [Planktothrix agardhii]

Araçagi gi|653003253|ref|WP_027255439.1| 1 non-ribosomal peptide synthetase [Planktothrix agardhii]

Araçagi gi|652389975|ref|WP_026785823.1| 1 non-ribosomal peptide synthetase [Planktothrix rubescens]

Araçagi gi|652389975|ref|WP_026785823.1| 1 non-ribosomal peptide synthetase [Planktothrix rubescens]

Araçagi gi|652402435|ref|WP_026798231.1| 1 non-ribosomal peptide synthetase [Planktothrix prolifica]

Araçagi gi|652402435|ref|WP_026798231.1| 1 non-ribosomal peptide synthetase [Planktothrix prolifica]

Araçagi gi|652399812|ref|WP_026795614.1| 1 MULTISPECIES: non-ribosomal peptide synthetase [Planktothrix]

Araçagi gi|652399812|ref|WP_026795614.1| 1 MULTISPECIES: non-ribosomal peptide synthetase [Planktothrix]

Araçagi gi|1060047545|ref|WP_069074216.1| 1 hypothetical protein [Nostoc sp. KVJ20]

Araçagi gi|1060047545|ref|WP_069074216.1| 1 hypothetical protein [Nostoc sp. KVJ20]

Araçagi gi|1060047545|ref|WP_069074216.1| 1 hypothetical protein [Nostoc sp. KVJ20]

Araçagi gi|652389981|ref|WP_026785829.1| 1 McnC protein [Planktothrix rubescens]

Araçagi gi|652389981|ref|WP_026785829.1| 1 McnC protein [Planktothrix rubescens]

Araçagi gi|652389981|ref|WP_026785829.1| 1 McnC protein [Planktothrix rubescens]

Araçagi gi|652389981|ref|WP_026785829.1| 1 McnC protein [Planktothrix rubescens]

Araçagi gi|1105152502|ref|WP_071823928.1| 1 hypothetical protein [Microcystis aeruginosa]

Araçagi gi|515351913|ref|WP_016863946.1| 1 non-ribosomal peptide synthetase

Araçagi gi|652326501|ref|WP_026723819.1| 1 non-ribosomal peptide synthase [Fischerella sp. PCC 9431]

Araçagi gi|652326600|ref|WP_026723918.1| 1 non-ribosomal peptide synthetase [Fischerella sp. PCC 9431]

Araçagi gi|652326600|ref|WP_026723918.1| 1 non-ribosomal peptide synthetase [Fischerella sp. PCC 9431]

Araçagi gi|504892622|ref|WP_015079724.1| 1 anabaenopeptilide synthetase ApdD [Anabaena sp. 90]

Araçagi gi|1120050422|ref|WP_072924245.1| 1 non-ribosomal peptide synthetase [Microcystis aeruginosa]

Araçagi gi|1120050422|ref|WP_072924245.1| 1 non-ribosomal peptide synthetase [Microcystis aeruginosa]

Araçagi gi|1120050422|ref|WP_072924245.1| 1 non-ribosomal peptide synthetase [Microcystis aeruginosa]

Araçagi gi|1120050422|ref|WP_072924245.1| 1 non-ribosomal peptide synthetase [Microcystis aeruginosa]

Araçagi gi|488864797|ref|WP_002777036.1| 1 McnC protein [Microcystis aeruginosa]

Araçagi gi|488864797|ref|WP_002777036.1| 1 McnC protein [Microcystis aeruginosa]

Araçagi gi|488864797|ref|WP_002777036.1| 1 McnC protein [Microcystis aeruginosa]

Araçagi gi|488864797|ref|WP_002777036.1| 1 McnC protein [Microcystis aeruginosa]

Araçagi gi|1002985852|ref|WP_061430379.1| 1 non-ribosomal peptide synthetase [Microcystis aeruginosa]

Araçagi gi|1002985852|ref|WP_061430379.1| 1 non-ribosomal peptide synthetase [Microcystis aeruginosa]

Araçagi gi|1002985852|ref|WP_061430379.1| 1 non-ribosomal peptide synthetase [Microcystis aeruginosa]

Araçagi gi|1002985852|ref|WP_061430379.1| 1 non-ribosomal peptide synthetase [Microcystis aeruginosa]

Araçagi gi|640535236|ref|WP_024968868.1| 1 McnC protein [Microcystis aeruginosa]

Araçagi gi|640535236|ref|WP_024968868.1| 1 McnC protein [Microcystis aeruginosa]

Araçagi gi|640535236|ref|WP_024968868.1| 1 McnC protein [Microcystis aeruginosa]

Araçagi gi|640535236|ref|WP_024968868.1| 1 McnC protein [Microcystis aeruginosa]

Araçagi gi|817700114|ref|WP_046662633.1| 1 McnC protein [Microcystis aeruginosa]

Araçagi gi|817700114|ref|WP_046662633.1| 1 McnC protein [Microcystis aeruginosa]

Araçagi gi|817700114|ref|WP_046662633.1| 1 McnC protein [Microcystis aeruginosa]

Araçagi gi|817700114|ref|WP_046662633.1| 1 McnC protein [Microcystis aeruginosa]

Araçagi gi|763120035|ref|WP_043999949.1| 1 non-ribosomal peptide synthetase [Microcystis aeruginosa]

Araçagi gi|495474985|ref|WP_008199672.1| 1 non-ribosomal peptide synthetase [Microcystis sp. T1-4]

Araçagi gi|488849077|ref|WP_002761483.1| 1 non-ribosomal peptide synthetase [Microcystis aeruginosa]

Araçagi gi|1065016638|ref|WP_069474157.1| 1 non-ribosomal peptide synthetase [Microcystis aeruginosa]

Araçagi gi|513845141|ref|WP_016515103.1| 1 non-ribosomal peptide synthetase [Microcystis aeruginosa]

Araçagi gi|652389981|ref|WP_026785829.1| 1 McnC protein [Planktothrix rubescens]

Araçagi gi|652389981|ref|WP_026785829.1| 1 McnC protein [Planktothrix rubescens]

Araçagi gi|652389981|ref|WP_026785829.1| 1 McnC protein [Planktothrix rubescens]

Araçagi gi|652389981|ref|WP_026785829.1| 1 McnC protein [Planktothrix rubescens]

Araçagi gi|488838375|ref|WP_002750781.1| 1 non-ribosomal peptide synthetase [Microcystis aeruginosa]

Araçagi gi|488829433|ref|WP_002741839.1| 1 McnE protein [Microcystis aeruginosa]

Araçagi gi|490389196|ref|WP_004268651.1| 1 non-ribosomal peptide synthetase [Microcystis aeruginosa]

Araçagi gi|488884387|ref|WP_002796612.1| 1 non-ribosomal peptide synthetase [Microcystis aeruginosa]

Araçagi gi|504891838|ref|WP_015078940.1| 1 non-ribosomal peptide synthetase [Anabaena sp. 90]

Araçagi gi|515877976|ref|WP_017308559.1| 1 non-ribosomal peptide synthetase [Fischerella sp. PCC 9339]

Araçagi gi|504994657|ref|WP_015181759.1| 1 non-ribosomal peptide synthase [Microcoleus sp. PCC 7113]

Araçagi gi|515878079|ref|WP_017308662.1| 1 non-ribosomal peptide synthetase [Fischerella sp. PCC 9339]

Araçagi gi|515878079|ref|WP_017308662.1| 1 non-ribosomal peptide synthetase [Fischerella sp. PCC 9339]

Araçagi gi|1027887380|ref|WP_063779441.1| 1 non-ribosomal peptide synthetase [Tolypothrix bouteillei]

Araçagi gi|488838375|ref|WP_002750781.1| 1 non-ribosomal peptide synthetase [Microcystis aeruginosa]

Araçagi gi|640535235|ref|WP_024968867.1| 1 non-ribosomal peptide synthetase [Microcystis aeruginosa]

Araçagi gi|488874710|ref|WP_002786935.1| 1 non-ribosomal peptide synthetase [Microcystis aeruginosa]

Araçagi gi|488864795|ref|WP_002777034.1| 1 non-ribosomal peptide synthetase [Microcystis aeruginosa]

Araçagi gi|488829433|ref|WP_002741839.1| 1 McnE protein [Microcystis aeruginosa]

Araçagi gi|1121311258|ref|WP_073622402.1| 1 hypothetical protein [Calothrix sp. HK-06]

Araçagi gi|652389973|ref|WP_026785821.1| 1 non-ribosomal peptide synthetase [Planktothrix rubescens]

Araçagi gi|652389973|ref|WP_026785821.1| 1 non-ribosomal peptide synthetase [Planktothrix rubescens]

Araçagi gi|653003255|ref|WP_027255441.1| 1 non-ribosomal peptide synthetase [Planktothrix agardhii]

Araçagi gi|653003255|ref|WP_027255441.1| 1 non-ribosomal peptide synthetase [Planktothrix agardhii]

Araçagi gi|652402437|ref|WP_026798233.1| 1 non-ribosomal peptide synthetase [Planktothrix prolifica]

Araçagi gi|652402437|ref|WP_026798233.1| 1 non-ribosomal peptide synthetase [Planktothrix prolifica]

Araçagi gi|652996393|ref|WP_027248860.1| 1 non-ribosomal peptide synthetase [Planktothrix agardhii]

Araçagi gi|652996393|ref|WP_027248860.1| 1 non-ribosomal peptide synthetase [Planktothrix agardhii]

Araçagi gi|652399810|ref|WP_026795612.1| 1 MULTISPECIES: non-ribosomal peptide synthetase [Planktothrix]

Araçagi gi|652399810|ref|WP_026795612.1| 1 MULTISPECIES: non-ribosomal peptide synthetase [Planktothrix]

Araçagi gi|652402435|ref|WP_026798231.1| 1 non-ribosomal peptide synthetase [Planktothrix prolifica]

Araçagi gi|652402435|ref|WP_026798231.1| 1 non-ribosomal peptide synthetase [Planktothrix prolifica]

Araçagi gi|652389975|ref|WP_026785823.1| 1 non-ribosomal peptide synthetase [Planktothrix rubescens]

Araçagi gi|652389975|ref|WP_026785823.1| 1 non-ribosomal peptide synthetase [Planktothrix rubescens]

Araçagi gi|652996395|ref|WP_027248862.1| 1 non-ribosomal peptide synthetase [Planktothrix agardhii]

Araçagi gi|652996395|ref|WP_027248862.1| 1 non-ribosomal peptide synthetase [Planktothrix agardhii]

Araçagi gi|653003253|ref|WP_027255439.1| 1 non-ribosomal peptide synthetase [Planktothrix agardhii]

Araçagi gi|653003253|ref|WP_027255439.1| 1 non-ribosomal peptide synthetase [Planktothrix agardhii]

Araçagi gi|652399812|ref|WP_026795614.1| 1 MULTISPECIES: non-ribosomal peptide synthetase [Planktothrix]

Araçagi gi|652399812|ref|WP_026795614.1| 1 MULTISPECIES: non-ribosomal peptide synthetase [Planktothrix]

Araçagi gi|1119342681|ref|WP_072319556.1| 1 hypothetical protein [Microcystis aeruginosa]

Araçagi gi|652996399|ref|WP_027248866.1| 1 non-ribosomal peptide synthetase [Planktothrix agardhii]

Araçagi gi|652996399|ref|WP_027248866.1| 1 non-ribosomal peptide synthetase [Planktothrix agardhii]

Araçagi gi|918105860|ref|WP_052331149.1| 1 non-ribosomal peptide synthetase [Planktothrix agardhii]

Araçagi gi|763056259|ref|WP_043938516.1| 1 non-ribosomal peptide synthetase

Araçagi gi|1060047544|ref|WP_069074215.1| 1 non-ribosomal peptide synthetase [Nostoc sp. KVJ20]

Araçagi gi|1060047544|ref|WP_069074215.1| 1 non-ribosomal peptide synthetase [Nostoc sp. KVJ20]

Araçagi gi|754792464|ref|WP_042156042.1| 1 McnC protein [Planktothrix agardhii]

Araçagi gi|754792464|ref|WP_042156042.1| 1 McnC protein [Planktothrix agardhii]

Araçagi gi|754792464|ref|WP_042156042.1| 1 McnC protein [Planktothrix agardhii]

Araçagi gi|754792464|ref|WP_042156042.1| 1 McnC protein [Planktothrix agardhii]

Araçagi gi|1002985852|ref|WP_061430379.1| 1 non-ribosomal peptide synthetase [Microcystis aeruginosa]

Araçagi gi|1002985852|ref|WP_061430379.1| 1 non-ribosomal peptide synthetase [Microcystis aeruginosa]

Araçagi gi|1002985852|ref|WP_061430379.1| 1 non-ribosomal peptide synthetase [Microcystis aeruginosa]

Araçagi gi|1002985852|ref|WP_061430379.1| 1 non-ribosomal peptide synthetase [Microcystis aeruginosa]

Araçagi gi|817700114|ref|WP_046662633.1| 1 McnC protein [Microcystis aeruginosa]

Araçagi gi|817700114|ref|WP_046662633.1| 1 McnC protein [Microcystis aeruginosa]

Araçagi gi|817700114|ref|WP_046662633.1| 1 McnC protein [Microcystis aeruginosa]

Araçagi gi|817700114|ref|WP_046662633.1| 1 McnC protein [Microcystis aeruginosa]

Araçagi gi|488864797|ref|WP_002777036.1| 1 McnC protein [Microcystis aeruginosa]

Araçagi gi|488864797|ref|WP_002777036.1| 1 McnC protein [Microcystis aeruginosa]

Araçagi gi|488864797|ref|WP_002777036.1| 1 McnC protein [Microcystis aeruginosa]

Araçagi gi|488864797|ref|WP_002777036.1| 1 McnC protein [Microcystis aeruginosa]

Araçagi gi|501225153|ref|WP_012268171.1| 1 McnC protein [Microcystis aeruginosa]

Araçagi gi|501225153|ref|WP_012268171.1| 1 McnC protein [Microcystis aeruginosa]

Araçagi gi|501225153|ref|WP_012268171.1| 1 McnC protein [Microcystis aeruginosa]

Araçagi gi|501225153|ref|WP_012268171.1| 1 McnC protein [Microcystis aeruginosa]

Araçagi gi|763120035|ref|WP_043999949.1| 1 non-ribosomal peptide synthetase [Microcystis aeruginosa]

Araçagi gi|488849077|ref|WP_002761483.1| 1 non-ribosomal peptide synthetase [Microcystis aeruginosa]

Araçagi gi|495474985|ref|WP_008199672.1| 1 non-ribosomal peptide synthetase [Microcystis sp. T1-4]

Araçagi gi|1065016638|ref|WP_069474157.1| 1 non-ribosomal peptide synthetase [Microcystis aeruginosa]

Araçagi gi|513845141|ref|WP_016515103.1| 1 non-ribosomal peptide synthetase [Microcystis aeruginosa]

Araçagi gi|488852638|ref|WP_002765044.1| 1 hypothetical protein [Microcystis aeruginosa]

Araçagi gi|488829433|ref|WP_002741839.1| 1 McnE protein [Microcystis aeruginosa]

Araçagi gi|1120050425|ref|WP_072924248.1| 1 non-ribosomal peptide synthetase [Microcystis aeruginosa]

Araçagi gi|488864795|ref|WP_002777034.1| 1 non-ribosomal peptide synthetase [Microcystis aeruginosa]

Araçagi gi|488838375|ref|WP_002750781.1| 1 non-ribosomal peptide synthetase [Microcystis aeruginosa]

Araçagi gi|553736495|ref|WP_023070737.1| 1 amino acid adenylation domain protein [Leptolyngbya sp. Heron Island J]

Araçagi gi|493566603|ref|WP_006519935.1| 1 non-ribosomal peptide synthase domain TIGR01720 [Leptolyngbya sp. PCC 7375]

Araçagi gi|640535888|ref|WP_024969394.1| 1 non-ribosomal peptide synthetase [Microcystis aeruginosa]

Araçagi gi|488839879|ref|WP_002752285.1| 1 non-ribosomal peptide synthetase [Microcystis aeruginosa]

Araçagi gi|488893012|ref|WP_002804157.1| 1 non-ribosomal peptide synthetase [Microcystis aeruginosa]

Araçagi gi|652399812|ref|WP_026795614.1| 1 MULTISPECIES: non-ribosomal peptide synthetase [Planktothrix]

Araçagi gi|652402435|ref|WP_026798231.1| 1 non-ribosomal peptide synthetase [Planktothrix prolifica]

Araçagi gi|763057492|ref|WP_043939613.1| 1 non-ribosomal peptide synthetase [Planktothrix prolifica]

Araçagi gi|488861782|ref|WP_002774021.1| 1 MicC protein [Microcystis aeruginosa]

Araçagi gi|754792464|ref|WP_042156042.1| 1 McnC protein [Planktothrix agardhii]

Araçagi gi|754792464|ref|WP_042156042.1| 1 McnC protein [Planktothrix agardhii]

Araçagi gi|754792464|ref|WP_042156042.1| 1 McnC protein [Planktothrix agardhii]

Araçagi gi|754792464|ref|WP_042156042.1| 1 McnC protein [Planktothrix agardhii]

Araçagi gi|754792464|ref|WP_042156042.1| 1 McnC protein [Planktothrix agardhii]

Araçagi gi|640535236|ref|WP_024968868.1| 1 McnC protein [Microcystis aeruginosa]

Araçagi gi|640535236|ref|WP_024968868.1| 1 McnC protein [Microcystis aeruginosa]

Araçagi gi|640535236|ref|WP_024968868.1| 1 McnC protein [Microcystis aeruginosa]

Araçagi gi|640535236|ref|WP_024968868.1| 1 McnC protein [Microcystis aeruginosa]

Araçagi gi|640535236|ref|WP_024968868.1| 1 McnC protein [Microcystis aeruginosa]

Araçagi gi|501225153|ref|WP_012268171.1| 1 McnC protein [Microcystis aeruginosa]

Araçagi gi|501225153|ref|WP_012268171.1| 1 McnC protein [Microcystis aeruginosa]

Araçagi gi|501225153|ref|WP_012268171.1| 1 McnC protein [Microcystis aeruginosa]

Araçagi gi|501225153|ref|WP_012268171.1| 1 McnC protein [Microcystis aeruginosa]

Araçagi gi|501225153|ref|WP_012268171.1| 1 McnC protein [Microcystis aeruginosa]

Araçagi gi|817700114|ref|WP_046662633.1| 1 McnC protein [Microcystis aeruginosa]

Araçagi gi|817700114|ref|WP_046662633.1| 1 McnC protein [Microcystis aeruginosa]

Araçagi gi|817700114|ref|WP_046662633.1| 1 McnC protein [Microcystis aeruginosa]

Araçagi gi|817700114|ref|WP_046662633.1| 1 McnC protein [Microcystis aeruginosa]

Araçagi gi|817700114|ref|WP_046662633.1| 1 McnC protein [Microcystis aeruginosa]

Araçagi gi|488864797|ref|WP_002777036.1| 1 McnC protein [Microcystis aeruginosa]

Araçagi gi|488864797|ref|WP_002777036.1| 1 McnC protein [Microcystis aeruginosa]

Araçagi gi|488864797|ref|WP_002777036.1| 1 McnC protein [Microcystis aeruginosa]

Araçagi gi|488864797|ref|WP_002777036.1| 1 McnC protein [Microcystis aeruginosa]

Araçagi gi|488864797|ref|WP_002777036.1| 1 McnC protein [Microcystis aeruginosa]

Araçagi gi|754792451|ref|WP_042156030.1| 1 non-ribosomal peptide synthetase [Planktothrix agardhii]

Araçagi gi|754792451|ref|WP_042156030.1| 1 non-ribosomal peptide synthetase [Planktothrix agardhii]

Araçagi gi|652389972|ref|WP_026785820.1| 1 non-ribosomal peptide synthetase [Planktothrix rubescens]

Araçagi gi|652399809|ref|WP_026795611.1| 1 MULTISPECIES: non-ribosomal peptide synthetase [Planktothrix]

Araçagi gi|652402438|ref|WP_026798234.1| 1 non-ribosomal peptide synthetase [Planktothrix prolifica]

Araçagi gi|653003256|ref|WP_027255442.1| 1 non-ribosomal peptide synthetase [Planktothrix agardhii]

Araçagi gi|488829431|ref|WP_002741837.1| 1 McnC protein [Microcystis aeruginosa]

Araçagi gi|488829431|ref|WP_002741837.1| 1 McnC protein [Microcystis aeruginosa]

Araçagi gi|488829431|ref|WP_002741837.1| 1 McnC protein [Microcystis aeruginosa]

Araçagi gi|488829431|ref|WP_002741837.1| 1 McnC protein [Microcystis aeruginosa]

Araçagi gi|1002985852|ref|WP_061430379.1| 1 non-ribosomal peptide synthetase [Microcystis aeruginosa]

Araçagi gi|1002985852|ref|WP_061430379.1| 1 non-ribosomal peptide synthetase [Microcystis aeruginosa]

Araçagi gi|1002985852|ref|WP_061430379.1| 1 non-ribosomal peptide synthetase [Microcystis aeruginosa]

Araçagi gi|1002985852|ref|WP_061430379.1| 1 non-ribosomal peptide synthetase [Microcystis aeruginosa]

Araçagi gi|488879470|ref|WP_002791695.1| 1 non-ribosomal peptide synthetase [Microcystis aeruginosa]

Araçagi gi|488879470|ref|WP_002791695.1| 1 non-ribosomal peptide synthetase [Microcystis aeruginosa]

Araçagi gi|488879470|ref|WP_002791695.1| 1 non-ribosomal peptide synthetase [Microcystis aeruginosa]

Araçagi gi|488879470|ref|WP_002791695.1| 1 non-ribosomal peptide synthetase [Microcystis aeruginosa]

Araçagi gi|501225153|ref|WP_012268171.1| 1 McnC protein [Microcystis aeruginosa]

Araçagi gi|501225153|ref|WP_012268171.1| 1 McnC protein [Microcystis aeruginosa]

Araçagi gi|501225153|ref|WP_012268171.1| 1 McnC protein [Microcystis aeruginosa]

Araçagi gi|501225153|ref|WP_012268171.1| 1 McnC protein [Microcystis aeruginosa]

Araçagi gi|1120050422|ref|WP_072924245.1| 1 non-ribosomal peptide synthetase [Microcystis aeruginosa]

Araçagi gi|1120050422|ref|WP_072924245.1| 1 non-ribosomal peptide synthetase [Microcystis aeruginosa]

Araçagi gi|1120050422|ref|WP_072924245.1| 1 non-ribosomal peptide synthetase [Microcystis aeruginosa]

Araçagi gi|1120050422|ref|WP_072924245.1| 1 non-ribosomal peptide synthetase [Microcystis aeruginosa]

Araçagi gi|763115833|ref|WP_043995747.1| 1 McyC protein [Microcystis aeruginosa]

Araçagi gi|1120049988|ref|WP_072923811.1| 1 non-ribosomal peptide synthetase [Microcystis aeruginosa]

Araçagi gi|488849301|ref|WP_002761707.1| 1 McyC protein [Microcystis aeruginosa]

Araçagi gi|513852747|ref|WP_016517213.1| 1 non-ribosomal peptide synthetase [Microcystis aeruginosa]

Araçagi gi|501223608|ref|WP_012266626.1| 1 McyC protein [Microcystis aeruginosa]

Araçagi gi|488874710|ref|WP_002786935.1| 1 non-ribosomal peptide synthetase [Microcystis aeruginosa]

Araçagi gi|488879472|ref|WP_002791697.1| 1 non-ribosomal peptide synthetase [Microcystis aeruginosa]

Araçagi gi|488829433|ref|WP_002741839.1| 1 McnE protein [Microcystis aeruginosa]

Araçagi gi|488838375|ref|WP_002750781.1| 1 non-ribosomal peptide synthetase [Microcystis aeruginosa]

Araçagi gi|488864795|ref|WP_002777034.1| 1 non-ribosomal peptide synthetase [Microcystis aeruginosa]

Araçagi gi|515887086|ref|WP_017317669.1| 1 non-ribosomal peptide synthetase [Mastigocladopsis repens]

Araçagi gi|515887086|ref|WP_017317669.1| 1 non-ribosomal peptide synthetase [Mastigocladopsis repens]

Araçagi gi|516355639|ref|WP_017745672.1| 1 non-ribosomal peptide synthetase [Scytonema hofmannii]

Araçagi gi|516355639|ref|WP_017745672.1| 1 non-ribosomal peptide synthetase [Scytonema hofmannii]

Araçagi gi|740233659|ref|WP_038074856.1| 1 non-ribosomal peptide synthetase [Tolypothrix bouteillei]

Araçagi gi|764669229|ref|WP_044451277.1| 1 non-ribosomal peptide synthetase [Mastigocladus laminosus]

Araçagi gi|493040208|ref|WP_006106018.1| 1 non-ribosomal peptide synthetase [Coleofasciculus chthonoplastes]

Araçagi gi|1130427890|ref|WP_075596637.1| 1 hypothetical protein [Oscillatoriales cyanobacterium 'hensonii']

Araçagi gi|488829431|ref|WP_002741837.1| 1 McnC protein [Microcystis aeruginosa]

Araçagi gi|488829431|ref|WP_002741837.1| 1 McnC protein [Microcystis aeruginosa]

Araçagi gi|488829431|ref|WP_002741837.1| 1 McnC protein [Microcystis aeruginosa]

Araçagi gi|488829431|ref|WP_002741837.1| 1 McnC protein [Microcystis aeruginosa]

Araçagi gi|501225153|ref|WP_012268171.1| 1 McnC protein [Microcystis aeruginosa]

Araçagi gi|501225153|ref|WP_012268171.1| 1 McnC protein [Microcystis aeruginosa]

Araçagi gi|501225153|ref|WP_012268171.1| 1 McnC protein [Microcystis aeruginosa]

Araçagi gi|501225153|ref|WP_012268171.1| 1 McnC protein [Microcystis aeruginosa]

Araçagi gi|488864797|ref|WP_002777036.1| 1 McnC protein [Microcystis aeruginosa]

Araçagi gi|488864797|ref|WP_002777036.1| 1 McnC protein [Microcystis aeruginosa]

Araçagi gi|488864797|ref|WP_002777036.1| 1 McnC protein [Microcystis aeruginosa]

Araçagi gi|488864797|ref|WP_002777036.1| 1 McnC protein [Microcystis aeruginosa]

Araçagi gi|1120050422|ref|WP_072924245.1| 1 non-ribosomal peptide synthetase [Microcystis aeruginosa]

Araçagi gi|1120050422|ref|WP_072924245.1| 1 non-ribosomal peptide synthetase [Microcystis aeruginosa]

Araçagi gi|1120050422|ref|WP_072924245.1| 1 non-ribosomal peptide synthetase [Microcystis aeruginosa]

Araçagi gi|1120050422|ref|WP_072924245.1| 1 non-ribosomal peptide synthetase [Microcystis aeruginosa]

Araçagi gi|817700114|ref|WP_046662633.1| 1 McnC protein [Microcystis aeruginosa]

Araçagi gi|817700114|ref|WP_046662633.1| 1 McnC protein [Microcystis aeruginosa]

Araçagi gi|817700114|ref|WP_046662633.1| 1 McnC protein [Microcystis aeruginosa]

Araçagi gi|817700114|ref|WP_046662633.1| 1 McnC protein [Microcystis aeruginosa]

Araçagi gi|504959837|ref|WP_015146939.1| 1 non-ribosomal peptide synthase [Oscillatoria acuminata]

Araçagi gi|504959837|ref|WP_015146939.1| 1 non-ribosomal peptide synthase [Oscillatoria acuminata]

Araçagi gi|1121324733|ref|WP_073634530.1| 1 non-ribosomal peptide synthetase [Scytonema sp. HK-05]

Araçagi gi|488849097|ref|WP_002761503.1| 1 non-ribosomal peptide synthetase [Microcystis aeruginosa]

Araçagi gi|1011379797|ref|WP_062292562.1| 1 non-ribosomal peptide synthetase [Nostoc piscinale]

Araçagi gi|1011379797|ref|WP_062292562.1| 1 non-ribosomal peptide synthetase [Nostoc piscinale]

Araçagi gi|1011379797|ref|WP_062292562.1| 1 non-ribosomal peptide synthetase [Nostoc piscinale]

Araçagi gi|515515844|ref|WP_016949098.1| 1 hypothetical protein [Anabaena sp. PCC 7108]

Araçagi gi|1121319237|ref|WP_073629376.1| 1 non-ribosomal peptide synthetase [Scytonema sp. HK-05]

Araçagi gi|1121319237|ref|WP_073629376.1| 1 non-ribosomal peptide synthetase [Scytonema sp. HK-05]

Araçagi gi|1121319237|ref|WP_073629376.1| 1 non-ribosomal peptide synthetase [Scytonema sp. HK-05]

Araçagi gi|913451636|ref|WP_050431045.1| 1 non-ribosomal peptide synthetase [Chondromyces crocatus]

Araçagi gi|488864798|ref|WP_002777037.1| 1 non-ribosomal peptide synthetase [Microcystis aeruginosa]

Araçagi gi|488864795|ref|WP_002777034.1| 1 non-ribosomal peptide synthetase [Microcystis aeruginosa]

Araçagi gi|501225152|ref|WP_012268170.1| 1 non-ribosomal peptide synthetase [Microcystis aeruginosa]

Araçagi gi|488838375|ref|WP_002750781.1| 1 non-ribosomal peptide synthetase [Microcystis aeruginosa]

Araçagi gi|640535235|ref|WP_024968867.1| 1 non-ribosomal peptide synthetase [Microcystis aeruginosa]

Araçagi gi|495464945|ref|WP_008189636.1| 1 non-ribosomal peptide synthetase [Moorea producens]

Araçagi gi|495464945|ref|WP_008189636.1| 1 non-ribosomal peptide synthetase [Moorea producens]

Araçagi gi|1094117065|ref|WP_071106564.1| 1 non-ribosomal peptide synthetase [Moorea producens]

Araçagi gi|1094117065|ref|WP_071106564.1| 1 non-ribosomal peptide synthetase [Moorea producens]

Araçagi gi|515877283|ref|WP_017307866.1| 1 non-ribosomal peptide synthetase [Fischerella sp. PCC 9339]

Araçagi gi|1130427778|ref|WP_075596525.1| 1 non-ribosomal peptide synthetase [Oscillatoriales cyanobacterium 'hensonii']

Araçagi gi|1084329345|ref|WP_070393404.1| 1 non-ribosomal peptide synthetase [Moorea producens]

Araçagi gi|1105152502|ref|WP_071823928.1| 1 hypothetical protein [Microcystis aeruginosa]

Araçagi gi|652326501|ref|WP_026723819.1| 1 non-ribosomal peptide synthase [Fischerella sp. PCC 9431]

Araçagi gi|1027926135|ref|WP_063800724.1| 1 non-ribosomal peptide synthetase [Mastigocoleus testarum]

Araçagi gi|515861075|ref|WP_017291703.1| 1 non-ribosomal peptide synthetase [Leptolyngbya boryana]

Araçagi gi|1094117065|ref|WP_071106564.1| 1 non-ribosomal peptide synthetase [Moorea producens]

Araçagi gi|1119342681|ref|WP_072319556.1| 1 hypothetical protein [Microcystis aeruginosa]

Araçagi gi|1119342681|ref|WP_072319556.1| 1 hypothetical protein [Microcystis aeruginosa]

Araçagi gi|1119342681|ref|WP_072319556.1| 1 hypothetical protein [Microcystis aeruginosa]

Araçagi gi|918105860|ref|WP_052331149.1| 1 non-ribosomal peptide synthetase [Planktothrix agardhii]

Araçagi gi|918105860|ref|WP_052331149.1| 1 non-ribosomal peptide synthetase [Planktothrix agardhii]

Araçagi gi|763056259|ref|WP_043938516.1| 1 non-ribosomal peptide synthetase

Araçagi gi|652996399|ref|WP_027248866.1| 1 non-ribosomal peptide synthetase [Planktothrix agardhii]

Araçagi gi|652996399|ref|WP_027248866.1| 1 non-ribosomal peptide synthetase [Planktothrix agardhii]

Araçagi gi|652996399|ref|WP_027248866.1| 1 non-ribosomal peptide synthetase [Planktothrix agardhii]

Araçagi gi|501377219|ref|WP_012408785.1| 1 non-ribosomal peptide synthetase [Nostoc punctiforme]

Araçagi gi|501377219|ref|WP_012408785.1| 1 non-ribosomal peptide synthetase [Nostoc punctiforme]

Araçagi gi|501377219|ref|WP_012408785.1| 1 non-ribosomal peptide synthetase [Nostoc punctiforme]

Araçagi gi|1002987251|ref|WP_061431778.1| 1 non-ribosomal peptide synthetase [Microcystis aeruginosa]

Araçagi gi|488884831|ref|WP_002797056.1| 1 McyA protein [Microcystis aeruginosa]

Araçagi gi|501223610|ref|WP_012266628.1| 1 McyA protein [Microcystis aeruginosa]

Araçagi gi|490265066|ref|WP_004161726.1| 1 McyA protein [Microcystis aeruginosa]

Araçagi gi|1120049983|ref|WP_072923806.1| 1 non-ribosomal peptide synthetase [Microcystis aeruginosa]

Araçagi gi|516352623|ref|WP_017742656.1| 1 non-ribosomal peptide synthetase [Scytonema hofmannii]

Araçagi gi|516352623|ref|WP_017742656.1| 1 non-ribosomal peptide synthetase [Scytonema hofmannii]

Araçagi gi|516352623|ref|WP_017742656.1| 1 non-ribosomal peptide synthetase [Scytonema hofmannii]

Araçagi gi|516352623|ref|WP_017742656.1| 1 non-ribosomal peptide synthetase [Scytonema hofmannii]

Araçagi gi|494595789|ref|WP_007354048.1| 1 MULTISPECIES: non-ribosomal peptide synthetase [Kamptonema]

Araçagi gi|516352640|ref|WP_017742673.1| 1 non-ribosomal peptide synthetase [Scytonema hofmannii]

Araçagi gi|516352640|ref|WP_017742673.1| 1 non-ribosomal peptide synthetase [Scytonema hofmannii]

Araçagi gi|516352640|ref|WP_017742673.1| 1 non-ribosomal peptide synthetase [Scytonema hofmannii]

Araçagi gi|516352640|ref|WP_017742673.1| 1 non-ribosomal peptide synthetase [Scytonema hofmannii]

Araçagi gi|515878078|ref|WP_017308661.1| 1 non-ribosomal peptide synthetase [Fischerella sp. PCC 9339]

Araçagi gi|515878078|ref|WP_017308661.1| 1 non-ribosomal peptide synthetase [Fischerella sp. PCC 9339]

Araçagi gi|515878078|ref|WP_017308661.1| 1 non-ribosomal peptide synthetase [Fischerella sp. PCC 9339]

Araçagi gi|501377220|ref|WP_012408786.1| 1 non-ribosomal peptide synthetase [Nostoc punctiforme]

Araçagi gi|501377220|ref|WP_012408786.1| 1 non-ribosomal peptide synthetase [Nostoc punctiforme]

Araçagi gi|1054850717|ref|WP_066612889.1| 1 non-ribosomal peptide synthetase [Scytonema hofmannii]

Araçagi gi|1054850717|ref|WP_066612889.1| 1 non-ribosomal peptide synthetase [Scytonema hofmannii]

Araçagi gi|1054850717|ref|WP_066612889.1| 1 non-ribosomal peptide synthetase [Scytonema hofmannii]

Araçagi gi|501377217|ref|WP_012408783.1| 1 non-ribosomal peptide synthetase [Nostoc punctiforme]

Araçagi gi|501377217|ref|WP_012408783.1| 1 non-ribosomal peptide synthetase [Nostoc punctiforme]

Araçagi gi|501377217|ref|WP_012408783.1| 1 non-ribosomal peptide synthetase [Nostoc punctiforme]

Araçagi gi|501377217|ref|WP_012408783.1| 1 non-ribosomal peptide synthetase [Nostoc punctiforme]

Araçagi gi|488821608|ref|WP_002734014.1| 1 non-ribosomal peptide synthetase [Microcystis aeruginosa]

Araçagi gi|1065016475|ref|WP_069474085.1| 1 non-ribosomal peptide synthetase [Microcystis aeruginosa]

Araçagi gi|488876300|ref|WP_002788525.1| 1 non-ribosomal peptide synthetase [Microcystis aeruginosa]

Araçagi gi|1054850722|ref|WP_066612894.1| 1 hypothetical protein [Scytonema hofmannii]

Araçagi gi|1054850722|ref|WP_066612894.1| 1 hypothetical protein [Scytonema hofmannii]

Araçagi gi|1054850722|ref|WP_066612894.1| 1 hypothetical protein [Scytonema hofmannii]

Araçagi gi|516352640|ref|WP_017742673.1| 1 non-ribosomal peptide synthetase [Scytonema hofmannii]

Araçagi gi|516352640|ref|WP_017742673.1| 1 non-ribosomal peptide synthetase [Scytonema hofmannii]

Araçagi gi|516352640|ref|WP_017742673.1| 1 non-ribosomal peptide synthetase [Scytonema hofmannii]

Araçagi gi|516352640|ref|WP_017742673.1| 1 non-ribosomal peptide synthetase [Scytonema hofmannii]

Araçagi gi|1002985852|ref|WP_061430379.1| 1 non-ribosomal peptide synthetase [Microcystis aeruginosa]

Araçagi gi|1002985852|ref|WP_061430379.1| 1 non-ribosomal peptide synthetase [Microcystis aeruginosa]

Araçagi gi|1002985852|ref|WP_061430379.1| 1 non-ribosomal peptide synthetase [Microcystis aeruginosa]

Araçagi gi|1002985852|ref|WP_061430379.1| 1 non-ribosomal peptide synthetase [Microcystis aeruginosa]

Araçagi gi|501225153|ref|WP_012268171.1| 1 McnC protein [Microcystis aeruginosa]

Araçagi gi|501225153|ref|WP_012268171.1| 1 McnC protein [Microcystis aeruginosa]

Araçagi gi|501225153|ref|WP_012268171.1| 1 McnC protein [Microcystis aeruginosa]

Araçagi gi|501225153|ref|WP_012268171.1| 1 McnC protein [Microcystis aeruginosa]

Araçagi gi|817700114|ref|WP_046662633.1| 1 McnC protein [Microcystis aeruginosa]

Araçagi gi|817700114|ref|WP_046662633.1| 1 McnC protein [Microcystis aeruginosa]

Araçagi gi|817700114|ref|WP_046662633.1| 1 McnC protein [Microcystis aeruginosa]

Araçagi gi|817700114|ref|WP_046662633.1| 1 McnC protein [Microcystis aeruginosa]

Araçagi gi|640535236|ref|WP_024968868.1| 1 McnC protein [Microcystis aeruginosa]

Araçagi gi|640535236|ref|WP_024968868.1| 1 McnC protein [Microcystis aeruginosa]

Araçagi gi|640535236|ref|WP_024968868.1| 1 McnC protein [Microcystis aeruginosa]

Araçagi gi|640535236|ref|WP_024968868.1| 1 McnC protein [Microcystis aeruginosa]

Araçagi gi|1120050422|ref|WP_072924245.1| 1 non-ribosomal peptide synthetase [Microcystis aeruginosa]

Araçagi gi|1120050422|ref|WP_072924245.1| 1 non-ribosomal peptide synthetase [Microcystis aeruginosa]

Araçagi gi|1120050422|ref|WP_072924245.1| 1 non-ribosomal peptide synthetase [Microcystis aeruginosa]

Araçagi gi|1120050422|ref|WP_072924245.1| 1 non-ribosomal peptide synthetase [Microcystis aeruginosa]

Araçagi gi|817700114|ref|WP_046662633.1| 1 McnC protein [Microcystis aeruginosa]

Araçagi gi|817700114|ref|WP_046662633.1| 1 McnC protein [Microcystis aeruginosa]

Araçagi gi|817700114|ref|WP_046662633.1| 1 McnC protein [Microcystis aeruginosa]

Araçagi gi|817700114|ref|WP_046662633.1| 1 McnC protein [Microcystis aeruginosa]

Araçagi gi|652997906|ref|WP_027250316.1| 1 McnC protein [Planktothrix agardhii]

Araçagi gi|652997906|ref|WP_027250316.1| 1 McnC protein [Planktothrix agardhii]

Araçagi gi|652997906|ref|WP_027250316.1| 1 McnC protein [Planktothrix agardhii]

Araçagi gi|652997906|ref|WP_027250316.1| 1 McnC protein [Planktothrix agardhii]

Araçagi gi|652402732|ref|WP_026798523.1| 1 McnC protein [Planktothrix prolifica]

Araçagi gi|652402732|ref|WP_026798523.1| 1 McnC protein [Planktothrix prolifica]

Araçagi gi|652402732|ref|WP_026798523.1| 1 McnC protein [Planktothrix prolifica]

Araçagi gi|652402732|ref|WP_026798523.1| 1 McnC protein [Planktothrix prolifica]

Araçagi gi|1120050422|ref|WP_072924245.1| 1 non-ribosomal peptide synthetase [Microcystis aeruginosa]

Araçagi gi|1120050422|ref|WP_072924245.1| 1 non-ribosomal peptide synthetase [Microcystis aeruginosa]

Araçagi gi|1120050422|ref|WP_072924245.1| 1 non-ribosomal peptide synthetase [Microcystis aeruginosa]

Araçagi gi|1120050422|ref|WP_072924245.1| 1 non-ribosomal peptide synthetase [Microcystis aeruginosa]

Araçagi gi|1002985852|ref|WP_061430379.1| 1 non-ribosomal peptide synthetase [Microcystis aeruginosa]

Araçagi gi|1002985852|ref|WP_061430379.1| 1 non-ribosomal peptide synthetase [Microcystis aeruginosa]

Araçagi gi|1002985852|ref|WP_061430379.1| 1 non-ribosomal peptide synthetase [Microcystis aeruginosa]

Araçagi gi|1002985852|ref|WP_061430379.1| 1 non-ribosomal peptide synthetase [Microcystis aeruginosa]

Araçagi gi|640537447|ref|WP_024970455.1| 1 McnB protein

Araçagi gi|763118395|ref|WP_043998309.1| 1 McnB protein

Araçagi gi|488874708|ref|WP_002786933.1| 1 non-ribosomal peptide synthetase [Microcystis aeruginosa]

Araçagi gi|1119342681|ref|WP_072319556.1| 1 hypothetical protein [Microcystis aeruginosa]

Araçagi gi|1119342681|ref|WP_072319556.1| 1 hypothetical protein [Microcystis aeruginosa]

Araçagi gi|488864798|ref|WP_002777037.1| 1 non-ribosomal peptide synthetase [Microcystis aeruginosa]

Araçagi gi|1120050422|ref|WP_072924245.1| 1 non-ribosomal peptide synthetase [Microcystis aeruginosa]

Araçagi gi|1120050422|ref|WP_072924245.1| 1 non-ribosomal peptide synthetase [Microcystis aeruginosa]

Araçagi gi|1120050422|ref|WP_072924245.1| 1 non-ribosomal peptide synthetase [Microcystis aeruginosa]

Araçagi gi|1120050422|ref|WP_072924245.1| 1 non-ribosomal peptide synthetase [Microcystis aeruginosa]

Araçagi gi|488864797|ref|WP_002777036.1| 1 McnC protein [Microcystis aeruginosa]

Araçagi gi|488864797|ref|WP_002777036.1| 1 McnC protein [Microcystis aeruginosa]

Araçagi gi|488864797|ref|WP_002777036.1| 1 McnC protein [Microcystis aeruginosa]

Araçagi gi|488864797|ref|WP_002777036.1| 1 McnC protein [Microcystis aeruginosa]

Araçagi gi|501225153|ref|WP_012268171.1| 1 McnC protein [Microcystis aeruginosa]

Araçagi gi|501225153|ref|WP_012268171.1| 1 McnC protein [Microcystis aeruginosa]

Araçagi gi|501225153|ref|WP_012268171.1| 1 McnC protein [Microcystis aeruginosa]

Araçagi gi|501225153|ref|WP_012268171.1| 1 McnC protein [Microcystis aeruginosa]

Araçagi gi|640535236|ref|WP_024968868.1| 1 McnC protein [Microcystis aeruginosa]

Araçagi gi|640535236|ref|WP_024968868.1| 1 McnC protein [Microcystis aeruginosa]

Araçagi gi|640535236|ref|WP_024968868.1| 1 McnC protein [Microcystis aeruginosa]

Araçagi gi|640535236|ref|WP_024968868.1| 1 McnC protein [Microcystis aeruginosa]

Araçagi gi|488829431|ref|WP_002741837.1| 1 McnC protein [Microcystis aeruginosa]

Araçagi gi|488829431|ref|WP_002741837.1| 1 McnC protein [Microcystis aeruginosa]

Araçagi gi|488829431|ref|WP_002741837.1| 1 McnC protein [Microcystis aeruginosa]

Araçagi gi|488829431|ref|WP_002741837.1| 1 McnC protein [Microcystis aeruginosa]

Araçagi gi|652389975|ref|WP_026785823.1| 1 non-ribosomal peptide synthetase [Planktothrix rubescens]

Araçagi gi|652389975|ref|WP_026785823.1| 1 non-ribosomal peptide synthetase [Planktothrix rubescens]

Araçagi gi|652402435|ref|WP_026798231.1| 1 non-ribosomal peptide synthetase [Planktothrix prolifica]

Araçagi gi|652402435|ref|WP_026798231.1| 1 non-ribosomal peptide synthetase [Planktothrix prolifica]

Araçagi gi|652996395|ref|WP_027248862.1| 1 non-ribosomal peptide synthetase [Planktothrix agardhii]

Araçagi gi|652996395|ref|WP_027248862.1| 1 non-ribosomal peptide synthetase [Planktothrix agardhii]

Araçagi gi|652399812|ref|WP_026795614.1| 1 MULTISPECIES: non-ribosomal peptide synthetase [Planktothrix]

Araçagi gi|652399812|ref|WP_026795614.1| 1 MULTISPECIES: non-ribosomal peptide synthetase [Planktothrix]

Araçagi gi|653003253|ref|WP_027255439.1| 1 non-ribosomal peptide synthetase [Planktothrix agardhii]

Araçagi gi|653003253|ref|WP_027255439.1| 1 non-ribosomal peptide synthetase [Planktothrix agardhii]

Araçagi gi|488829431|ref|WP_002741837.1| 1 McnC protein [Microcystis aeruginosa]

Araçagi gi|488829431|ref|WP_002741837.1| 1 McnC protein [Microcystis aeruginosa]

Araçagi gi|488864797|ref|WP_002777036.1| 1 McnC protein [Microcystis aeruginosa]

Araçagi gi|488864797|ref|WP_002777036.1| 1 McnC protein [Microcystis aeruginosa]

Araçagi gi|1120050422|ref|WP_072924245.1| 1 non-ribosomal peptide synthetase [Microcystis aeruginosa]

Araçagi gi|488879470|ref|WP_002791695.1| 1 non-ribosomal peptide synthetase [Microcystis aeruginosa]

Araçagi gi|488879470|ref|WP_002791695.1| 1 non-ribosomal peptide synthetase [Microcystis aeruginosa]

Araçagi gi|1002985852|ref|WP_061430379.1| 1 non-ribosomal peptide synthetase [Microcystis aeruginosa]

Araçagi gi|1002985852|ref|WP_061430379.1| 1 non-ribosomal peptide synthetase [Microcystis aeruginosa]

Araçagi gi|516357525|ref|WP_017747558.1| 1 non-ribosomal peptide synthetase [Scytonema hofmannii]

Araçagi gi|505019970|ref|WP_015207072.1| 1 non-ribosomal peptide synthase [Cylindrospermum stagnale]

Araçagi gi|1002985852|ref|WP_061430379.1| 1 non-ribosomal peptide synthetase [Microcystis aeruginosa]

Araçagi gi|1002985852|ref|WP_061430379.1| 1 non-ribosomal peptide synthetase [Microcystis aeruginosa]

Araçagi gi|1002985852|ref|WP_061430379.1| 1 non-ribosomal peptide synthetase [Microcystis aeruginosa]

Araçagi gi|1002985852|ref|WP_061430379.1| 1 non-ribosomal peptide synthetase [Microcystis aeruginosa]

Araçagi gi|1120050422|ref|WP_072924245.1| 1 non-ribosomal peptide synthetase [Microcystis aeruginosa]

Araçagi gi|1120050422|ref|WP_072924245.1| 1 non-ribosomal peptide synthetase [Microcystis aeruginosa]

Araçagi gi|1120050422|ref|WP_072924245.1| 1 non-ribosomal peptide synthetase [Microcystis aeruginosa]

Araçagi gi|1120050422|ref|WP_072924245.1| 1 non-ribosomal peptide synthetase [Microcystis aeruginosa]

Araçagi gi|488879470|ref|WP_002791695.1| 1 non-ribosomal peptide synthetase [Microcystis aeruginosa]

Araçagi gi|488879470|ref|WP_002791695.1| 1 non-ribosomal peptide synthetase [Microcystis aeruginosa]

Araçagi gi|488879470|ref|WP_002791695.1| 1 non-ribosomal peptide synthetase [Microcystis aeruginosa]

Araçagi gi|488879470|ref|WP_002791695.1| 1 non-ribosomal peptide synthetase [Microcystis aeruginosa]

Araçagi gi|488829431|ref|WP_002741837.1| 1 McnC protein [Microcystis aeruginosa]

Araçagi gi|488829431|ref|WP_002741837.1| 1 McnC protein [Microcystis aeruginosa]

Araçagi gi|488829431|ref|WP_002741837.1| 1 McnC protein [Microcystis aeruginosa]

Araçagi gi|488829431|ref|WP_002741837.1| 1 McnC protein [Microcystis aeruginosa]

Araçagi gi|501225153|ref|WP_012268171.1| 1 McnC protein [Microcystis aeruginosa]

Araçagi gi|501225153|ref|WP_012268171.1| 1 McnC protein [Microcystis aeruginosa]

Araçagi gi|501225153|ref|WP_012268171.1| 1 McnC protein [Microcystis aeruginosa]

Araçagi gi|501225153|ref|WP_012268171.1| 1 McnC protein [Microcystis aeruginosa]

Araçagi gi|1120051153|ref|WP_072924976.1| 1 non-ribosomal peptide synthetase [Microcystis aeruginosa]

Araçagi gi|763155072|ref|WP_044034730.1| 1 non-ribosomal peptide synthetase

Araçagi gi|501224905|ref|WP_012267923.1| 1 non-ribosomal peptide synthetase [Microcystis aeruginosa]

Araçagi gi|488828768|ref|WP_002741174.1| 1 non-ribosomal peptide synthetase [Microcystis aeruginosa]

Araçagi gi|488876300|ref|WP_002788525.1| 1 non-ribosomal peptide synthetase [Microcystis aeruginosa]

Araçagi gi|917763913|ref|WP_052277918.1| 1 McyA protein [Microcystis panniformis]

Araçagi gi|917763913|ref|WP_052277918.1| 1 McyA protein [Microcystis panniformis]

Araçagi gi|488830429|ref|WP_002742835.1| 1 non-ribosomal peptide synthase [Microcystis aeruginosa]

Araçagi gi|488830429|ref|WP_002742835.1| 1 non-ribosomal peptide synthase [Microcystis aeruginosa]

Araçagi gi|490265066|ref|WP_004161726.1| 1 McyA protein [Microcystis aeruginosa]

Araçagi gi|490265066|ref|WP_004161726.1| 1 McyA protein [Microcystis aeruginosa]

Araçagi gi|1120049983|ref|WP_072923806.1| 1 non-ribosomal peptide synthetase [Microcystis aeruginosa]

Araçagi gi|1120049983|ref|WP_072923806.1| 1 non-ribosomal peptide synthetase [Microcystis aeruginosa]

Araçagi gi|763117447|ref|WP_043997361.1| 1 McyA protein [Microcystis aeruginosa]

Araçagi gi|763117447|ref|WP_043997361.1| 1 McyA protein [Microcystis aeruginosa]

Araçagi gi|1105152502|ref|WP_071823928.1| 1 hypothetical protein [Microcystis aeruginosa]

Araçagi gi|652326501|ref|WP_026723819.1| 1 non-ribosomal peptide synthase [Fischerella sp. PCC 9431]

Araçagi gi|515351913|ref|WP_016863946.1| 1 non-ribosomal peptide synthetase

Araçagi gi|889955689|ref|WP_048866322.1| 1 non-ribosomal peptide synthetase [Scytonema tolypothrichoides]

Araçagi gi|1121319255|ref|WP_073629394.1| 1 non-ribosomal peptide synthetase [Scytonema sp. HK-05]

Araçagi gi|652389972|ref|WP_026785820.1| 1 non-ribosomal peptide synthetase [Planktothrix rubescens]

Araçagi gi|653003256|ref|WP_027255442.1| 1 non-ribosomal peptide synthetase [Planktothrix agardhii]

Araçagi gi|754792450|ref|WP_042156029.1| 1 non-ribosomal peptide synthetase [Planktothrix agardhii]

Araçagi gi|652402438|ref|WP_026798234.1| 1 non-ribosomal peptide synthetase [Planktothrix prolifica]

Araçagi gi|652399809|ref|WP_026795611.1| 1 MULTISPECIES: non-ribosomal peptide synthetase [Planktothrix]

Araçagi gi|652996394|ref|WP_027248861.1| 1 non-ribosomal peptide synthetase [Planktothrix agardhii]

Araçagi gi|754792453|ref|WP_042156032.1| 1 non-ribosomal peptide synthetase [Planktothrix agardhii]

Araçagi gi|653003254|ref|WP_027255440.1| 1 non-ribosomal peptide synthetase [Planktothrix agardhii]

Araçagi gi|652402436|ref|WP_026798232.1| 1 non-ribosomal peptide synthetase [Planktothrix prolifica]

Araçagi gi|652389974|ref|WP_026785822.1| 1 non-ribosomal peptide synthetase [Planktothrix rubescens]

Araçagi gi|490265524|ref|WP_004162176.1| 1 non-ribosomal peptide synthetase [Microcystis aeruginosa]

Araçagi gi|1105146240|ref|WP_071823439.1| 1 hypothetical protein [Microcystis aeruginosa]

Araçagi gi|1112924879|ref|WP_071995597.1| 1 hypothetical protein [Microcystis aeruginosa]

Araçagi gi|488880990|ref|WP_002793215.1| 1 Microcystin synthetase A [Microcystis aeruginosa]

Araçagi gi|917763913|ref|WP_052277918.1| 1 McyA protein [Microcystis panniformis]

Araçagi gi|1120049983|ref|WP_072923806.1| 1 non-ribosomal peptide synthetase [Microcystis aeruginosa]

Araçagi gi|488830429|ref|WP_002742835.1| 1 non-ribosomal peptide synthase [Microcystis aeruginosa]

Araçagi gi|490265066|ref|WP_004161726.1| 1 McyA protein [Microcystis aeruginosa]

Araçagi gi|488828768|ref|WP_002741174.1| 1 non-ribosomal peptide synthetase [Microcystis aeruginosa]

Araçagi gi|488876300|ref|WP_002788525.1| 1 non-ribosomal peptide synthetase [Microcystis aeruginosa]

Araçagi gi|1120051153|ref|WP_072924976.1| 1 non-ribosomal peptide synthetase [Microcystis aeruginosa]

Araçagi gi|1002987895|ref|WP_061432422.1| 1 non-ribosomal peptide synthetase [Microcystis aeruginosa]

Araçagi gi|488881832|ref|WP_002794057.1| 1 non-ribosomal peptide synthetase [Microcystis aeruginosa]

Araçagi gi|490265530|ref|WP_004162182.1| 1 non-ribosomal peptide synthetase [Microcystis aeruginosa]

Araçagi gi|916599645|ref|WP_051206736.1| 1 hypothetical protein [Fischerella sp. PCC 9431]

Araçagi gi|654347244|ref|WP_027840649.1| 1 hypothetical protein [Mastigocoleus testarum]

Araçagi gi|738448646|ref|WP_036399833.1| 1 McnB protein [Microcystis aeruginosa]

Araçagi gi|488874708|ref|WP_002786933.1| 1 non-ribosomal peptide synthetase [Microcystis aeruginosa]

Araçagi gi|640537447|ref|WP_024970455.1| 1 McnB protein

Araçagi gi|763118395|ref|WP_043998309.1| 1 McnB protein

Araçagi gi|763154312|ref|WP_044033970.1| 1 McnB protein [Microcystis aeruginosa]

Araçagi gi|488828768|ref|WP_002741174.1| 1 non-ribosomal peptide synthetase [Microcystis aeruginosa]

Araçagi gi|488876300|ref|WP_002788525.1| 1 non-ribosomal peptide synthetase [Microcystis aeruginosa]

Araçagi gi|1120051153|ref|WP_072924976.1| 1 non-ribosomal peptide synthetase [Microcystis aeruginosa]

Araçagi gi|488849674|ref|WP_002762080.1| 1 non-ribosomal peptide synthetase [Microcystis aeruginosa]

Araçagi gi|488821608|ref|WP_002734014.1| 1 non-ribosomal peptide synthetase [Microcystis aeruginosa]

Araçagi gi|1121319241|ref|WP_073629380.1| 1 hypothetical protein [Scytonema sp. HK-05]

Araçagi gi|763118395|ref|WP_043998309.1| 1 McnB protein

Araçagi gi|1054850717|ref|WP_066612889.1| 1 non-ribosomal peptide synthetase [Scytonema hofmannii]

Araçagi gi|1054850717|ref|WP_066612889.1| 1 non-ribosomal peptide synthetase [Scytonema hofmannii]

Araçagi gi|738448646|ref|WP_036399833.1| 1 McnB protein [Microcystis aeruginosa]

Araçagi gi|488864798|ref|WP_002777037.1| 1 non-ribosomal peptide synthetase [Microcystis aeruginosa]

Araçagi gi|488829431|ref|WP_002741837.1| 1 McnC protein [Microcystis aeruginosa]

Araçagi gi|488829431|ref|WP_002741837.1| 1 McnC protein [Microcystis aeruginosa]

Araçagi gi|488829431|ref|WP_002741837.1| 1 McnC protein [Microcystis aeruginosa]

Araçagi gi|488829431|ref|WP_002741837.1| 1 McnC protein [Microcystis aeruginosa]

Araçagi gi|488864797|ref|WP_002777036.1| 1 McnC protein [Microcystis aeruginosa]

Araçagi gi|488864797|ref|WP_002777036.1| 1 McnC protein [Microcystis aeruginosa]

Araçagi gi|488864797|ref|WP_002777036.1| 1 McnC protein [Microcystis aeruginosa]

Araçagi gi|488864797|ref|WP_002777036.1| 1 McnC protein [Microcystis aeruginosa]

Araçagi gi|1120050422|ref|WP_072924245.1| 1 non-ribosomal peptide synthetase [Microcystis aeruginosa]

Araçagi gi|1120050422|ref|WP_072924245.1| 1 non-ribosomal peptide synthetase [Microcystis aeruginosa]

Araçagi gi|1120050422|ref|WP_072924245.1| 1 non-ribosomal peptide synthetase [Microcystis aeruginosa]

Araçagi gi|1120050422|ref|WP_072924245.1| 1 non-ribosomal peptide synthetase [Microcystis aeruginosa]

Araçagi gi|817700114|ref|WP_046662633.1| 1 McnC protein [Microcystis aeruginosa]

Araçagi gi|817700114|ref|WP_046662633.1| 1 McnC protein [Microcystis aeruginosa]

Araçagi gi|817700114|ref|WP_046662633.1| 1 McnC protein [Microcystis aeruginosa]

Araçagi gi|817700114|ref|WP_046662633.1| 1 McnC protein [Microcystis aeruginosa]

Araçagi gi|1002985852|ref|WP_061430379.1| 1 non-ribosomal peptide synthetase [Microcystis aeruginosa]

Araçagi gi|1002985852|ref|WP_061430379.1| 1 non-ribosomal peptide synthetase [Microcystis aeruginosa]

Araçagi gi|1002985852|ref|WP_061430379.1| 1 non-ribosomal peptide synthetase [Microcystis aeruginosa]

Araçagi gi|1002985852|ref|WP_061430379.1| 1 non-ribosomal peptide synthetase [Microcystis aeruginosa]

Araçagi gi|501377949|ref|WP_012409515.1| 1 non-ribosomal peptide synthetase [Nostoc punctiforme]

Araçagi gi|501377949|ref|WP_012409515.1| 1 non-ribosomal peptide synthetase [Nostoc punctiforme]

Araçagi gi|501377949|ref|WP_012409515.1| 1 non-ribosomal peptide synthetase [Nostoc punctiforme]

Araçagi gi|501377946|ref|WP_012409512.1| 1 non-ribosomal peptide synthetase [Nostoc punctiforme]

Araçagi gi|501377946|ref|WP_012409512.1| 1 non-ribosomal peptide synthetase [Nostoc punctiforme]

Araçagi gi|516352615|ref|WP_017742648.1| 1 non-ribosomal peptide synthetase [Scytonema hofmannii]

Araçagi gi|1121311260|ref|WP_073622404.1| 1 hypothetical protein [Calothrix sp. HK-06]

Araçagi gi|652399816|ref|WP_026795618.1| 1 MULTISPECIES: McnB protein [Planktothrix]

Araçagi gi|738448357|ref|WP_036399545.1| 1 non-ribosomal peptide synthetase [Microcystis aeruginosa]

Araçagi gi|817700114|ref|WP_046662633.1| 1 McnC protein [Microcystis aeruginosa]

Araçagi gi|817700114|ref|WP_046662633.1| 1 McnC protein [Microcystis aeruginosa]

Araçagi gi|817700114|ref|WP_046662633.1| 1 McnC protein [Microcystis aeruginosa]

Araçagi gi|817700114|ref|WP_046662633.1| 1 McnC protein [Microcystis aeruginosa]

Araçagi gi|488879470|ref|WP_002791695.1| 1 non-ribosomal peptide synthetase [Microcystis aeruginosa]

Araçagi gi|488879470|ref|WP_002791695.1| 1 non-ribosomal peptide synthetase [Microcystis aeruginosa]

Araçagi gi|488879470|ref|WP_002791695.1| 1 non-ribosomal peptide synthetase [Microcystis aeruginosa]

Araçagi gi|488879470|ref|WP_002791695.1| 1 non-ribosomal peptide synthetase [Microcystis aeruginosa]

Araçagi gi|640535236|ref|WP_024968868.1| 1 McnC protein [Microcystis aeruginosa]

Araçagi gi|640535236|ref|WP_024968868.1| 1 McnC protein [Microcystis aeruginosa]

Araçagi gi|640535236|ref|WP_024968868.1| 1 McnC protein [Microcystis aeruginosa]

Araçagi gi|640535236|ref|WP_024968868.1| 1 McnC protein [Microcystis aeruginosa]

Araçagi gi|488829431|ref|WP_002741837.1| 1 McnC protein [Microcystis aeruginosa]

Araçagi gi|488829431|ref|WP_002741837.1| 1 McnC protein [Microcystis aeruginosa]

Araçagi gi|488829431|ref|WP_002741837.1| 1 McnC protein [Microcystis aeruginosa]

Araçagi gi|488829431|ref|WP_002741837.1| 1 McnC protein [Microcystis aeruginosa]

Araçagi gi|640535887|ref|WP_024969393.1| 1 non-ribosomal peptide synthetase [Microcystis aeruginosa]

Araçagi gi|488839880|ref|WP_002752286.1| 1 non-ribosomal peptide synthetase [Microcystis aeruginosa]

Araçagi gi|488893013|ref|WP_002804158.1| 1 non-ribosomal peptide synthetase [Microcystis aeruginosa]

Araçagi gi|652399811|ref|WP_026795613.1| 1 MULTISPECIES: non-ribosomal peptide synthetase [Planktothrix]

Araçagi gi|754792453|ref|WP_042156032.1| 1 non-ribosomal peptide synthetase [Planktothrix agardhii]

Araçagi gi|738448646|ref|WP_036399833.1| 1 McnB protein [Microcystis aeruginosa]

Araçagi gi|488864798|ref|WP_002777037.1| 1 non-ribosomal peptide synthetase [Microcystis aeruginosa]

Araçagi gi|763154312|ref|WP_044033970.1| 1 McnB protein [Microcystis aeruginosa]

Araçagi gi|640537447|ref|WP_024970455.1| 1 McnB protein

Araçagi gi|763118395|ref|WP_043998309.1| 1 McnB protein

Araçagi gi|910241529|ref|WP_050046520.1| 1 non-ribosomal peptide synthetase [Tolypothrix bouteillei]

Araçagi gi|910241529|ref|WP_050046520.1| 1 non-ribosomal peptide synthetase [Tolypothrix bouteillei]

Araçagi gi|497316517|ref|WP_009630730.1| 1 non-ribosomal peptide synthetase [Synechocystis sp. PCC 7509]

Araçagi gi|521968397|ref|WP_020479985.1| 1 non-ribosomal peptide synthetase [Mastigocladopsis repens]

Araçagi gi|521968397|ref|WP_020479985.1| 1 non-ribosomal peptide synthetase [Mastigocladopsis repens]

Araçagi gi|1056313841|ref|WP_067768735.1| 1 non-ribosomal peptide synthetase [Nostoc sp. NIES-3756]

Araçagi gi|504962340|ref|WP_015149442.1| 1 non-ribosomal peptide synthase [Oscillatoria acuminata]

Araçagi gi|488821608|ref|WP_002734014.1| 1 non-ribosomal peptide synthetase [Microcystis aeruginosa]

Araçagi gi|488876300|ref|WP_002788525.1| 1 non-ribosomal peptide synthetase [Microcystis aeruginosa]

Araçagi gi|1065016475|ref|WP_069474085.1| 1 non-ribosomal peptide synthetase [Microcystis aeruginosa]

Araçagi gi|754792450|ref|WP_042156029.1| 1 non-ribosomal peptide synthetase [Planktothrix agardhii]

Araçagi gi|503099778|ref|WP_013334575.1| 1 non-ribosomal peptide synthetase [Cyanothece sp. PCC 7822]

Araçagi gi|817698756|ref|WP_046661401.1| 1 non-ribosomal peptide synthetase [Microcystis aeruginosa]

Araçagi gi|640536675|ref|WP_024969848.1| 1 non-ribosomal peptide synthetase [Microcystis aeruginosa]

Araçagi gi|1002987895|ref|WP_061432422.1| 1 non-ribosomal peptide synthetase [Microcystis aeruginosa]

Araçagi gi|488821608|ref|WP_002734014.1| 1 non-ribosomal peptide synthetase [Microcystis aeruginosa]

Araçagi gi|917761360|ref|WP_052275365.1| 1 non-ribosomal peptide synthetase [Microcystis panniformis]

Araçagi gi|640535236|ref|WP_024968868.1| 1 McnC protein [Microcystis aeruginosa]

Araçagi gi|640535236|ref|WP_024968868.1| 1 McnC protein [Microcystis aeruginosa]

Araçagi gi|640535236|ref|WP_024968868.1| 1 McnC protein [Microcystis aeruginosa]

Araçagi gi|640535236|ref|WP_024968868.1| 1 McnC protein [Microcystis aeruginosa]

Araçagi gi|488829431|ref|WP_002741837.1| 1 McnC protein [Microcystis aeruginosa]

Araçagi gi|488829431|ref|WP_002741837.1| 1 McnC protein [Microcystis aeruginosa]

Araçagi gi|488829431|ref|WP_002741837.1| 1 McnC protein [Microcystis aeruginosa]

Araçagi gi|488829431|ref|WP_002741837.1| 1 McnC protein [Microcystis aeruginosa]

Araçagi gi|488879470|ref|WP_002791695.1| 1 non-ribosomal peptide synthetase [Microcystis aeruginosa]

Araçagi gi|488879470|ref|WP_002791695.1| 1 non-ribosomal peptide synthetase [Microcystis aeruginosa]

Araçagi gi|488879470|ref|WP_002791695.1| 1 non-ribosomal peptide synthetase [Microcystis aeruginosa]

Araçagi gi|488879470|ref|WP_002791695.1| 1 non-ribosomal peptide synthetase [Microcystis aeruginosa]

Araçagi gi|501225153|ref|WP_012268171.1| 1 McnC protein [Microcystis aeruginosa]

Araçagi gi|501225153|ref|WP_012268171.1| 1 McnC protein [Microcystis aeruginosa]

Araçagi gi|501225153|ref|WP_012268171.1| 1 McnC protein [Microcystis aeruginosa]

Araçagi gi|501225153|ref|WP_012268171.1| 1 McnC protein [Microcystis aeruginosa]

Araçagi gi|817700114|ref|WP_046662633.1| 1 McnC protein [Microcystis aeruginosa]

Araçagi gi|817700114|ref|WP_046662633.1| 1 McnC protein [Microcystis aeruginosa]

Araçagi gi|817700114|ref|WP_046662633.1| 1 McnC protein [Microcystis aeruginosa]

Araçagi gi|817700114|ref|WP_046662633.1| 1 McnC protein [Microcystis aeruginosa]

Araçagi gi|488874710|ref|WP_002786935.1| 1 non-ribosomal peptide synthetase [Microcystis aeruginosa]

Araçagi gi|640535235|ref|WP_024968867.1| 1 non-ribosomal peptide synthetase [Microcystis aeruginosa]

Araçagi gi|490389196|ref|WP_004268651.1| 1 non-ribosomal peptide synthetase [Microcystis aeruginosa]

Araçagi gi|488829433|ref|WP_002741839.1| 1 McnE protein [Microcystis aeruginosa]

Araçagi gi|817700115|ref|WP_046662634.1| 1 non-ribosomal peptide synthetase [Microcystis aeruginosa]

Araçagi gi|640535887|ref|WP_024969393.1| 1 non-ribosomal peptide synthetase [Microcystis aeruginosa]

Araçagi gi|488893013|ref|WP_002804158.1| 1 non-ribosomal peptide synthetase [Microcystis aeruginosa]

Araçagi gi|488839880|ref|WP_002752286.1| 1 non-ribosomal peptide synthetase [Microcystis aeruginosa]

Araçagi gi|652402436|ref|WP_026798232.1| 1 non-ribosomal peptide synthetase [Planktothrix prolifica]

Araçagi gi|652996394|ref|WP_027248861.1| 1 non-ribosomal peptide synthetase [Planktothrix agardhii]

Araçagi gi|640535235|ref|WP_024968867.1| 1 non-ribosomal peptide synthetase [Microcystis aeruginosa]

Araçagi gi|488838375|ref|WP_002750781.1| 1 non-ribosomal peptide synthetase [Microcystis aeruginosa]

Araçagi gi|488879472|ref|WP_002791697.1| 1 non-ribosomal peptide synthetase [Microcystis aeruginosa]

Araçagi gi|488829433|ref|WP_002741839.1| 1 McnE protein [Microcystis aeruginosa]

Araçagi gi|501225152|ref|WP_012268170.1| 1 non-ribosomal peptide synthetase [Microcystis aeruginosa]

Araçagi gi|488830429|ref|WP_002742835.1| 1 non-ribosomal peptide synthase [Microcystis aeruginosa]

Araçagi gi|488884831|ref|WP_002797056.1| 1 McyA protein [Microcystis aeruginosa]

Araçagi gi|1002987251|ref|WP_061431778.1| 1 non-ribosomal peptide synthetase [Microcystis aeruginosa]

Araçagi gi|501223610|ref|WP_012266628.1| 1 McyA protein [Microcystis aeruginosa]

Araçagi gi|490265066|ref|WP_004161726.1| 1 McyA protein [Microcystis aeruginosa]

Araçagi gi|488828768|ref|WP_002741174.1| 1 non-ribosomal peptide synthetase [Microcystis aeruginosa]

Araçagi gi|488876300|ref|WP_002788525.1| 1 non-ribosomal peptide synthetase [Microcystis aeruginosa]

Araçagi gi|640536675|ref|WP_024969848.1| 1 non-ribosomal peptide synthetase [Microcystis aeruginosa]

Araçagi gi|488821608|ref|WP_002734014.1| 1 non-ribosomal peptide synthetase [Microcystis aeruginosa]

Araçagi gi|1065016475|ref|WP_069474085.1| 1 non-ribosomal peptide synthetase [Microcystis aeruginosa]

Araçagi gi|501224905|ref|WP_012267923.1| 1 non-ribosomal peptide synthetase [Microcystis aeruginosa]

Araçagi gi|488881832|ref|WP_002794057.1| 1 non-ribosomal peptide synthetase [Microcystis aeruginosa]

Araçagi gi|640536675|ref|WP_024969848.1| 1 non-ribosomal peptide synthetase [Microcystis aeruginosa]

Araçagi gi|764661239|ref|WP_044447315.1| 1 hypothetical protein [Mastigocladus laminosus]

Araçagi gi|488864203|ref|WP_002776442.1| 1 non-ribosomal peptide synthetase [Microcystis aeruginosa]

Araçagi gi|763115833|ref|WP_043995747.1| 1 McyC protein [Microcystis aeruginosa]

Araçagi gi|1120049988|ref|WP_072923811.1| 1 non-ribosomal peptide synthetase [Microcystis aeruginosa]

Araçagi gi|738449165|ref|WP_036400352.1| 1 McyC protein [Microcystis aeruginosa]

Araçagi gi|763116736|ref|WP_043996650.1| 1 McyC protein [Microcystis aeruginosa]

Araçagi gi|501377217|ref|WP_012408783.1| 1 non-ribosomal peptide synthetase [Nostoc punctiforme]

Araçagi gi|501377217|ref|WP_012408783.1| 1 non-ribosomal peptide synthetase [Nostoc punctiforme]

Araçagi gi|501377217|ref|WP_012408783.1| 1 non-ribosomal peptide synthetase [Nostoc punctiforme]

Araçagi gi|501377217|ref|WP_012408783.1| 1 non-ribosomal peptide synthetase [Nostoc punctiforme]

Araçagi gi|1060047545|ref|WP_069074216.1| 1 hypothetical protein [Nostoc sp. KVJ20]

Araçagi gi|1060047545|ref|WP_069074216.1| 1 hypothetical protein [Nostoc sp. KVJ20]

Araçagi gi|501377220|ref|WP_012408786.1| 1 non-ribosomal peptide synthetase [Nostoc punctiforme]

Araçagi gi|501377220|ref|WP_012408786.1| 1 non-ribosomal peptide synthetase [Nostoc punctiforme]

Araçagi gi|1121319243|ref|WP_073629382.1| 1 hypothetical protein [Scytonema sp. HK-05]

Araçagi gi|1121319237|ref|WP_073629376.1| 1 non-ribosomal peptide synthetase [Scytonema sp. HK-05]

Araçagi gi|1121319237|ref|WP_073629376.1| 1 non-ribosomal peptide synthetase [Scytonema sp. HK-05]

Araçagi gi|1121319237|ref|WP_073629376.1| 1 non-ribosomal peptide synthetase [Scytonema sp. HK-05]

Araçagi gi|763155072|ref|WP_044034730.1| 1 non-ribosomal peptide synthetase

Araçagi gi|501224905|ref|WP_012267923.1| 1 non-ribosomal peptide synthetase [Microcystis aeruginosa]

Araçagi gi|640536675|ref|WP_024969848.1| 1 non-ribosomal peptide synthetase [Microcystis aeruginosa]

Araçagi gi|488881832|ref|WP_002794057.1| 1 non-ribosomal peptide synthetase [Microcystis aeruginosa]

Araçagi gi|488828768|ref|WP_002741174.1| 1 non-ribosomal peptide synthetase [Microcystis aeruginosa]

Araçagi gi|546229791|ref|WP_021833816.1| 1 type I polyketide synthase [Crocosphaera watsonii]

Araçagi gi|916237525|ref|WP_050977909.1| 1 type I polyketide synthase

Araçagi gi|1132235572|ref|WP_075903736.1| 1 hypothetical protein [Moorea bouillonii]

Araçagi gi|1132235572|ref|WP_075903736.1| 1 hypothetical protein [Moorea bouillonii]

Araçagi gi|910240674|ref|WP_050045665.1| 1 hypothetical protein [Tolypothrix bouteillei]

Araçagi gi|488829431|ref|WP_002741837.1| 1 McnC protein [Microcystis aeruginosa]

Araçagi gi|1120050422|ref|WP_072924245.1| 1 non-ribosomal peptide synthetase [Microcystis aeruginosa]

Araçagi gi|488864797|ref|WP_002777036.1| 1 McnC protein [Microcystis aeruginosa]

Araçagi gi|488879470|ref|WP_002791695.1| 1 non-ribosomal peptide synthetase [Microcystis aeruginosa]

Araçagi gi|1002985852|ref|WP_061430379.1| 1 non-ribosomal peptide synthetase [Microcystis aeruginosa]

Araçagi gi|513836606|ref|WP_016514303.1| 1 non-ribosomal peptide synthetase [Microcystis aeruginosa]

Araçagi gi|917761360|ref|WP_052275365.1| 1 non-ribosomal peptide synthetase [Microcystis panniformis]

Araçagi gi|1002987895|ref|WP_061432422.1| 1 non-ribosomal peptide synthetase [Microcystis aeruginosa]

Araçagi gi|488828768|ref|WP_002741174.1| 1 non-ribosomal peptide synthetase [Microcystis aeruginosa]

Araçagi gi|763155072|ref|WP_044034730.1| 1 non-ribosomal peptide synthetase

Araçagi gi|640536675|ref|WP_024969848.1| 1 non-ribosomal peptide synthetase [Microcystis aeruginosa]

Araçagi gi|817698756|ref|WP_046661401.1| 1 non-ribosomal peptide synthetase [Microcystis aeruginosa]

Araçagi gi|488876300|ref|WP_002788525.1| 1 non-ribosomal peptide synthetase [Microcystis aeruginosa]

Araçagi gi|1002987895|ref|WP_061432422.1| 1 non-ribosomal peptide synthetase [Microcystis aeruginosa]

Araçagi gi|488828768|ref|WP_002741174.1| 1 non-ribosomal peptide synthetase [Microcystis aeruginosa]

Araçagi gi|513852747|ref|WP_016517213.1| 1 non-ribosomal peptide synthetase [Microcystis aeruginosa]

Araçagi gi|488849301|ref|WP_002761707.1| 1 McyC protein [Microcystis aeruginosa]

Araçagi gi|501223608|ref|WP_012266626.1| 1 McyC protein [Microcystis aeruginosa]

Araçagi gi|1002987253|ref|WP_061431780.1| 1 non-ribosomal peptide synthetase [Microcystis aeruginosa]

Araçagi gi|763115833|ref|WP_043995747.1| 1 McyC protein [Microcystis aeruginosa]

Araçagi gi|488829431|ref|WP_002741837.1| 1 McnC protein [Microcystis aeruginosa]

Araçagi gi|488829431|ref|WP_002741837.1| 1 McnC protein [Microcystis aeruginosa]

Araçagi gi|488864797|ref|WP_002777036.1| 1 McnC protein [Microcystis aeruginosa]

Araçagi gi|488864797|ref|WP_002777036.1| 1 McnC protein [Microcystis aeruginosa]

Araçagi gi|1120050422|ref|WP_072924245.1| 1 non-ribosomal peptide synthetase [Microcystis aeruginosa]

Araçagi gi|640535236|ref|WP_024968868.1| 1 McnC protein [Microcystis aeruginosa]

Araçagi gi|640535236|ref|WP_024968868.1| 1 McnC protein [Microcystis aeruginosa]

Araçagi gi|488879470|ref|WP_002791695.1| 1 non-ribosomal peptide synthetase [Microcystis aeruginosa]

Araçagi gi|488879470|ref|WP_002791695.1| 1 non-ribosomal peptide synthetase [Microcystis aeruginosa]

Araçagi gi|490265524|ref|WP_004162176.1| 1 non-ribosomal peptide synthetase [Microcystis aeruginosa]

Araçagi gi|504968606|ref|WP_015155708.1| 1 condensation domain-containing protein [Chroococcidiopsis thermalis]

Araçagi gi|497317123|ref|WP_009631336.1| 1 non-ribosomal peptide synthase/amino acid adenylation enzyme [Synechocystis sp. PCC 7509]

Araçagi gi|497317123|ref|WP_009631336.1| 1 non-ribosomal peptide synthase/amino acid adenylation enzyme [Synechocystis sp. PCC 7509]

Araçagi gi|501330418|ref|WP_012362053.1| 1 non-ribosomal peptide synthetase [Cyanothece sp. ATCC 51142]

Araçagi gi|639853762|ref|WP_024750348.1| 1 non-ribosomal peptide synthetase [Cyanothece sp. ATCC 51472]

Araçagi gi|553735111|ref|WP_023069675.1| 1 methyltransferase

Araçagi gi|808793955|ref|WP_046276736.1| 1 type I polyketide synthase [Limnoraphis robusta]

Araçagi gi|505005272|ref|WP_015192374.1| 1 6-deoxyerythronolide-B synthase 8-amino-7-oxononanoate synthase [Stanieria cyanosphaera]

Araçagi gi|553741403|ref|WP_023075560.1| 1 polyketide synthase family protein [Leptolyngbya sp. Heron Island J]

Araçagi gi|495473350|ref|WP_008198038.1| 1 Putative fatty-acid--CoA ligase (Acyl-CoA synthetase) [Microcystis sp. T1-4]

Araçagi gi|1057444374|ref|WP_068790100.1| 1 acyl-CoA synthetase [Phormidium willei]

Araçagi gi|493555044|ref|WP_006508582.1| 1 acyl-CoA synthetase (AMP-forming)/AMP-acid ligase II [Xenococcus sp. PCC 7305]

Araçagi gi|515515854|ref|WP_016949108.1| 1 hypothetical protein [Anabaena sp. PCC 7108]

Araçagi gi|889965730|ref|WP_048868054.1| 1 AMP-dependent synthetase [Scytonema tolypothrichoides]

Araçagi gi|917763913|ref|WP_052277918.1| 1 McyA protein [Microcystis panniformis]

Araçagi gi|488884831|ref|WP_002797056.1| 1 McyA protein [Microcystis aeruginosa]

Araçagi gi|501223610|ref|WP_012266628.1| 1 McyA protein [Microcystis aeruginosa]

Araçagi gi|738446319|ref|WP_036397508.1| 1 McyA protein [Microcystis aeruginosa]

Araçagi gi|1002987251|ref|WP_061431778.1| 1 non-ribosomal peptide synthetase [Microcystis aeruginosa]

Araçagi gi|918157632|ref|WP_052335408.1| 1 hypothetical protein [Tolypothrix sp. PCC 7601]

Araçagi gi|1119342681|ref|WP_072319556.1| 1 hypothetical protein [Microcystis aeruginosa]

Araçagi gi|1119342681|ref|WP_072319556.1| 1 hypothetical protein [Microcystis aeruginosa]

Araçagi gi|515351913|ref|WP_016863946.1| 1 non-ribosomal peptide synthetase

Araçagi gi|488864797|ref|WP_002777036.1| 1 McnC protein [Microcystis aeruginosa]

Araçagi gi|488864797|ref|WP_002777036.1| 1 McnC protein [Microcystis aeruginosa]

Araçagi gi|488864797|ref|WP_002777036.1| 1 McnC protein [Microcystis aeruginosa]

Araçagi gi|488864797|ref|WP_002777036.1| 1 McnC protein [Microcystis aeruginosa]

Araçagi gi|488829431|ref|WP_002741837.1| 1 McnC protein [Microcystis aeruginosa]

Araçagi gi|488829431|ref|WP_002741837.1| 1 McnC protein [Microcystis aeruginosa]

Araçagi gi|488829431|ref|WP_002741837.1| 1 McnC protein [Microcystis aeruginosa]

Araçagi gi|488829431|ref|WP_002741837.1| 1 McnC protein [Microcystis aeruginosa]

Araçagi gi|1120050422|ref|WP_072924245.1| 1 non-ribosomal peptide synthetase [Microcystis aeruginosa]

Araçagi gi|1120050422|ref|WP_072924245.1| 1 non-ribosomal peptide synthetase [Microcystis aeruginosa]

Araçagi gi|1120050422|ref|WP_072924245.1| 1 non-ribosomal peptide synthetase [Microcystis aeruginosa]

Araçagi gi|1120050422|ref|WP_072924245.1| 1 non-ribosomal peptide synthetase [Microcystis aeruginosa]

Araçagi gi|501225153|ref|WP_012268171.1| 1 McnC protein [Microcystis aeruginosa]

Araçagi gi|501225153|ref|WP_012268171.1| 1 McnC protein [Microcystis aeruginosa]

Araçagi gi|501225153|ref|WP_012268171.1| 1 McnC protein [Microcystis aeruginosa]

Araçagi gi|501225153|ref|WP_012268171.1| 1 McnC protein [Microcystis aeruginosa]

Araçagi gi|817700114|ref|WP_046662633.1| 1 McnC protein [Microcystis aeruginosa]

Araçagi gi|817700114|ref|WP_046662633.1| 1 McnC protein [Microcystis aeruginosa]

Araçagi gi|817700114|ref|WP_046662633.1| 1 McnC protein [Microcystis aeruginosa]

Araçagi gi|817700114|ref|WP_046662633.1| 1 McnC protein [Microcystis aeruginosa]

Araçagi gi|488864797|ref|WP_002777036.1| 1 McnC protein [Microcystis aeruginosa]

Araçagi gi|488864797|ref|WP_002777036.1| 1 McnC protein [Microcystis aeruginosa]

Araçagi gi|488864797|ref|WP_002777036.1| 1 McnC protein [Microcystis aeruginosa]

Araçagi gi|488864797|ref|WP_002777036.1| 1 McnC protein [Microcystis aeruginosa]

Araçagi gi|1002985852|ref|WP_061430379.1| 1 non-ribosomal peptide synthetase [Microcystis aeruginosa]

Araçagi gi|1002985852|ref|WP_061430379.1| 1 non-ribosomal peptide synthetase [Microcystis aeruginosa]

Araçagi gi|1002985852|ref|WP_061430379.1| 1 non-ribosomal peptide synthetase [Microcystis aeruginosa]

Araçagi gi|1002985852|ref|WP_061430379.1| 1 non-ribosomal peptide synthetase [Microcystis aeruginosa]

Araçagi gi|490265524|ref|WP_004162176.1| 1 non-ribosomal peptide synthetase [Microcystis aeruginosa]

Araçagi gi|504991553|ref|WP_015178655.1| 1 non-ribosomal peptide synthetase [Oscillatoria nigro-viridis]

Araçagi gi|493684924|ref|WP_006635046.1| 1 non-ribosomal peptide synthetase [Microcoleus vaginatus]

Araçagi gi|738537926|ref|WP_036486324.1| 1 non-ribosomal peptide synthetase [Myxosarcina sp. GI1]

Araçagi gi|1121288172|ref|WP_073600774.1| 1 hypothetical protein [Hydrococcus rivularis]

Araçagi gi|1065016638|ref|WP_069474157.1| 1 non-ribosomal peptide synthetase [Microcystis aeruginosa]

Araçagi gi|513845141|ref|WP_016515103.1| 1 non-ribosomal peptide synthetase [Microcystis aeruginosa]

Araçagi gi|495474985|ref|WP_008199672.1| 1 non-ribosomal peptide synthetase [Microcystis sp. T1-4]

Araçagi gi|763120035|ref|WP_043999949.1| 1 non-ribosomal peptide synthetase [Microcystis aeruginosa]

Araçagi gi|488849077|ref|WP_002761483.1| 1 non-ribosomal peptide synthetase [Microcystis aeruginosa]

Araçagi gi|1002985852|ref|WP_061430379.1| 1 non-ribosomal peptide synthetase [Microcystis aeruginosa]

Araçagi gi|1002985852|ref|WP_061430379.1| 1 non-ribosomal peptide synthetase [Microcystis aeruginosa]

Araçagi gi|1002985852|ref|WP_061430379.1| 1 non-ribosomal peptide synthetase [Microcystis aeruginosa]

Araçagi gi|1002985852|ref|WP_061430379.1| 1 non-ribosomal peptide synthetase [Microcystis aeruginosa]

Araçagi gi|501225153|ref|WP_012268171.1| 1 McnC protein [Microcystis aeruginosa]

Araçagi gi|501225153|ref|WP_012268171.1| 1 McnC protein [Microcystis aeruginosa]

Araçagi gi|501225153|ref|WP_012268171.1| 1 McnC protein [Microcystis aeruginosa]

Araçagi gi|501225153|ref|WP_012268171.1| 1 McnC protein [Microcystis aeruginosa]

Araçagi gi|488829431|ref|WP_002741837.1| 1 McnC protein [Microcystis aeruginosa]

Araçagi gi|488829431|ref|WP_002741837.1| 1 McnC protein [Microcystis aeruginosa]

Araçagi gi|488829431|ref|WP_002741837.1| 1 McnC protein [Microcystis aeruginosa]

Araçagi gi|488829431|ref|WP_002741837.1| 1 McnC protein [Microcystis aeruginosa]

Araçagi gi|640535236|ref|WP_024968868.1| 1 McnC protein [Microcystis aeruginosa]

Araçagi gi|640535236|ref|WP_024968868.1| 1 McnC protein [Microcystis aeruginosa]

Araçagi gi|640535236|ref|WP_024968868.1| 1 McnC protein [Microcystis aeruginosa]

Araçagi gi|640535236|ref|WP_024968868.1| 1 McnC protein [Microcystis aeruginosa]

Araçagi gi|817700114|ref|WP_046662633.1| 1 McnC protein [Microcystis aeruginosa]

Araçagi gi|817700114|ref|WP_046662633.1| 1 McnC protein [Microcystis aeruginosa]

Araçagi gi|817700114|ref|WP_046662633.1| 1 McnC protein [Microcystis aeruginosa]

Araçagi gi|817700114|ref|WP_046662633.1| 1 McnC protein [Microcystis aeruginosa]

Araçagi gi|493684919|ref|WP_006635041.1| 1 non-ribosomal peptide synthetase [Microcoleus vaginatus]

Araçagi gi|1121333592|ref|WP_073641491.1| 1 non-ribosomal peptide synthetase [Nostoc calcicola]

Araçagi gi|516249133|ref|WP_017653096.1| 1 non-ribosomal peptide synthetase [Fortiea contorta]

Araçagi gi|916304314|ref|WP_051039360.1| 1 non-ribosomal peptide synthetase [Chlorogloeopsis fritschii]

Araçagi gi|518326645|ref|WP_019496852.1| 1 non-ribosomal peptide synthetase [Calothrix sp. PCC 7103]

Araçagi gi|490265066|ref|WP_004161726.1| 1 McyA protein [Microcystis aeruginosa]

Araçagi gi|490265066|ref|WP_004161726.1| 1 McyA protein [Microcystis aeruginosa]

Araçagi gi|488884831|ref|WP_002797056.1| 1 McyA protein [Microcystis aeruginosa]

Araçagi gi|488884831|ref|WP_002797056.1| 1 McyA protein [Microcystis aeruginosa]

Araçagi gi|1002987251|ref|WP_061431778.1| 1 non-ribosomal peptide synthetase [Microcystis aeruginosa]

Araçagi gi|1002987251|ref|WP_061431778.1| 1 non-ribosomal peptide synthetase [Microcystis aeruginosa]

Araçagi gi|488830429|ref|WP_002742835.1| 1 non-ribosomal peptide synthase [Microcystis aeruginosa]

Araçagi gi|488830429|ref|WP_002742835.1| 1 non-ribosomal peptide synthase [Microcystis aeruginosa]

Araçagi gi|1120049983|ref|WP_072923806.1| 1 non-ribosomal peptide synthetase [Microcystis aeruginosa]

Araçagi gi|1120049983|ref|WP_072923806.1| 1 non-ribosomal peptide synthetase [Microcystis aeruginosa]

Araçagi gi|488879472|ref|WP_002791697.1| 1 non-ribosomal peptide synthetase [Microcystis aeruginosa]

Araçagi gi|488829433|ref|WP_002741839.1| 1 McnE protein [Microcystis aeruginosa]

Araçagi gi|501225152|ref|WP_012268170.1| 1 non-ribosomal peptide synthetase [Microcystis aeruginosa]

Araçagi gi|488884387|ref|WP_002796612.1| 1 non-ribosomal peptide synthetase [Microcystis aeruginosa]

Araçagi gi|1002985853|ref|WP_061430380.1| 1 non-ribosomal peptide synthetase [Microcystis aeruginosa]

Araçagi gi|652996395|ref|WP_027248862.1| 1 non-ribosomal peptide synthetase [Planktothrix agardhii]

Araçagi gi|652389975|ref|WP_026785823.1| 1 non-ribosomal peptide synthetase [Planktothrix rubescens]

Araçagi gi|640535888|ref|WP_024969394.1| 1 non-ribosomal peptide synthetase [Microcystis aeruginosa]

Araçagi gi|488893012|ref|WP_002804157.1| 1 non-ribosomal peptide synthetase [Microcystis aeruginosa]

Araçagi gi|488839879|ref|WP_002752285.1| 1 non-ribosomal peptide synthetase [Microcystis aeruginosa]

Araçagi gi|763154312|ref|WP_044033970.1| 1 McnB protein [Microcystis aeruginosa]

Araçagi gi|488874708|ref|WP_002786933.1| 1 non-ribosomal peptide synthetase [Microcystis aeruginosa]

Araçagi gi|652996399|ref|WP_027248866.1| 1 non-ribosomal peptide synthetase [Planktothrix agardhii]

Araçagi gi|652996399|ref|WP_027248866.1| 1 non-ribosomal peptide synthetase [Planktothrix agardhii]

Araçagi gi|652996399|ref|WP_027248866.1| 1 non-ribosomal peptide synthetase [Planktothrix agardhii]

Araçagi gi|501225154|ref|WP_012268172.1| 1 McnB protein [Microcystis aeruginosa]

Araçagi gi|817701301|ref|WP_046663793.1| 1 McnB protein [Microcystis aeruginosa]

Araçagi gi|488829431|ref|WP_002741837.1| 1 McnC protein [Microcystis aeruginosa]

Araçagi gi|1120050422|ref|WP_072924245.1| 1 non-ribosomal peptide synthetase [Microcystis aeruginosa]

Araçagi gi|488879470|ref|WP_002791695.1| 1 non-ribosomal peptide synthetase [Microcystis aeruginosa]

Araçagi gi|488864797|ref|WP_002777036.1| 1 McnC protein [Microcystis aeruginosa]

Araçagi gi|1002985852|ref|WP_061430379.1| 1 non-ribosomal peptide synthetase [Microcystis aeruginosa]

Araçagi gi|817700114|ref|WP_046662633.1| 1 McnC protein [Microcystis aeruginosa]

Araçagi gi|817700114|ref|WP_046662633.1| 1 McnC protein [Microcystis aeruginosa]

Araçagi gi|817700114|ref|WP_046662633.1| 1 McnC protein [Microcystis aeruginosa]

Araçagi gi|817700114|ref|WP_046662633.1| 1 McnC protein [Microcystis aeruginosa]

Araçagi gi|1002985852|ref|WP_061430379.1| 1 non-ribosomal peptide synthetase [Microcystis aeruginosa]

Araçagi gi|1002985852|ref|WP_061430379.1| 1 non-ribosomal peptide synthetase [Microcystis aeruginosa]

Araçagi gi|1002985852|ref|WP_061430379.1| 1 non-ribosomal peptide synthetase [Microcystis aeruginosa]

Araçagi gi|1002985852|ref|WP_061430379.1| 1 non-ribosomal peptide synthetase [Microcystis aeruginosa]

Araçagi gi|1120050422|ref|WP_072924245.1| 1 non-ribosomal peptide synthetase [Microcystis aeruginosa]

Araçagi gi|1120050422|ref|WP_072924245.1| 1 non-ribosomal peptide synthetase [Microcystis aeruginosa]

Araçagi gi|1120050422|ref|WP_072924245.1| 1 non-ribosomal peptide synthetase [Microcystis aeruginosa]

Araçagi gi|1120050422|ref|WP_072924245.1| 1 non-ribosomal peptide synthetase [Microcystis aeruginosa]

Araçagi gi|488829431|ref|WP_002741837.1| 1 McnC protein [Microcystis aeruginosa]

Araçagi gi|488829431|ref|WP_002741837.1| 1 McnC protein [Microcystis aeruginosa]

Araçagi gi|488829431|ref|WP_002741837.1| 1 McnC protein [Microcystis aeruginosa]

Araçagi gi|488829431|ref|WP_002741837.1| 1 McnC protein [Microcystis aeruginosa]

Araçagi gi|501225153|ref|WP_012268171.1| 1 McnC protein [Microcystis aeruginosa]

Araçagi gi|501225153|ref|WP_012268171.1| 1 McnC protein [Microcystis aeruginosa]

Araçagi gi|501225153|ref|WP_012268171.1| 1 McnC protein [Microcystis aeruginosa]

Araçagi gi|501225153|ref|WP_012268171.1| 1 McnC protein [Microcystis aeruginosa]

Araçagi gi|488879470|ref|WP_002791695.1| 1 non-ribosomal peptide synthetase [Microcystis aeruginosa]

Araçagi gi|488879470|ref|WP_002791695.1| 1 non-ribosomal peptide synthetase [Microcystis aeruginosa]

Araçagi gi|488879470|ref|WP_002791695.1| 1 non-ribosomal peptide synthetase [Microcystis aeruginosa]

Araçagi gi|488879470|ref|WP_002791695.1| 1 non-ribosomal peptide synthetase [Microcystis aeruginosa]

Araçagi gi|488864797|ref|WP_002777036.1| 1 McnC protein [Microcystis aeruginosa]

Araçagi gi|488864797|ref|WP_002777036.1| 1 McnC protein [Microcystis aeruginosa]

Araçagi gi|488864797|ref|WP_002777036.1| 1 McnC protein [Microcystis aeruginosa]

Araçagi gi|488864797|ref|WP_002777036.1| 1 McnC protein [Microcystis aeruginosa]

Araçagi gi|1120050422|ref|WP_072924245.1| 1 non-ribosomal peptide synthetase [Microcystis aeruginosa]

Araçagi gi|1120050422|ref|WP_072924245.1| 1 non-ribosomal peptide synthetase [Microcystis aeruginosa]

Araçagi gi|1120050422|ref|WP_072924245.1| 1 non-ribosomal peptide synthetase [Microcystis aeruginosa]

Araçagi gi|1120050422|ref|WP_072924245.1| 1 non-ribosomal peptide synthetase [Microcystis aeruginosa]

Araçagi gi|1002985852|ref|WP_061430379.1| 1 non-ribosomal peptide synthetase [Microcystis aeruginosa]

Araçagi gi|1002985852|ref|WP_061430379.1| 1 non-ribosomal peptide synthetase [Microcystis aeruginosa]

Araçagi gi|1002985852|ref|WP_061430379.1| 1 non-ribosomal peptide synthetase [Microcystis aeruginosa]

Araçagi gi|1002985852|ref|WP_061430379.1| 1 non-ribosomal peptide synthetase [Microcystis aeruginosa]

Araçagi gi|817700114|ref|WP_046662633.1| 1 McnC protein [Microcystis aeruginosa]

Araçagi gi|817700114|ref|WP_046662633.1| 1 McnC protein [Microcystis aeruginosa]

Araçagi gi|817700114|ref|WP_046662633.1| 1 McnC protein [Microcystis aeruginosa]

Araçagi gi|817700114|ref|WP_046662633.1| 1 McnC protein [Microcystis aeruginosa]

Araçagi gi|488879470|ref|WP_002791695.1| 1 non-ribosomal peptide synthetase [Microcystis aeruginosa]

Araçagi gi|488879470|ref|WP_002791695.1| 1 non-ribosomal peptide synthetase [Microcystis aeruginosa]

Araçagi gi|488864797|ref|WP_002777036.1| 1 McnC protein [Microcystis aeruginosa]

Araçagi gi|488864797|ref|WP_002777036.1| 1 McnC protein [Microcystis aeruginosa]

Araçagi gi|1002985852|ref|WP_061430379.1| 1 non-ribosomal peptide synthetase [Microcystis aeruginosa]

Araçagi gi|501225153|ref|WP_012268171.1| 1 McnC protein [Microcystis aeruginosa]

Araçagi gi|501225153|ref|WP_012268171.1| 1 McnC protein [Microcystis aeruginosa]

Araçagi gi|817700114|ref|WP_046662633.1| 1 McnC protein [Microcystis aeruginosa]

Araçagi gi|640535236|ref|WP_024968868.1| 1 McnC protein [Microcystis aeruginosa]

Araçagi gi|640535236|ref|WP_024968868.1| 1 McnC protein [Microcystis aeruginosa]

Araçagi gi|640535236|ref|WP_024968868.1| 1 McnC protein [Microcystis aeruginosa]

Araçagi gi|640535236|ref|WP_024968868.1| 1 McnC protein [Microcystis aeruginosa]

Araçagi gi|488864797|ref|WP_002777036.1| 1 McnC protein [Microcystis aeruginosa]

Araçagi gi|488864797|ref|WP_002777036.1| 1 McnC protein [Microcystis aeruginosa]

Araçagi gi|488864797|ref|WP_002777036.1| 1 McnC protein [Microcystis aeruginosa]

Araçagi gi|488864797|ref|WP_002777036.1| 1 McnC protein [Microcystis aeruginosa]

Araçagi gi|1120050422|ref|WP_072924245.1| 1 non-ribosomal peptide synthetase [Microcystis aeruginosa]

Araçagi gi|1120050422|ref|WP_072924245.1| 1 non-ribosomal peptide synthetase [Microcystis aeruginosa]

Araçagi gi|1120050422|ref|WP_072924245.1| 1 non-ribosomal peptide synthetase [Microcystis aeruginosa]

Araçagi gi|1120050422|ref|WP_072924245.1| 1 non-ribosomal peptide synthetase [Microcystis aeruginosa]

Araçagi gi|817700114|ref|WP_046662633.1| 1 McnC protein [Microcystis aeruginosa]

Araçagi gi|817700114|ref|WP_046662633.1| 1 McnC protein [Microcystis aeruginosa]

Araçagi gi|817700114|ref|WP_046662633.1| 1 McnC protein [Microcystis aeruginosa]

Araçagi gi|817700114|ref|WP_046662633.1| 1 McnC protein [Microcystis aeruginosa]

Araçagi gi|488879470|ref|WP_002791695.1| 1 non-ribosomal peptide synthetase [Microcystis aeruginosa]

Araçagi gi|488879470|ref|WP_002791695.1| 1 non-ribosomal peptide synthetase [Microcystis aeruginosa]

Araçagi gi|488879470|ref|WP_002791695.1| 1 non-ribosomal peptide synthetase [Microcystis aeruginosa]

Araçagi gi|488879470|ref|WP_002791695.1| 1 non-ribosomal peptide synthetase [Microcystis aeruginosa]

Araçagi gi|754792451|ref|WP_042156030.1| 1 non-ribosomal peptide synthetase [Planktothrix agardhii]

Araçagi gi|501377451|ref|WP_012409017.1| 1 non-ribosomal peptide synthetase [Nostoc punctiforme]

Araçagi gi|1121332337|ref|WP_073640236.1| 1 non-ribosomal peptide synthetase [Nostoc calcicola]

Araçagi gi|504891876|ref|WP_015078978.1| 1 non-ribosomal peptide synthetase [Anabaena sp. 90]

Araçagi gi|653154930|ref|WP_027403947.1| 1 non-ribosomal peptide synthetase [Aphanizomenon flos-aquae]

Araçagi gi|640535181|ref|WP_024968820.1| 1 hybrid non-ribosomal peptide synthetase/type I polyketide synthase [Microcystis aeruginosa]

Araçagi gi|918210105|ref|WP_052347424.1| 1 hybrid non-ribosomal peptide synthetase/type I polyketide synthase [Planktothrix prolifica]

Araçagi gi|918201475|ref|WP_052338794.1| 1 hybrid non-ribosomal peptide synthetase/type I polyketide synthase [Planktothrix prolifica]

Araçagi gi|1065018840|ref|WP_069475189.1| 1 hypothetical protein [Microcystis aeruginosa]

Araçagi gi|488839866|ref|WP_002752272.1| 1 hybrid non-ribosomal peptide synthetase/type I polyketide synthase [Microcystis aeruginosa]

Araçagi gi|817700114|ref|WP_046662633.1| 1 McnC protein [Microcystis aeruginosa]

Araçagi gi|817700114|ref|WP_046662633.1| 1 McnC protein [Microcystis aeruginosa]

Araçagi gi|817700114|ref|WP_046662633.1| 1 McnC protein [Microcystis aeruginosa]

Araçagi gi|817700114|ref|WP_046662633.1| 1 McnC protein [Microcystis aeruginosa]

Araçagi gi|488864797|ref|WP_002777036.1| 1 McnC protein [Microcystis aeruginosa]

Araçagi gi|488864797|ref|WP_002777036.1| 1 McnC protein [Microcystis aeruginosa]

Araçagi gi|488864797|ref|WP_002777036.1| 1 McnC protein [Microcystis aeruginosa]

Araçagi gi|488864797|ref|WP_002777036.1| 1 McnC protein [Microcystis aeruginosa]

Araçagi gi|1002985852|ref|WP_061430379.1| 1 non-ribosomal peptide synthetase [Microcystis aeruginosa]

Araçagi gi|1002985852|ref|WP_061430379.1| 1 non-ribosomal peptide synthetase [Microcystis aeruginosa]

Araçagi gi|1002985852|ref|WP_061430379.1| 1 non-ribosomal peptide synthetase [Microcystis aeruginosa]

Araçagi gi|1002985852|ref|WP_061430379.1| 1 non-ribosomal peptide synthetase [Microcystis aeruginosa]

Araçagi gi|1120050422|ref|WP_072924245.1| 1 non-ribosomal peptide synthetase [Microcystis aeruginosa]

Araçagi gi|1120050422|ref|WP_072924245.1| 1 non-ribosomal peptide synthetase [Microcystis aeruginosa]

Araçagi gi|1120050422|ref|WP_072924245.1| 1 non-ribosomal peptide synthetase [Microcystis aeruginosa]

Araçagi gi|1120050422|ref|WP_072924245.1| 1 non-ribosomal peptide synthetase [Microcystis aeruginosa]

Araçagi gi|640535236|ref|WP_024968868.1| 1 McnC protein [Microcystis aeruginosa]

Araçagi gi|640535236|ref|WP_024968868.1| 1 McnC protein [Microcystis aeruginosa]

Araçagi gi|640535236|ref|WP_024968868.1| 1 McnC protein [Microcystis aeruginosa]

Araçagi gi|640535236|ref|WP_024968868.1| 1 McnC protein [Microcystis aeruginosa]

Araçagi gi|488874707|ref|WP_002786932.1| 1 McnA protein [Microcystis aeruginosa]

Araçagi gi|738448644|ref|WP_036399831.1| 1 McnA protein [Microcystis aeruginosa]

Araçagi gi|488864799|ref|WP_002777038.1| 1 non-ribosomal peptide synthetase [Microcystis aeruginosa]

Araçagi gi|488879468|ref|WP_002791693.1| 1 non-ribosomal peptide synthetase [Microcystis aeruginosa]

Araçagi gi|501225155|ref|WP_012268173.1| 1 McnA protein [Microcystis aeruginosa]

Araçagi gi|501377948|ref|WP_012409514.1| 1 non-ribosomal peptide synthetase [Nostoc punctiforme]

Araçagi gi|501377948|ref|WP_012409514.1| 1 non-ribosomal peptide synthetase [Nostoc punctiforme]

Araçagi gi|501377948|ref|WP_012409514.1| 1 non-ribosomal peptide synthetase [Nostoc punctiforme]

Araçagi gi|488829431|ref|WP_002741837.1| 1 McnC protein [Microcystis aeruginosa]

Araçagi gi|488829431|ref|WP_002741837.1| 1 McnC protein [Microcystis aeruginosa]

Araçagi gi|488829431|ref|WP_002741837.1| 1 McnC protein [Microcystis aeruginosa]

Araçagi gi|488829431|ref|WP_002741837.1| 1 McnC protein [Microcystis aeruginosa]

Araçagi gi|488864797|ref|WP_002777036.1| 1 McnC protein [Microcystis aeruginosa]

Araçagi gi|488864797|ref|WP_002777036.1| 1 McnC protein [Microcystis aeruginosa]

Araçagi gi|488864797|ref|WP_002777036.1| 1 McnC protein [Microcystis aeruginosa]

Araçagi gi|488864797|ref|WP_002777036.1| 1 McnC protein [Microcystis aeruginosa]

Araçagi gi|817700114|ref|WP_046662633.1| 1 McnC protein [Microcystis aeruginosa]

Araçagi gi|817700114|ref|WP_046662633.1| 1 McnC protein [Microcystis aeruginosa]

Araçagi gi|817700114|ref|WP_046662633.1| 1 McnC protein [Microcystis aeruginosa]

Araçagi gi|817700114|ref|WP_046662633.1| 1 McnC protein [Microcystis aeruginosa]

Araçagi gi|1002985852|ref|WP_061430379.1| 1 non-ribosomal peptide synthetase [Microcystis aeruginosa]

Araçagi gi|1002985852|ref|WP_061430379.1| 1 non-ribosomal peptide synthetase [Microcystis aeruginosa]

Araçagi gi|1002985852|ref|WP_061430379.1| 1 non-ribosomal peptide synthetase [Microcystis aeruginosa]

Araçagi gi|1002985852|ref|WP_061430379.1| 1 non-ribosomal peptide synthetase [Microcystis aeruginosa]

Araçagi gi|1120050422|ref|WP_072924245.1| 1 non-ribosomal peptide synthetase [Microcystis aeruginosa]

Araçagi gi|1120050422|ref|WP_072924245.1| 1 non-ribosomal peptide synthetase [Microcystis aeruginosa]

Araçagi gi|1120050422|ref|WP_072924245.1| 1 non-ribosomal peptide synthetase [Microcystis aeruginosa]

Araçagi gi|1120050422|ref|WP_072924245.1| 1 non-ribosomal peptide synthetase [Microcystis aeruginosa]

Araçagi gi|1105152502|ref|WP_071823928.1| 1 hypothetical protein [Microcystis aeruginosa]

Araçagi gi|652326501|ref|WP_026723819.1| 1 non-ribosomal peptide synthase [Fischerella sp. PCC 9431]

Araçagi gi|1027926135|ref|WP_063800724.1| 1 non-ribosomal peptide synthetase [Mastigocoleus testarum]

Araçagi gi|1121309678|ref|WP_073621082.1| 1 non-ribosomal peptide synthetase [Calothrix sp. HK-06]

Araçagi gi|501378046|ref|WP_012409612.1| 1 non-ribosomal peptide synthetase [Nostoc punctiforme]

Araçagi gi|1002987895|ref|WP_061432422.1| 1 non-ribosomal peptide synthetase [Microcystis aeruginosa]

Araçagi gi|917761360|ref|WP_052275365.1| 1 non-ribosomal peptide synthetase [Microcystis panniformis]

Araçagi gi|817698756|ref|WP_046661401.1| 1 non-ribosomal peptide synthetase [Microcystis aeruginosa]

Araçagi gi|640536675|ref|WP_024969848.1| 1 non-ribosomal peptide synthetase [Microcystis aeruginosa]

Araçagi gi|1120051153|ref|WP_072924976.1| 1 non-ribosomal peptide synthetase [Microcystis aeruginosa]

Araçagi gi|1084331020|ref|WP_070395079.1| 1 hypothetical protein [Moorea producens]

Araçagi gi|493036824|ref|WP_006104235.1| 1 non-ribosomal peptide synthetase [Coleofasciculus chthonoplastes]

Araçagi gi|495465771|ref|WP_008190462.1| 1 non-ribosomal peptide synthetase [Moorea producens]

Araçagi gi|918439317|ref|WP_052490493.1| 1 non-ribosomal peptide synthetase [Tolypothrix campylonemoides]

Araçagi gi|918439317|ref|WP_052490493.1| 1 non-ribosomal peptide synthetase [Tolypothrix campylonemoides]

Araçagi gi|516326924|ref|WP_017717583.1| 1 hypothetical protein [Oscillatoria sp. PCC 10802]

Araçagi gi|737165129|ref|WP_035151548.1| 1 acyl-CoA synthetase [Calothrix sp. 336/3]

Araçagi gi|488864797|ref|WP_002777036.1| 1 McnC protein [Microcystis aeruginosa]

Araçagi gi|488864797|ref|WP_002777036.1| 1 McnC protein [Microcystis aeruginosa]

Araçagi gi|488864797|ref|WP_002777036.1| 1 McnC protein [Microcystis aeruginosa]

Araçagi gi|488864797|ref|WP_002777036.1| 1 McnC protein [Microcystis aeruginosa]

Araçagi gi|488879470|ref|WP_002791695.1| 1 non-ribosomal peptide synthetase [Microcystis aeruginosa]

Araçagi gi|488879470|ref|WP_002791695.1| 1 non-ribosomal peptide synthetase [Microcystis aeruginosa]

Araçagi gi|488879470|ref|WP_002791695.1| 1 non-ribosomal peptide synthetase [Microcystis aeruginosa]

Araçagi gi|488879470|ref|WP_002791695.1| 1 non-ribosomal peptide synthetase [Microcystis aeruginosa]

Araçagi gi|1002985852|ref|WP_061430379.1| 1 non-ribosomal peptide synthetase [Microcystis aeruginosa]

Araçagi gi|1002985852|ref|WP_061430379.1| 1 non-ribosomal peptide synthetase [Microcystis aeruginosa]

Araçagi gi|1002985852|ref|WP_061430379.1| 1 non-ribosomal peptide synthetase [Microcystis aeruginosa]

Araçagi gi|1002985852|ref|WP_061430379.1| 1 non-ribosomal peptide synthetase [Microcystis aeruginosa]

Araçagi gi|1120050422|ref|WP_072924245.1| 1 non-ribosomal peptide synthetase [Microcystis aeruginosa]

Araçagi gi|1120050422|ref|WP_072924245.1| 1 non-ribosomal peptide synthetase [Microcystis aeruginosa]

Araçagi gi|1120050422|ref|WP_072924245.1| 1 non-ribosomal peptide synthetase [Microcystis aeruginosa]

Araçagi gi|1120050422|ref|WP_072924245.1| 1 non-ribosomal peptide synthetase [Microcystis aeruginosa]

Araçagi gi|817700114|ref|WP_046662633.1| 1 McnC protein [Microcystis aeruginosa]

Araçagi gi|817700114|ref|WP_046662633.1| 1 McnC protein [Microcystis aeruginosa]

Araçagi gi|817700114|ref|WP_046662633.1| 1 McnC protein [Microcystis aeruginosa]

Araçagi gi|817700114|ref|WP_046662633.1| 1 McnC protein [Microcystis aeruginosa]

Araçagi gi|754792451|ref|WP_042156030.1| 1 non-ribosomal peptide synthetase [Planktothrix agardhii]

Araçagi gi|754792451|ref|WP_042156030.1| 1 non-ribosomal peptide synthetase [Planktothrix agardhii]

Araçagi gi|653154930|ref|WP_027403947.1| 1 non-ribosomal peptide synthetase [Aphanizomenon flos-aquae]

Araçagi gi|653154930|ref|WP_027403947.1| 1 non-ribosomal peptide synthetase [Aphanizomenon flos-aquae]

Araçagi gi|504891876|ref|WP_015078978.1| 1 non-ribosomal peptide synthetase [Anabaena sp. 90]

Araçagi gi|504891876|ref|WP_015078978.1| 1 non-ribosomal peptide synthetase [Anabaena sp. 90]

Araçagi gi|1121332337|ref|WP_073640236.1| 1 non-ribosomal peptide synthetase [Nostoc calcicola]

Araçagi gi|1121332337|ref|WP_073640236.1| 1 non-ribosomal peptide synthetase [Nostoc calcicola]

Araçagi gi|1060038991|ref|WP_069068160.1| 1 non-ribosomal peptide synthetase [Nostoc sp. KVJ20]

Araçagi gi|1060038991|ref|WP_069068160.1| 1 non-ribosomal peptide synthetase [Nostoc sp. KVJ20]

Araçagi gi|763115833|ref|WP_043995747.1| 1 McyC protein [Microcystis aeruginosa]

Araçagi gi|1120049988|ref|WP_072923811.1| 1 non-ribosomal peptide synthetase [Microcystis aeruginosa]

Araçagi gi|501223608|ref|WP_012266626.1| 1 McyC protein [Microcystis aeruginosa]

Araçagi gi|513852747|ref|WP_016517213.1| 1 non-ribosomal peptide synthetase [Microcystis aeruginosa]

Araçagi gi|763116736|ref|WP_043996650.1| 1 McyC protein [Microcystis aeruginosa]

Araçagi gi|917763913|ref|WP_052277918.1| 1 McyA protein [Microcystis panniformis]

Araçagi gi|738446319|ref|WP_036397508.1| 1 McyA protein [Microcystis aeruginosa]

Araçagi gi|488830429|ref|WP_002742835.1| 1 non-ribosomal peptide synthase [Microcystis aeruginosa]

Araçagi gi|1120049983|ref|WP_072923806.1| 1 non-ribosomal peptide synthetase [Microcystis aeruginosa]

Araçagi gi|488884831|ref|WP_002797056.1| 1 McyA protein [Microcystis aeruginosa]

Araçagi gi|488829431|ref|WP_002741837.1| 1 McnC protein [Microcystis aeruginosa]

Araçagi gi|1002985852|ref|WP_061430379.1| 1 non-ribosomal peptide synthetase [Microcystis aeruginosa]

Araçagi gi|1120050422|ref|WP_072924245.1| 1 non-ribosomal peptide synthetase [Microcystis aeruginosa]

Araçagi gi|817700114|ref|WP_046662633.1| 1 McnC protein [Microcystis aeruginosa]

Araçagi gi|501225153|ref|WP_012268171.1| 1 McnC protein [Microcystis aeruginosa]

Araçagi gi|754792450|ref|WP_042156029.1| 1 non-ribosomal peptide synthetase [Planktothrix agardhii]

Araçagi gi|488893016|ref|WP_002804161.1| 1 non-ribosomal peptide synthetase [Microcystis aeruginosa]

Araçagi gi|488839883|ref|WP_002752289.1| 1 non-ribosomal peptide synthetase [Microcystis aeruginosa]

Araçagi gi|653003256|ref|WP_027255442.1| 1 non-ribosomal peptide synthetase [Planktothrix agardhii]

Araçagi gi|652389972|ref|WP_026785820.1| 1 non-ribosomal peptide synthetase [Planktothrix rubescens]

Araçagi gi|917763686|ref|WP_052277691.1| 1 MULTISPECIES: peptide synthetase [Microcystis]

Araçagi gi|779876898|ref|WP_045359195.1| 1 peptide synthetase [Microcystis aeruginosa]

Araçagi gi|488877749|ref|WP_002789974.1| 1 peptide synthetase [Microcystis aeruginosa]

Araçagi gi|488849510|ref|WP_002761916.1| 1 peptide synthetase [Microcystis aeruginosa]

Araçagi gi|501222817|ref|WP_012265835.1| 1 peptide synthetase [Microcystis aeruginosa]

Araçagi gi|488874710|ref|WP_002786935.1| 1 non-ribosomal peptide synthetase [Microcystis aeruginosa]

Araçagi gi|488829433|ref|WP_002741839.1| 1 McnE protein [Microcystis aeruginosa]

Araçagi gi|640535235|ref|WP_024968867.1| 1 non-ribosomal peptide synthetase [Microcystis aeruginosa]

Araçagi gi|501225152|ref|WP_012268170.1| 1 non-ribosomal peptide synthetase [Microcystis aeruginosa]

Araçagi gi|1120050425|ref|WP_072924248.1| 1 non-ribosomal peptide synthetase [Microcystis aeruginosa]

Araçagi gi|488877749|ref|WP_002789974.1| 1 peptide synthetase [Microcystis aeruginosa]

Araçagi gi|917763686|ref|WP_052277691.1| 1 MULTISPECIES: peptide synthetase [Microcystis]

Araçagi gi|488849510|ref|WP_002761916.1| 1 peptide synthetase [Microcystis aeruginosa]

Araçagi gi|779876898|ref|WP_045359195.1| 1 peptide synthetase [Microcystis aeruginosa]

Araçagi gi|495482306|ref|WP_008206993.1| 1 peptide synthetase [Microcystis sp. T1-4]

Araçagi gi|488839879|ref|WP_002752285.1| 1 non-ribosomal peptide synthetase [Microcystis aeruginosa]

Araçagi gi|488893012|ref|WP_002804157.1| 1 non-ribosomal peptide synthetase [Microcystis aeruginosa]

Araçagi gi|640535888|ref|WP_024969394.1| 1 non-ribosomal peptide synthetase [Microcystis aeruginosa]

Araçagi gi|754792454|ref|WP_042156033.1| 1 non-ribosomal peptide synthetase [Planktothrix agardhii]

Araçagi gi|652399812|ref|WP_026795614.1| 1 MULTISPECIES: non-ribosomal peptide synthetase [Planktothrix]

Araçagi gi|763115833|ref|WP_043995747.1| 1 McyC protein [Microcystis aeruginosa]

Araçagi gi|513852747|ref|WP_016517213.1| 1 non-ribosomal peptide synthetase [Microcystis aeruginosa]

Araçagi gi|1120049988|ref|WP_072923811.1| 1 non-ribosomal peptide synthetase [Microcystis aeruginosa]

Araçagi gi|501223608|ref|WP_012266626.1| 1 McyC protein [Microcystis aeruginosa]

Araçagi gi|763116736|ref|WP_043996650.1| 1 McyC protein [Microcystis aeruginosa]

Araçagi gi|515877203|ref|WP_017307786.1| 1 non-ribosomal peptide synthetase [Fischerella sp. PCC 9339]

Araçagi gi|889965730|ref|WP_048868054.1| 1 AMP-dependent synthetase [Scytonema tolypothrichoides]

Araçagi gi|504950583|ref|WP_015137685.1| 1 acyl-CoA synthetase [Nostoc sp. PCC 7524]

Araçagi gi|516355639|ref|WP_017745672.1| 1 non-ribosomal peptide synthetase [Scytonema hofmannii]

Araçagi gi|516355639|ref|WP_017745672.1| 1 non-ribosomal peptide synthetase [Scytonema hofmannii]

Araçagi gi|653003249|ref|WP_027255435.1| 1 McnC protein [Planktothrix agardhii]

Araçagi gi|653003249|ref|WP_027255435.1| 1 McnC protein [Planktothrix agardhii]

Araçagi gi|653003249|ref|WP_027255435.1| 1 McnC protein [Planktothrix agardhii]

Araçagi gi|653003249|ref|WP_027255435.1| 1 McnC protein [Planktothrix agardhii]

Araçagi gi|653003249|ref|WP_027255435.1| 1 McnC protein [Planktothrix agardhii]

Araçagi gi|653003249|ref|WP_027255435.1| 1 McnC protein [Planktothrix agardhii]

Araçagi gi|653003249|ref|WP_027255435.1| 1 McnC protein [Planktothrix agardhii]

Araçagi gi|653003249|ref|WP_027255435.1| 1 McnC protein [Planktothrix agardhii]

Araçagi gi|652997906|ref|WP_027250316.1| 1 McnC protein [Planktothrix agardhii]

Araçagi gi|652997906|ref|WP_027250316.1| 1 McnC protein [Planktothrix agardhii]

Araçagi gi|652997906|ref|WP_027250316.1| 1 McnC protein [Planktothrix agardhii]

Araçagi gi|652997906|ref|WP_027250316.1| 1 McnC protein [Planktothrix agardhii]

Araçagi gi|652997906|ref|WP_027250316.1| 1 McnC protein [Planktothrix agardhii]

Araçagi gi|652997906|ref|WP_027250316.1| 1 McnC protein [Planktothrix agardhii]

Araçagi gi|652997906|ref|WP_027250316.1| 1 McnC protein [Planktothrix agardhii]

Araçagi gi|652997906|ref|WP_027250316.1| 1 McnC protein [Planktothrix agardhii]

Araçagi gi|652402732|ref|WP_026798523.1| 1 McnC protein [Planktothrix prolifica]

Araçagi gi|652402732|ref|WP_026798523.1| 1 McnC protein [Planktothrix prolifica]

Araçagi gi|652402732|ref|WP_026798523.1| 1 McnC protein [Planktothrix prolifica]

Araçagi gi|652402732|ref|WP_026798523.1| 1 McnC protein [Planktothrix prolifica]

Araçagi gi|652402732|ref|WP_026798523.1| 1 McnC protein [Planktothrix prolifica]

Araçagi gi|652402732|ref|WP_026798523.1| 1 McnC protein [Planktothrix prolifica]

Araçagi gi|652402732|ref|WP_026798523.1| 1 McnC protein [Planktothrix prolifica]

Araçagi gi|652402732|ref|WP_026798523.1| 1 McnC protein [Planktothrix prolifica]

Araçagi gi|652996400|ref|WP_027248867.1| 1 McnC protein [Planktothrix agardhii]

Araçagi gi|652996400|ref|WP_027248867.1| 1 McnC protein [Planktothrix agardhii]

Araçagi gi|652996400|ref|WP_027248867.1| 1 McnC protein [Planktothrix agardhii]

Araçagi gi|652996400|ref|WP_027248867.1| 1 McnC protein [Planktothrix agardhii]

Araçagi gi|652996400|ref|WP_027248867.1| 1 McnC protein [Planktothrix agardhii]

Araçagi gi|652996400|ref|WP_027248867.1| 1 McnC protein [Planktothrix agardhii]

Araçagi gi|652996400|ref|WP_027248867.1| 1 McnC protein [Planktothrix agardhii]

Araçagi gi|652996400|ref|WP_027248867.1| 1 McnC protein [Planktothrix agardhii]

Araçagi gi|488879470|ref|WP_002791695.1| 1 non-ribosomal peptide synthetase [Microcystis aeruginosa]

Araçagi gi|488879470|ref|WP_002791695.1| 1 non-ribosomal peptide synthetase [Microcystis aeruginosa]

Araçagi gi|488879470|ref|WP_002791695.1| 1 non-ribosomal peptide synthetase [Microcystis aeruginosa]

Araçagi gi|488879470|ref|WP_002791695.1| 1 non-ribosomal peptide synthetase [Microcystis aeruginosa]

Araçagi gi|488879470|ref|WP_002791695.1| 1 non-ribosomal peptide synthetase [Microcystis aeruginosa]

Araçagi gi|488879470|ref|WP_002791695.1| 1 non-ribosomal peptide synthetase [Microcystis aeruginosa]

Araçagi gi|488879470|ref|WP_002791695.1| 1 non-ribosomal peptide synthetase [Microcystis aeruginosa]

Araçagi gi|1120050422|ref|WP_072924245.1| 1 non-ribosomal peptide synthetase [Microcystis aeruginosa]

Araçagi gi|1120050422|ref|WP_072924245.1| 1 non-ribosomal peptide synthetase [Microcystis aeruginosa]

Araçagi gi|1120050422|ref|WP_072924245.1| 1 non-ribosomal peptide synthetase [Microcystis aeruginosa]

Araçagi gi|1120050422|ref|WP_072924245.1| 1 non-ribosomal peptide synthetase [Microcystis aeruginosa]

Araçagi gi|640535236|ref|WP_024968868.1| 1 McnC protein [Microcystis aeruginosa]

Araçagi gi|640535236|ref|WP_024968868.1| 1 McnC protein [Microcystis aeruginosa]

Araçagi gi|640535236|ref|WP_024968868.1| 1 McnC protein [Microcystis aeruginosa]

Araçagi gi|640535236|ref|WP_024968868.1| 1 McnC protein [Microcystis aeruginosa]

Araçagi gi|817700114|ref|WP_046662633.1| 1 McnC protein [Microcystis aeruginosa]

Araçagi gi|817700114|ref|WP_046662633.1| 1 McnC protein [Microcystis aeruginosa]

Araçagi gi|817700114|ref|WP_046662633.1| 1 McnC protein [Microcystis aeruginosa]

Araçagi gi|817700114|ref|WP_046662633.1| 1 McnC protein [Microcystis aeruginosa]

Araçagi gi|488829431|ref|WP_002741837.1| 1 McnC protein [Microcystis aeruginosa]

Araçagi gi|488829431|ref|WP_002741837.1| 1 McnC protein [Microcystis aeruginosa]

Araçagi gi|488829431|ref|WP_002741837.1| 1 McnC protein [Microcystis aeruginosa]

Araçagi gi|488829431|ref|WP_002741837.1| 1 McnC protein [Microcystis aeruginosa]

Araçagi gi|501225153|ref|WP_012268171.1| 1 McnC protein [Microcystis aeruginosa]

Araçagi gi|501225153|ref|WP_012268171.1| 1 McnC protein [Microcystis aeruginosa]

Araçagi gi|501225153|ref|WP_012268171.1| 1 McnC protein [Microcystis aeruginosa]

Araçagi gi|501225153|ref|WP_012268171.1| 1 McnC protein [Microcystis aeruginosa]

Araçagi gi|490265066|ref|WP_004161726.1| 1 McyA protein [Microcystis aeruginosa]

Araçagi gi|488830429|ref|WP_002742835.1| 1 non-ribosomal peptide synthase [Microcystis aeruginosa]

Araçagi gi|917763913|ref|WP_052277918.1| 1 McyA protein [Microcystis panniformis]

Araçagi gi|1120049983|ref|WP_072923806.1| 1 non-ribosomal peptide synthetase [Microcystis aeruginosa]

Araçagi gi|1002987251|ref|WP_061431778.1| 1 non-ribosomal peptide synthetase [Microcystis aeruginosa]

Araçagi gi|918157302|ref|WP_052335078.1| 1 non-ribosomal peptide synthetase [Tolypothrix sp. PCC 7601]

Araçagi gi|1062957830|ref|WP_069347110.1| 1 hypothetical protein [Scytonema millei]

Araçagi gi|501225153|ref|WP_012268171.1| 1 McnC protein [Microcystis aeruginosa]

Araçagi gi|501225153|ref|WP_012268171.1| 1 McnC protein [Microcystis aeruginosa]

Araçagi gi|501225153|ref|WP_012268171.1| 1 McnC protein [Microcystis aeruginosa]

Araçagi gi|501225153|ref|WP_012268171.1| 1 McnC protein [Microcystis aeruginosa]

Araçagi gi|1120050422|ref|WP_072924245.1| 1 non-ribosomal peptide synthetase [Microcystis aeruginosa]

Araçagi gi|1120050422|ref|WP_072924245.1| 1 non-ribosomal peptide synthetase [Microcystis aeruginosa]

Araçagi gi|1120050422|ref|WP_072924245.1| 1 non-ribosomal peptide synthetase [Microcystis aeruginosa]

Araçagi gi|1120050422|ref|WP_072924245.1| 1 non-ribosomal peptide synthetase [Microcystis aeruginosa]

Araçagi gi|488864797|ref|WP_002777036.1| 1 McnC protein [Microcystis aeruginosa]

Araçagi gi|488864797|ref|WP_002777036.1| 1 McnC protein [Microcystis aeruginosa]

Araçagi gi|488864797|ref|WP_002777036.1| 1 McnC protein [Microcystis aeruginosa]

Araçagi gi|488864797|ref|WP_002777036.1| 1 McnC protein [Microcystis aeruginosa]

Araçagi gi|817700114|ref|WP_046662633.1| 1 McnC protein [Microcystis aeruginosa]

Araçagi gi|817700114|ref|WP_046662633.1| 1 McnC protein [Microcystis aeruginosa]

Araçagi gi|817700114|ref|WP_046662633.1| 1 McnC protein [Microcystis aeruginosa]

Araçagi gi|817700114|ref|WP_046662633.1| 1 McnC protein [Microcystis aeruginosa]

Araçagi gi|640535236|ref|WP_024968868.1| 1 McnC protein [Microcystis aeruginosa]

Araçagi gi|640535236|ref|WP_024968868.1| 1 McnC protein [Microcystis aeruginosa]

Araçagi gi|640535236|ref|WP_024968868.1| 1 McnC protein [Microcystis aeruginosa]

Araçagi gi|640535236|ref|WP_024968868.1| 1 McnC protein [Microcystis aeruginosa]

Araçagi gi|515877975|ref|WP_017308558.1| 1 non-ribosomal peptide synthetase [Fischerella sp. PCC 9339]

Araçagi gi|515877975|ref|WP_017308558.1| 1 non-ribosomal peptide synthetase [Fischerella sp. PCC 9339]

Araçagi gi|752819044|ref|WP_041458258.1| 1 McyA protein [Anabaena sp. 90]

Araçagi gi|752819044|ref|WP_041458258.1| 1 McyA protein [Anabaena sp. 90]

Araçagi gi|1117801540|ref|WP_072160736.1| 1 hypothetical protein [Planktothricoides sp. SR001]

Araçagi gi|1117801540|ref|WP_072160736.1| 1 hypothetical protein [Planktothricoides sp. SR001]

Araçagi gi|1121321460|ref|WP_073631599.1| 1 non-ribosomal peptide synthetase [Scytonema sp. HK-05]

Araçagi gi|515889842|ref|WP_017320425.1| 1 non-ribosomal peptide synthetase [cyanobacterium PCC 7702]

Araçagi gi|488839883|ref|WP_002752289.1| 1 non-ribosomal peptide synthetase [Microcystis aeruginosa]

Araçagi gi|488879470|ref|WP_002791695.1| 1 non-ribosomal peptide synthetase [Microcystis aeruginosa]

Araçagi gi|488879470|ref|WP_002791695.1| 1 non-ribosomal peptide synthetase [Microcystis aeruginosa]

Araçagi gi|488829431|ref|WP_002741837.1| 1 McnC protein [Microcystis aeruginosa]

Araçagi gi|488829431|ref|WP_002741837.1| 1 McnC protein [Microcystis aeruginosa]

Araçagi gi|488864797|ref|WP_002777036.1| 1 McnC protein [Microcystis aeruginosa]

Araçagi gi|488864797|ref|WP_002777036.1| 1 McnC protein [Microcystis aeruginosa]

Araçagi gi|652399809|ref|WP_026795611.1| 1 MULTISPECIES: non-ribosomal peptide synthetase [Planktothrix]

Araçagi gi|504966882|ref|WP_015153984.1| 1 non-ribosomal peptide synthetase [Chroococcidiopsis thermalis]

Araçagi gi|488829431|ref|WP_002741837.1| 1 McnC protein [Microcystis aeruginosa]

Araçagi gi|488829431|ref|WP_002741837.1| 1 McnC protein [Microcystis aeruginosa]

Araçagi gi|488829431|ref|WP_002741837.1| 1 McnC protein [Microcystis aeruginosa]

Araçagi gi|488829431|ref|WP_002741837.1| 1 McnC protein [Microcystis aeruginosa]

Araçagi gi|501225153|ref|WP_012268171.1| 1 McnC protein [Microcystis aeruginosa]

Araçagi gi|501225153|ref|WP_012268171.1| 1 McnC protein [Microcystis aeruginosa]

Araçagi gi|501225153|ref|WP_012268171.1| 1 McnC protein [Microcystis aeruginosa]

Araçagi gi|501225153|ref|WP_012268171.1| 1 McnC protein [Microcystis aeruginosa]

Araçagi gi|652996400|ref|WP_027248867.1| 1 McnC protein [Planktothrix agardhii]

Araçagi gi|652996400|ref|WP_027248867.1| 1 McnC protein [Planktothrix agardhii]

Araçagi gi|652996400|ref|WP_027248867.1| 1 McnC protein [Planktothrix agardhii]

Araçagi gi|652996400|ref|WP_027248867.1| 1 McnC protein [Planktothrix agardhii]

Araçagi gi|488864797|ref|WP_002777036.1| 1 McnC protein [Microcystis aeruginosa]

Araçagi gi|488864797|ref|WP_002777036.1| 1 McnC protein [Microcystis aeruginosa]

Araçagi gi|488864797|ref|WP_002777036.1| 1 McnC protein [Microcystis aeruginosa]

Araçagi gi|488864797|ref|WP_002777036.1| 1 McnC protein [Microcystis aeruginosa]

Araçagi gi|754792464|ref|WP_042156042.1| 1 McnC protein [Planktothrix agardhii]

Araçagi gi|754792464|ref|WP_042156042.1| 1 McnC protein [Planktothrix agardhii]

Araçagi gi|754792464|ref|WP_042156042.1| 1 McnC protein [Planktothrix agardhii]

Araçagi gi|754792464|ref|WP_042156042.1| 1 McnC protein [Planktothrix agardhii]

Boqueirão gi|516352623|ref|WP_017742656.1| 1 non-ribosomal peptide synthetase [Scytonema hofmannii]

Boqueirão gi|516352623|ref|WP_017742656.1| 1 non-ribosomal peptide synthetase [Scytonema hofmannii]

Boqueirão gi|516352623|ref|WP_017742656.1| 1 non-ribosomal peptide synthetase [Scytonema hofmannii]

Boqueirão gi|516352615|ref|WP_017742648.1| 1 non-ribosomal peptide synthetase [Scytonema hofmannii]

Boqueirão gi|516352615|ref|WP_017742648.1| 1 non-ribosomal peptide synthetase [Scytonema hofmannii]

Boqueirão gi|516352615|ref|WP_017742648.1| 1 non-ribosomal peptide synthetase [Scytonema hofmannii]

Boqueirão gi|516352615|ref|WP_017742648.1| 1 non-ribosomal peptide synthetase [Scytonema hofmannii]

Boqueirão gi|504892624|ref|WP_015079726.1| 1 anabaenopeptilide synthetase ApdB [Anabaena sp. 90]

Boqueirão gi|504892624|ref|WP_015079726.1| 1 anabaenopeptilide synthetase ApdB [Anabaena sp. 90]

Boqueirão gi|503099779|ref|WP_013334576.1| 1 non-ribosomal peptide synthetase [Cyanothece sp. PCC 7822]

Boqueirão gi|515878079|ref|WP_017308662.1| 1 non-ribosomal peptide synthetase [Fischerella sp. PCC 9339]

Boqueirão gi|653154928|ref|WP_027403945.1| 1 non-ribosomal peptide synthetase [Aphanizomenon flos-aquae]

Boqueirão gi|653154928|ref|WP_027403945.1| 1 non-ribosomal peptide synthetase [Aphanizomenon flos-aquae]

Boqueirão gi|504891874|ref|WP_015078976.1| 1 non-ribosomal peptide synthetase [Anabaena sp. 90]

Boqueirão gi|504891874|ref|WP_015078976.1| 1 non-ribosomal peptide synthetase [Anabaena sp. 90]

Boqueirão gi|493211327|ref|WP_006196339.1| 1 non-ribosomal peptide synthetase [Nodularia spumigena]

Boqueirão gi|493211327|ref|WP_006196339.1| 1 non-ribosomal peptide synthetase [Nodularia spumigena]

Boqueirão gi|1028121175|ref|WP_063874472.1| 1 non-ribosomal peptide synthetase [Nodularia spumigena]

Boqueirão gi|1028121175|ref|WP_063874472.1| 1 non-ribosomal peptide synthetase [Nodularia spumigena]

Boqueirão gi|1121324739|ref|WP_073634534.1| 1 non-ribosomal peptide synthetase [Scytonema sp. HK-05]

Boqueirão gi|1121324739|ref|WP_073634534.1| 1 non-ribosomal peptide synthetase [Scytonema sp. HK-05]

Boqueirão gi|504892624|ref|WP_015079726.1| 1 anabaenopeptilide synthetase ApdB [Anabaena sp. 90]

Boqueirão gi|504892624|ref|WP_015079726.1| 1 anabaenopeptilide synthetase ApdB [Anabaena sp. 90]

Boqueirão gi|504892624|ref|WP_015079726.1| 1 anabaenopeptilide synthetase ApdB [Anabaena sp. 90]

Boqueirão gi|504892624|ref|WP_015079726.1| 1 anabaenopeptilide synthetase ApdB [Anabaena sp. 90]

Boqueirão gi|516352623|ref|WP_017742656.1| 1 non-ribosomal peptide synthetase [Scytonema hofmannii]

Boqueirão gi|516352623|ref|WP_017742656.1| 1 non-ribosomal peptide synthetase [Scytonema hofmannii]

Boqueirão gi|516352623|ref|WP_017742656.1| 1 non-ribosomal peptide synthetase [Scytonema hofmannii]

Boqueirão gi|516352623|ref|WP_017742656.1| 1 non-ribosomal peptide synthetase [Scytonema hofmannii]

Boqueirão gi|652389981|ref|WP_026785829.1| 1 McnC protein [Planktothrix rubescens]

Boqueirão gi|652389981|ref|WP_026785829.1| 1 McnC protein [Planktothrix rubescens]

Boqueirão gi|652389981|ref|WP_026785829.1| 1 McnC protein [Planktothrix rubescens]

Boqueirão gi|652389981|ref|WP_026785829.1| 1 McnC protein [Planktothrix rubescens]

Boqueirão gi|754792464|ref|WP_042156042.1| 1 McnC protein [Planktothrix agardhii]

Boqueirão gi|754792464|ref|WP_042156042.1| 1 McnC protein [Planktothrix agardhii]

Boqueirão gi|754792464|ref|WP_042156042.1| 1 McnC protein [Planktothrix agardhii]

Boqueirão gi|754792464|ref|WP_042156042.1| 1 McnC protein [Planktothrix agardhii]

Boqueirão gi|653003249|ref|WP_027255435.1| 1 McnC protein [Planktothrix agardhii]

Boqueirão gi|653003249|ref|WP_027255435.1| 1 McnC protein [Planktothrix agardhii]

Boqueirão gi|653003249|ref|WP_027255435.1| 1 McnC protein [Planktothrix agardhii]

Boqueirão gi|653003249|ref|WP_027255435.1| 1 McnC protein [Planktothrix agardhii]

Boqueirão gi|1028121175|ref|WP_063874472.1| 1 non-ribosomal peptide synthetase [Nodularia spumigena]

Boqueirão gi|493211327|ref|WP_006196339.1| 1 non-ribosomal peptide synthetase [Nodularia spumigena]

Boqueirão gi|653154928|ref|WP_027403945.1| 1 non-ribosomal peptide synthetase [Aphanizomenon flos-aquae]

Boqueirão gi|1121332340|ref|WP_073640239.1| 1 non-ribosomal peptide synthetase [Nostoc calcicola]

Boqueirão gi|1121324739|ref|WP_073634534.1| 1 non-ribosomal peptide synthetase [Scytonema sp. HK-05]

Boqueirão gi|916817351|ref|WP_051424407.1| 1 hypothetical protein [Aphanizomenon flos-aquae]

Boqueirão gi|652996399|ref|WP_027248866.1| 1 non-ribosomal peptide synthetase [Planktothrix agardhii]

Boqueirão gi|488838362|ref|WP_002750768.1| 1 non-ribosomal peptide synthetase [Microcystis aeruginosa]

Boqueirão gi|1054850717|ref|WP_066612889.1| 1 non-ribosomal peptide synthetase [Scytonema hofmannii]

Boqueirão gi|488864799|ref|WP_002777038.1| 1 non-ribosomal peptide synthetase [Microcystis aeruginosa]

Boqueirão gi|1028121172|ref|WP_063874469.1| 1 non-ribosomal peptide synthetase [Nodularia spumigena]

Boqueirão gi|516352606|ref|WP_017742639.1| 1 non-ribosomal peptide synthetase [Scytonema hofmannii]

Boqueirão gi|504891877|ref|WP_015078979.1| 1 non-ribosomal peptide synthetase [Anabaena sp. 90]

Boqueirão gi|1121324733|ref|WP_073634530.1| 1 non-ribosomal peptide synthetase [Scytonema sp. HK-05]

Boqueirão gi|653154931|ref|WP_027403948.1| 1 non-ribosomal peptide synthetase [Aphanizomenon flos-aquae]

Boqueirão gi|1028121174|ref|WP_063874471.1| 1 non-ribosomal peptide synthetase [Nodularia spumigena]

Boqueirão gi|653154929|ref|WP_027403946.1| 1 non-ribosomal peptide synthetase [Aphanizomenon flos-aquae]

Boqueirão gi|501377450|ref|WP_012409016.1| 1 non-ribosomal peptide synthetase [Nostoc punctiforme]

Boqueirão gi|1121324737|ref|WP_073634533.1| 1 non-ribosomal peptide synthetase [Scytonema sp. HK-05]

Boqueirão gi|504891875|ref|WP_015078977.1| 1 non-ribosomal peptide synthetase [Anabaena sp. 90]

Boqueirão gi|916817351|ref|WP_051424407.1| 1 hypothetical protein [Aphanizomenon flos-aquae]

Boqueirão gi|501377217|ref|WP_012408783.1| 1 non-ribosomal peptide synthetase [Nostoc punctiforme]

Boqueirão gi|501377217|ref|WP_012408783.1| 1 non-ribosomal peptide synthetase [Nostoc punctiforme]

Boqueirão gi|501377217|ref|WP_012408783.1| 1 non-ribosomal peptide synthetase [Nostoc punctiforme]

Boqueirão gi|501377217|ref|WP_012408783.1| 1 non-ribosomal peptide synthetase [Nostoc punctiforme]

Boqueirão gi|488864799|ref|WP_002777038.1| 1 non-ribosomal peptide synthetase [Microcystis aeruginosa]

Boqueirão gi|1054850717|ref|WP_066612889.1| 1 non-ribosomal peptide synthetase [Scytonema hofmannii]

Boqueirão gi|1054850717|ref|WP_066612889.1| 1 non-ribosomal peptide synthetase [Scytonema hofmannii]

Boqueirão gi|1054850717|ref|WP_066612889.1| 1 non-ribosomal peptide synthetase [Scytonema hofmannii]

Boqueirão gi|501225155|ref|WP_012268173.1| 1 McnA protein [Microcystis aeruginosa]

Boqueirão gi|1028121172|ref|WP_063874469.1| 1 non-ribosomal peptide synthetase [Nodularia spumigena]

Boqueirão gi|1121324733|ref|WP_073634530.1| 1 non-ribosomal peptide synthetase [Scytonema sp. HK-05]

Boqueirão gi|653154931|ref|WP_027403948.1| 1 non-ribosomal peptide synthetase [Aphanizomenon flos-aquae]

Boqueirão gi|504891877|ref|WP_015078979.1| 1 non-ribosomal peptide synthetase [Anabaena sp. 90]

Boqueirão gi|1121332335|ref|WP_073640234.1| 1 non-ribosomal peptide synthetase [Nostoc calcicola]

Boqueirão gi|493211327|ref|WP_006196339.1| 1 non-ribosomal peptide synthetase [Nodularia spumigena]

Boqueirão gi|653154928|ref|WP_027403945.1| 1 non-ribosomal peptide synthetase [Aphanizomenon flos-aquae]

Boqueirão gi|1028121175|ref|WP_063874472.1| 1 non-ribosomal peptide synthetase [Nodularia spumigena]

Boqueirão gi|1028121173|ref|WP_063874470.1| 1 non-ribosomal peptide synthetase [Nodularia spumigena]

Boqueirão gi|1028121173|ref|WP_063874470.1| 1 non-ribosomal peptide synthetase [Nodularia spumigena]

Boqueirão gi|653154930|ref|WP_027403947.1| 1 non-ribosomal peptide synthetase [Aphanizomenon flos-aquae]

Boqueirão gi|653154930|ref|WP_027403947.1| 1 non-ribosomal peptide synthetase [Aphanizomenon flos-aquae]

Boqueirão gi|504891876|ref|WP_015078978.1| 1 non-ribosomal peptide synthetase [Anabaena sp. 90]

Boqueirão gi|504891876|ref|WP_015078978.1| 1 non-ribosomal peptide synthetase [Anabaena sp. 90]

Boqueirão gi|516352607|ref|WP_017742640.1| 1 non-ribosomal peptide synthetase [Scytonema hofmannii]

Boqueirão gi|516352607|ref|WP_017742640.1| 1 non-ribosomal peptide synthetase [Scytonema hofmannii]

Boqueirão gi|493211329|ref|WP_006196341.1| 1 non-ribosomal peptide synthetase [Nodularia spumigena]

Boqueirão gi|493211329|ref|WP_006196341.1| 1 non-ribosomal peptide synthetase [Nodularia spumigena]

Boqueirão gi|916817351|ref|WP_051424407.1| 1 hypothetical protein [Aphanizomenon flos-aquae]

Boqueirão gi|488838362|ref|WP_002750768.1| 1 non-ribosomal peptide synthetase [Microcystis aeruginosa]

Boqueirão gi|640537446|ref|WP_024970454.1| 1 McnA protein [Microcystis aeruginosa]

Boqueirão gi|652996399|ref|WP_027248866.1| 1 non-ribosomal peptide synthetase [Planktothrix agardhii]

Boqueirão gi|488879468|ref|WP_002791693.1| 1 non-ribosomal peptide synthetase [Microcystis aeruginosa]

Boqueirão gi|653154928|ref|WP_027403945.1| 1 non-ribosomal peptide synthetase [Aphanizomenon flos-aquae]

Boqueirão gi|493211327|ref|WP_006196339.1| 1 non-ribosomal peptide synthetase [Nodularia spumigena]

Boqueirão gi|1028121175|ref|WP_063874472.1| 1 non-ribosomal peptide synthetase [Nodularia spumigena]

Boqueirão gi|1121324739|ref|WP_073634534.1| 1 non-ribosomal peptide synthetase [Scytonema sp. HK-05]

Boqueirão gi|1121332340|ref|WP_073640239.1| 1 non-ribosomal peptide synthetase [Nostoc calcicola]

Boqueirão gi|516352615|ref|WP_017742648.1| 1 non-ribosomal peptide synthetase [Scytonema hofmannii]

Boqueirão gi|754792464|ref|WP_042156042.1| 1 McnC protein [Planktothrix agardhii]

Boqueirão gi|653003249|ref|WP_027255435.1| 1 McnC protein [Planktothrix agardhii]

Boqueirão gi|652402732|ref|WP_026798523.1| 1 McnC protein [Planktothrix prolifica]

Boqueirão gi|652997906|ref|WP_027250316.1| 1 McnC protein [Planktothrix agardhii]

Boqueirão gi|501377217|ref|WP_012408783.1| 1 non-ribosomal peptide synthetase [Nostoc punctiforme]

Boqueirão gi|501377217|ref|WP_012408783.1| 1 non-ribosomal peptide synthetase [Nostoc punctiforme]

Boqueirão gi|501377217|ref|WP_012408783.1| 1 non-ribosomal peptide synthetase [Nostoc punctiforme]

Boqueirão gi|501377217|ref|WP_012408783.1| 1 non-ribosomal peptide synthetase [Nostoc punctiforme]

Boqueirão gi|918105858|ref|WP_052331148.1| 1 non-ribosomal peptide synthetase [Planktothrix agardhii]

Boqueirão gi|916712644|ref|WP_051319735.1| 1 MULTISPECIES: non-ribosomal peptide synthetase [Planktothrix]

Boqueirão gi|501225152|ref|WP_012268170.1| 1 non-ribosomal peptide synthetase [Microcystis aeruginosa]

Boqueirão gi|488884387|ref|WP_002796612.1| 1 non-ribosomal peptide synthetase [Microcystis aeruginosa]

Boqueirão gi|488839868|ref|WP_002752274.1| 1 MicC protein [Microcystis aeruginosa]

Boqueirão gi|516352615|ref|WP_017742648.1| 1 non-ribosomal peptide synthetase [Scytonema hofmannii]

Boqueirão gi|516352615|ref|WP_017742648.1| 1 non-ribosomal peptide synthetase [Scytonema hofmannii]

Boqueirão gi|516352615|ref|WP_017742648.1| 1 non-ribosomal peptide synthetase [Scytonema hofmannii]

Boqueirão gi|516352615|ref|WP_017742648.1| 1 non-ribosomal peptide synthetase [Scytonema hofmannii]

Boqueirão gi|516352615|ref|WP_017742648.1| 1 non-ribosomal peptide synthetase [Scytonema hofmannii]

Boqueirão gi|652389981|ref|WP_026785829.1| 1 McnC protein [Planktothrix rubescens]

Boqueirão gi|652389981|ref|WP_026785829.1| 1 McnC protein [Planktothrix rubescens]

Boqueirão gi|652389981|ref|WP_026785829.1| 1 McnC protein [Planktothrix rubescens]

Boqueirão gi|652389981|ref|WP_026785829.1| 1 McnC protein [Planktothrix rubescens]

Boqueirão gi|1094114794|ref|WP_071104293.1| 1 hypothetical protein [Moorea producens]

Boqueirão gi|918157632|ref|WP_052335408.1| 1 hypothetical protein [Tolypothrix sp. PCC 7601]

Boqueirão gi|1028121175|ref|WP_063874472.1| 1 non-ribosomal peptide synthetase [Nodularia spumigena]

Boqueirão gi|493211327|ref|WP_006196339.1| 1 non-ribosomal peptide synthetase [Nodularia spumigena]

Boqueirão gi|653154928|ref|WP_027403945.1| 1 non-ribosomal peptide synthetase [Aphanizomenon flos-aquae]

Boqueirão gi|1121332340|ref|WP_073640239.1| 1 non-ribosomal peptide synthetase [Nostoc calcicola]

Boqueirão gi|504891874|ref|WP_015078976.1| 1 non-ribosomal peptide synthetase [Anabaena sp. 90]

Boqueirão gi|488829433|ref|WP_002741839.1| 1 McnE protein [Microcystis aeruginosa]

Boqueirão gi|1094114944|ref|WP_071104443.1| 1 hypothetical protein [Moorea producens]

Boqueirão gi|764665630|ref|WP_044449765.1| 1 non-ribosomal peptide synthetase [Mastigocladus laminosus]

Boqueirão gi|916900950|ref|WP_051507663.1| 1 hypothetical protein [Clostridium botulinum]

Boqueirão gi|515878139|ref|WP_017308722.1| 1 non-ribosomal peptide synthetase [Fischerella sp. PCC 9339]

Boqueirão gi|495453921|ref|WP_008178615.1| 1 non-ribosomal peptide synthetase [Moorea producens]

Boqueirão gi|515878346|ref|WP_017308929.1| 1 non-ribosomal peptide synthetase [Fischerella sp. PCC 9339]

Boqueirão gi|923074279|ref|WP_053457693.1| 1 non-ribosomal peptide synthetase [Hapalosiphon sp. MRB220]

Boqueirão gi|515877499|ref|WP_017308082.1| 1 non-ribosomal peptide synthetase [Fischerella sp. PCC 9339]

Boqueirão gi|764668005|ref|WP_044450794.1| 1 peptide synthetase

Boqueirão gi|493211328|ref|WP_006196340.1| 1 non-ribosomal peptide synthetase [Nodularia spumigena]

Boqueirão gi|653154929|ref|WP_027403946.1| 1 non-ribosomal peptide synthetase [Aphanizomenon flos-aquae]

Boqueirão gi|1028121174|ref|WP_063874471.1| 1 non-ribosomal peptide synthetase [Nodularia spumigena]

Boqueirão gi|504891875|ref|WP_015078977.1| 1 non-ribosomal peptide synthetase [Anabaena sp. 90]

Boqueirão gi|516352608|ref|WP_017742641.1| 1 non-ribosomal peptide synthetase [Scytonema hofmannii]

Boqueirão gi|504892624|ref|WP_015079726.1| 1 anabaenopeptilide synthetase ApdB [Anabaena sp. 90]

Boqueirão gi|504892624|ref|WP_015079726.1| 1 anabaenopeptilide synthetase ApdB [Anabaena sp. 90]

Boqueirão gi|504892624|ref|WP_015079726.1| 1 anabaenopeptilide synthetase ApdB [Anabaena sp. 90]

Boqueirão gi|504892624|ref|WP_015079726.1| 1 anabaenopeptilide synthetase ApdB [Anabaena sp. 90]

Boqueirão gi|516352623|ref|WP_017742656.1| 1 non-ribosomal peptide synthetase [Scytonema hofmannii]

Boqueirão gi|516352623|ref|WP_017742656.1| 1 non-ribosomal peptide synthetase [Scytonema hofmannii]

Boqueirão gi|516352623|ref|WP_017742656.1| 1 non-ribosomal peptide synthetase [Scytonema hofmannii]

Boqueirão gi|516352623|ref|WP_017742656.1| 1 non-ribosomal peptide synthetase [Scytonema hofmannii]

Boqueirão gi|652389981|ref|WP_026785829.1| 1 McnC protein [Planktothrix rubescens]

Boqueirão gi|652389981|ref|WP_026785829.1| 1 McnC protein [Planktothrix rubescens]

Boqueirão gi|652389981|ref|WP_026785829.1| 1 McnC protein [Planktothrix rubescens]

Boqueirão gi|652389981|ref|WP_026785829.1| 1 McnC protein [Planktothrix rubescens]

Boqueirão gi|754792464|ref|WP_042156042.1| 1 McnC protein [Planktothrix agardhii]

Boqueirão gi|754792464|ref|WP_042156042.1| 1 McnC protein [Planktothrix agardhii]

Boqueirão gi|754792464|ref|WP_042156042.1| 1 McnC protein [Planktothrix agardhii]

Boqueirão gi|754792464|ref|WP_042156042.1| 1 McnC protein [Planktothrix agardhii]

Boqueirão gi|653003249|ref|WP_027255435.1| 1 McnC protein [Planktothrix agardhii]

Boqueirão gi|653003249|ref|WP_027255435.1| 1 McnC protein [Planktothrix agardhii]

Boqueirão gi|653003249|ref|WP_027255435.1| 1 McnC protein [Planktothrix agardhii]

Boqueirão gi|653003249|ref|WP_027255435.1| 1 McnC protein [Planktothrix agardhii]

Boqueirão gi|972339078|ref|WP_058995148.1| 1 non-ribosomal peptide synthetase [Leptolyngbya sp. NIES-2104]

Boqueirão gi|516352623|ref|WP_017742656.1| 1 non-ribosomal peptide synthetase [Scytonema hofmannii]

Boqueirão gi|516352623|ref|WP_017742656.1| 1 non-ribosomal peptide synthetase [Scytonema hofmannii]

Boqueirão gi|516352623|ref|WP_017742656.1| 1 non-ribosomal peptide synthetase [Scytonema hofmannii]

Boqueirão gi|516352623|ref|WP_017742656.1| 1 non-ribosomal peptide synthetase [Scytonema hofmannii]

Boqueirão gi|504892624|ref|WP_015079726.1| 1 anabaenopeptilide synthetase ApdB [Anabaena sp. 90]

Boqueirão gi|504892624|ref|WP_015079726.1| 1 anabaenopeptilide synthetase ApdB [Anabaena sp. 90]

Boqueirão gi|504892624|ref|WP_015079726.1| 1 anabaenopeptilide synthetase ApdB [Anabaena sp. 90]

Boqueirão gi|504892624|ref|WP_015079726.1| 1 anabaenopeptilide synthetase ApdB [Anabaena sp. 90]

Boqueirão gi|1056313851|ref|WP_067768741.1| 1 hypothetical protein [Nostoc sp. NIES-3756]

Boqueirão gi|1056313851|ref|WP_067768741.1| 1 hypothetical protein [Nostoc sp. NIES-3756]

Boqueirão gi|501377220|ref|WP_012408786.1| 1 non-ribosomal peptide synthetase [Nostoc punctiforme]

Boqueirão gi|501377220|ref|WP_012408786.1| 1 non-ribosomal peptide synthetase [Nostoc punctiforme]

Boqueirão gi|1011379802|ref|WP_062292567.1| 1 non-ribosomal peptide synthetase [Nostoc piscinale]

Boqueirão gi|1011379802|ref|WP_062292567.1| 1 non-ribosomal peptide synthetase [Nostoc piscinale]

Boqueirão gi|501377219|ref|WP_012408785.1| 1 non-ribosomal peptide synthetase [Nostoc punctiforme]

Boqueirão gi|1028121179|ref|WP_063874476.1| 1 non-ribosomal peptide synthetase [Nodularia spumigena]

Boqueirão gi|493215585|ref|WP_006198652.1| 1 non-ribosomal peptide synthetase [Nodularia spumigena]

Boqueirão gi|504892624|ref|WP_015079726.1| 1 anabaenopeptilide synthetase ApdB [Anabaena sp. 90]

Boqueirão gi|653154922|ref|WP_027403939.1| 1 non-ribosomal peptide synthetase [Aphanizomenon flos-aquae]

Boqueirão gi|653154922|ref|WP_027403939.1| 1 non-ribosomal peptide synthetase [Aphanizomenon flos-aquae]

Boqueirão gi|653154928|ref|WP_027403945.1| 1 non-ribosomal peptide synthetase [Aphanizomenon flos-aquae]

Boqueirão gi|493211327|ref|WP_006196339.1| 1 non-ribosomal peptide synthetase [Nodularia spumigena]

Boqueirão gi|1028121175|ref|WP_063874472.1| 1 non-ribosomal peptide synthetase [Nodularia spumigena]

Boqueirão gi|1121324739|ref|WP_073634534.1| 1 non-ribosomal peptide synthetase [Scytonema sp. HK-05]

Boqueirão gi|504891874|ref|WP_015078976.1| 1 non-ribosomal peptide synthetase [Anabaena sp. 90]

Boqueirão gi|516352615|ref|WP_017742648.1| 1 non-ribosomal peptide synthetase [Scytonema hofmannii]

Boqueirão gi|516352615|ref|WP_017742648.1| 1 non-ribosomal peptide synthetase [Scytonema hofmannii]

Boqueirão gi|501225153|ref|WP_012268171.1| 1 McnC protein [Microcystis aeruginosa]

Boqueirão gi|501225153|ref|WP_012268171.1| 1 McnC protein [Microcystis aeruginosa]

Boqueirão gi|1011379797|ref|WP_062292562.1| 1 non-ribosomal peptide synthetase [Nostoc piscinale]

Boqueirão gi|652402429|ref|WP_026798225.1| 1 McnC protein [Planktothrix prolifica]

Boqueirão gi|916599636|ref|WP_051206727.1| 1 non-ribosomal peptide synthetase [Fischerella sp. PCC 9431]

Boqueirão gi|516352615|ref|WP_017742648.1| 1 non-ribosomal peptide synthetase [Scytonema hofmannii]

Boqueirão gi|516352615|ref|WP_017742648.1| 1 non-ribosomal peptide synthetase [Scytonema hofmannii]

Boqueirão gi|516352615|ref|WP_017742648.1| 1 non-ribosomal peptide synthetase [Scytonema hofmannii]

Boqueirão gi|516352615|ref|WP_017742648.1| 1 non-ribosomal peptide synthetase [Scytonema hofmannii]

Boqueirão gi|516352623|ref|WP_017742656.1| 1 non-ribosomal peptide synthetase [Scytonema hofmannii]

Boqueirão gi|516352623|ref|WP_017742656.1| 1 non-ribosomal peptide synthetase [Scytonema hofmannii]

Boqueirão gi|516352623|ref|WP_017742656.1| 1 non-ribosomal peptide synthetase [Scytonema hofmannii]

Boqueirão gi|516352623|ref|WP_017742656.1| 1 non-ribosomal peptide synthetase [Scytonema hofmannii]

Boqueirão gi|504892624|ref|WP_015079726.1| 1 anabaenopeptilide synthetase ApdB [Anabaena sp. 90]

Boqueirão gi|504892624|ref|WP_015079726.1| 1 anabaenopeptilide synthetase ApdB [Anabaena sp. 90]

Boqueirão gi|504892624|ref|WP_015079726.1| 1 anabaenopeptilide synthetase ApdB [Anabaena sp. 90]

Boqueirão gi|504892624|ref|WP_015079726.1| 1 anabaenopeptilide synthetase ApdB [Anabaena sp. 90]

Boqueirão gi|1121324726|ref|WP_073634526.1| 1 tubC protein [Scytonema sp. HK-05]

Boqueirão gi|1121324726|ref|WP_073634526.1| 1 tubC protein [Scytonema sp. HK-05]

Boqueirão gi|817700114|ref|WP_046662633.1| 1 McnC protein [Microcystis aeruginosa]

Boqueirão gi|817700114|ref|WP_046662633.1| 1 McnC protein [Microcystis aeruginosa]

Boqueirão gi|817700114|ref|WP_046662633.1| 1 McnC protein [Microcystis aeruginosa]

Boqueirão gi|1028121173|ref|WP_063874470.1| 1 non-ribosomal peptide synthetase [Nodularia spumigena]

Boqueirão gi|1028121173|ref|WP_063874470.1| 1 non-ribosomal peptide synthetase [Nodularia spumigena]

Boqueirão gi|653154930|ref|WP_027403947.1| 1 non-ribosomal peptide synthetase [Aphanizomenon flos-aquae]

Boqueirão gi|653154930|ref|WP_027403947.1| 1 non-ribosomal peptide synthetase [Aphanizomenon flos-aquae]

Boqueirão gi|1121332337|ref|WP_073640236.1| 1 non-ribosomal peptide synthetase [Nostoc calcicola]

Boqueirão gi|1121332337|ref|WP_073640236.1| 1 non-ribosomal peptide synthetase [Nostoc calcicola]

Boqueirão gi|1060038991|ref|WP_069068160.1| 1 non-ribosomal peptide synthetase [Nostoc sp. KVJ20]

Boqueirão gi|1060038991|ref|WP_069068160.1| 1 non-ribosomal peptide synthetase [Nostoc sp. KVJ20]

Boqueirão gi|1121324736|ref|WP_073634532.1| 1 non-ribosomal peptide synthetase [Scytonema sp. HK-05]

Boqueirão gi|1121324736|ref|WP_073634532.1| 1 non-ribosomal peptide synthetase [Scytonema sp. HK-05]

Boqueirão gi|1028121172|ref|WP_063874469.1| 1 non-ribosomal peptide synthetase [Nodularia spumigena]

Boqueirão gi|493211331|ref|WP_006196342.1| 1 non-ribosomal peptide synthetase [Nodularia spumigena]

Boqueirão gi|653154931|ref|WP_027403948.1| 1 non-ribosomal peptide synthetase [Aphanizomenon flos-aquae]

Boqueirão gi|504891877|ref|WP_015078979.1| 1 non-ribosomal peptide synthetase [Anabaena sp. 90]

Boqueirão gi|501377452|ref|WP_012409018.1| 1 non-ribosomal peptide synthetase [Nostoc punctiforme]

Boqueirão gi|516352615|ref|WP_017742648.1| 1 non-ribosomal peptide synthetase [Scytonema hofmannii]

Boqueirão gi|516352615|ref|WP_017742648.1| 1 non-ribosomal peptide synthetase [Scytonema hofmannii]

Boqueirão gi|516352615|ref|WP_017742648.1| 1 non-ribosomal peptide synthetase [Scytonema hofmannii]

Boqueirão gi|516352615|ref|WP_017742648.1| 1 non-ribosomal peptide synthetase [Scytonema hofmannii]

Boqueirão gi|504892624|ref|WP_015079726.1| 1 anabaenopeptilide synthetase ApdB [Anabaena sp. 90]

Boqueirão gi|504892624|ref|WP_015079726.1| 1 anabaenopeptilide synthetase ApdB [Anabaena sp. 90]

Boqueirão gi|504892624|ref|WP_015079726.1| 1 anabaenopeptilide synthetase ApdB [Anabaena sp. 90]

Boqueirão gi|504892624|ref|WP_015079726.1| 1 anabaenopeptilide synthetase ApdB [Anabaena sp. 90]

Boqueirão gi|516352623|ref|WP_017742656.1| 1 non-ribosomal peptide synthetase [Scytonema hofmannii]

Boqueirão gi|516352623|ref|WP_017742656.1| 1 non-ribosomal peptide synthetase [Scytonema hofmannii]

Boqueirão gi|516352623|ref|WP_017742656.1| 1 non-ribosomal peptide synthetase [Scytonema hofmannii]

Boqueirão gi|516352623|ref|WP_017742656.1| 1 non-ribosomal peptide synthetase [Scytonema hofmannii]

Boqueirão gi|488864797|ref|WP_002777036.1| 1 McnC protein [Microcystis aeruginosa]

Boqueirão gi|488864797|ref|WP_002777036.1| 1 McnC protein [Microcystis aeruginosa]

Boqueirão gi|488864797|ref|WP_002777036.1| 1 McnC protein [Microcystis aeruginosa]

Boqueirão gi|488864797|ref|WP_002777036.1| 1 McnC protein [Microcystis aeruginosa]

Boqueirão gi|1121324726|ref|WP_073634526.1| 1 tubC protein [Scytonema sp. HK-05]

Boqueirão gi|1121324726|ref|WP_073634526.1| 1 tubC protein [Scytonema sp. HK-05]

Boqueirão gi|516355341|ref|WP_017745374.1| 1 hypothetical protein [Scytonema hofmannii]

Boqueirão gi|889965730|ref|WP_048868054.1| 1 AMP-dependent synthetase [Scytonema tolypothrichoides]

Boqueirão gi|515347504|ref|WP_016862272.1| 1 non-ribosomal peptide synthetase [Fischerella muscicola]

Boqueirão gi|516352623|ref|WP_017742656.1| 1 non-ribosomal peptide synthetase [Scytonema hofmannii]

Boqueirão gi|516352623|ref|WP_017742656.1| 1 non-ribosomal peptide synthetase [Scytonema hofmannii]

Boqueirão gi|516352623|ref|WP_017742656.1| 1 non-ribosomal peptide synthetase [Scytonema hofmannii]

Boqueirão gi|516352623|ref|WP_017742656.1| 1 non-ribosomal peptide synthetase [Scytonema hofmannii]

Boqueirão gi|504892624|ref|WP_015079726.1| 1 anabaenopeptilide synthetase ApdB [Anabaena sp. 90]

Boqueirão gi|504892624|ref|WP_015079726.1| 1 anabaenopeptilide synthetase ApdB [Anabaena sp. 90]

Boqueirão gi|504892624|ref|WP_015079726.1| 1 anabaenopeptilide synthetase ApdB [Anabaena sp. 90]

Boqueirão gi|504892624|ref|WP_015079726.1| 1 anabaenopeptilide synthetase ApdB [Anabaena sp. 90]

Boqueirão gi|516352615|ref|WP_017742648.1| 1 non-ribosomal peptide synthetase [Scytonema hofmannii]

Boqueirão gi|516352615|ref|WP_017742648.1| 1 non-ribosomal peptide synthetase [Scytonema hofmannii]

Boqueirão gi|516352615|ref|WP_017742648.1| 1 non-ribosomal peptide synthetase [Scytonema hofmannii]

Boqueirão gi|516352615|ref|WP_017742648.1| 1 non-ribosomal peptide synthetase [Scytonema hofmannii]

Boqueirão gi|652389981|ref|WP_026785829.1| 1 McnC protein [Planktothrix rubescens]

Boqueirão gi|652389981|ref|WP_026785829.1| 1 McnC protein [Planktothrix rubescens]

Boqueirão gi|652389981|ref|WP_026785829.1| 1 McnC protein [Planktothrix rubescens]

Boqueirão gi|652389981|ref|WP_026785829.1| 1 McnC protein [Planktothrix rubescens]

Boqueirão gi|754792464|ref|WP_042156042.1| 1 McnC protein [Planktothrix agardhii]

Boqueirão gi|754792464|ref|WP_042156042.1| 1 McnC protein [Planktothrix agardhii]

Boqueirão gi|754792464|ref|WP_042156042.1| 1 McnC protein [Planktothrix agardhii]

Boqueirão gi|754792464|ref|WP_042156042.1| 1 McnC protein [Planktothrix agardhii]

Boqueirão gi|653154922|ref|WP_027403939.1| 1 non-ribosomal peptide synthetase [Aphanizomenon flos-aquae]

Boqueirão gi|653154922|ref|WP_027403939.1| 1 non-ribosomal peptide synthetase [Aphanizomenon flos-aquae]

Boqueirão gi|516352623|ref|WP_017742656.1| 1 non-ribosomal peptide synthetase [Scytonema hofmannii]

Boqueirão gi|516352623|ref|WP_017742656.1| 1 non-ribosomal peptide synthetase [Scytonema hofmannii]

Boqueirão gi|516352623|ref|WP_017742656.1| 1 non-ribosomal peptide synthetase [Scytonema hofmannii]

Boqueirão gi|516352623|ref|WP_017742656.1| 1 non-ribosomal peptide synthetase [Scytonema hofmannii]

Boqueirão gi|1028121179|ref|WP_063874476.1| 1 non-ribosomal peptide synthetase [Nodularia spumigena]

Boqueirão gi|1028121179|ref|WP_063874476.1| 1 non-ribosomal peptide synthetase [Nodularia spumigena]

Boqueirão gi|1054850725|ref|WP_066612897.1| 1 hypothetical protein

Boqueirão gi|1054850725|ref|WP_066612897.1| 1 hypothetical protein

Boqueirão gi|1054850725|ref|WP_066612897.1| 1 hypothetical protein

Boqueirão gi|504892625|ref|WP_015079727.1| 1 anabaenopeptilide synthetase ApdA [Anabaena sp. 90]

Boqueirão gi|505043850|ref|WP_015230952.1| 1 non-ribosomal peptide synthase [Dactylococcopsis salina]

Boqueirão gi|506437201|ref|WP_015956918.1| 1 non-ribosomal peptide synthase [Cyanothece sp. PCC 7424]

Boqueirão gi|825994982|ref|WP_047156157.1| 1 amino acid adenylation protein

Boqueirão gi|499931748|ref|WP_011612482.1| 1 non-ribosomal peptide synthetase [Trichodesmium erythraeum]

Boqueirão gi|1084329345|ref|WP_070393404.1| 1 non-ribosomal peptide synthetase [Moorea producens]

Boqueirão gi|653154931|ref|WP_027403948.1| 1 non-ribosomal peptide synthetase [Aphanizomenon flos-aquae]

Boqueirão gi|1028121172|ref|WP_063874469.1| 1 non-ribosomal peptide synthetase [Nodularia spumigena]

Boqueirão gi|504891877|ref|WP_015078979.1| 1 non-ribosomal peptide synthetase [Anabaena sp. 90]

Boqueirão gi|493211331|ref|WP_006196342.1| 1 non-ribosomal peptide synthetase [Nodularia spumigena]

Boqueirão gi|501377452|ref|WP_012409018.1| 1 non-ribosomal peptide synthetase [Nostoc punctiforme]

Boqueirão gi|493211329|ref|WP_006196341.1| 1 non-ribosomal peptide synthetase [Nodularia spumigena]

Boqueirão gi|493211329|ref|WP_006196341.1| 1 non-ribosomal peptide synthetase [Nodularia spumigena]

Boqueirão gi|501377451|ref|WP_012409017.1| 1 non-ribosomal peptide synthetase [Nostoc punctiforme]

Boqueirão gi|501377451|ref|WP_012409017.1| 1 non-ribosomal peptide synthetase [Nostoc punctiforme]

Boqueirão gi|653154930|ref|WP_027403947.1| 1 non-ribosomal peptide synthetase [Aphanizomenon flos-aquae]

Boqueirão gi|653154930|ref|WP_027403947.1| 1 non-ribosomal peptide synthetase [Aphanizomenon flos-aquae]

Boqueirão gi|1121332337|ref|WP_073640236.1| 1 non-ribosomal peptide synthetase [Nostoc calcicola]

Boqueirão gi|1121332337|ref|WP_073640236.1| 1 non-ribosomal peptide synthetase [Nostoc calcicola]

Boqueirão gi|652996393|ref|WP_027248860.1| 1 non-ribosomal peptide synthetase [Planktothrix agardhii]

Boqueirão gi|652996393|ref|WP_027248860.1| 1 non-ribosomal peptide synthetase [Planktothrix agardhii]

Boqueirão gi|504891876|ref|WP_015078978.1| 1 non-ribosomal peptide synthetase [Anabaena sp. 90]

Boqueirão gi|1028121173|ref|WP_063874470.1| 1 non-ribosomal peptide synthetase [Nodularia spumigena]

Boqueirão gi|493211329|ref|WP_006196341.1| 1 non-ribosomal peptide synthetase [Nodularia spumigena]

Boqueirão gi|493211329|ref|WP_006196341.1| 1 non-ribosomal peptide synthetase [Nodularia spumigena]

Boqueirão gi|653154930|ref|WP_027403947.1| 1 non-ribosomal peptide synthetase [Aphanizomenon flos-aquae]

Boqueirão gi|493215585|ref|WP_006198652.1| 1 non-ribosomal peptide synthetase [Nodularia spumigena]

Boqueirão gi|1028121172|ref|WP_063874469.1| 1 non-ribosomal peptide synthetase [Nodularia spumigena]

Boqueirão gi|653154931|ref|WP_027403948.1| 1 non-ribosomal peptide synthetase [Aphanizomenon flos-aquae]

Boqueirão gi|504891877|ref|WP_015078979.1| 1 non-ribosomal peptide synthetase [Anabaena sp. 90]

Boqueirão gi|1060038995|ref|WP_069068162.1| 1 non-ribosomal peptide synthetase [Nostoc sp. KVJ20]

Boqueirão gi|493211331|ref|WP_006196342.1| 1 non-ribosomal peptide synthetase [Nodularia spumigena]

Boqueirão gi|653154930|ref|WP_027403947.1| 1 non-ribosomal peptide synthetase [Aphanizomenon flos-aquae]

Boqueirão gi|653154930|ref|WP_027403947.1| 1 non-ribosomal peptide synthetase [Aphanizomenon flos-aquae]

Boqueirão gi|493211329|ref|WP_006196341.1| 1 non-ribosomal peptide synthetase [Nodularia spumigena]

Boqueirão gi|493211329|ref|WP_006196341.1| 1 non-ribosomal peptide synthetase [Nodularia spumigena]

Boqueirão gi|504891876|ref|WP_015078978.1| 1 non-ribosomal peptide synthetase [Anabaena sp. 90]

Boqueirão gi|504891876|ref|WP_015078978.1| 1 non-ribosomal peptide synthetase [Anabaena sp. 90]

Boqueirão gi|1028121173|ref|WP_063874470.1| 1 non-ribosomal peptide synthetase [Nodularia spumigena]

Boqueirão gi|1028121173|ref|WP_063874470.1| 1 non-ribosomal peptide synthetase [Nodularia spumigena]

Boqueirão gi|1121332337|ref|WP_073640236.1| 1 non-ribosomal peptide synthetase [Nostoc calcicola]

Boqueirão gi|1121332337|ref|WP_073640236.1| 1 non-ribosomal peptide synthetase [Nostoc calcicola]

Boqueirão gi|516352615|ref|WP_017742648.1| 1 non-ribosomal peptide synthetase [Scytonema hofmannii]

Boqueirão gi|516352615|ref|WP_017742648.1| 1 non-ribosomal peptide synthetase [Scytonema hofmannii]

Boqueirão gi|516352615|ref|WP_017742648.1| 1 non-ribosomal peptide synthetase [Scytonema hofmannii]

Boqueirão gi|516352615|ref|WP_017742648.1| 1 non-ribosomal peptide synthetase [Scytonema hofmannii]

Boqueirão gi|1002985852|ref|WP_061430379.1| 1 non-ribosomal peptide synthetase [Microcystis aeruginosa]

Boqueirão gi|1002985852|ref|WP_061430379.1| 1 non-ribosomal peptide synthetase [Microcystis aeruginosa]

Boqueirão gi|1002985852|ref|WP_061430379.1| 1 non-ribosomal peptide synthetase [Microcystis aeruginosa]

Boqueirão gi|1002985852|ref|WP_061430379.1| 1 non-ribosomal peptide synthetase [Microcystis aeruginosa]

Boqueirão gi|488864797|ref|WP_002777036.1| 1 McnC protein [Microcystis aeruginosa]

Boqueirão gi|488864797|ref|WP_002777036.1| 1 McnC protein [Microcystis aeruginosa]

Boqueirão gi|488864797|ref|WP_002777036.1| 1 McnC protein [Microcystis aeruginosa]

Boqueirão gi|488864797|ref|WP_002777036.1| 1 McnC protein [Microcystis aeruginosa]

Boqueirão gi|817700114|ref|WP_046662633.1| 1 McnC protein [Microcystis aeruginosa]

Boqueirão gi|817700114|ref|WP_046662633.1| 1 McnC protein [Microcystis aeruginosa]

Boqueirão gi|817700114|ref|WP_046662633.1| 1 McnC protein [Microcystis aeruginosa]

Boqueirão gi|817700114|ref|WP_046662633.1| 1 McnC protein [Microcystis aeruginosa]

Boqueirão gi|488829431|ref|WP_002741837.1| 1 McnC protein [Microcystis aeruginosa]

Boqueirão gi|488829431|ref|WP_002741837.1| 1 McnC protein [Microcystis aeruginosa]

Boqueirão gi|488829431|ref|WP_002741837.1| 1 McnC protein [Microcystis aeruginosa]

Boqueirão gi|488829431|ref|WP_002741837.1| 1 McnC protein [Microcystis aeruginosa]

Boqueirão gi|493211328|ref|WP_006196340.1| 1 non-ribosomal peptide synthetase [Nodularia spumigena]

Boqueirão gi|653154929|ref|WP_027403946.1| 1 non-ribosomal peptide synthetase [Aphanizomenon flos-aquae]

Boqueirão gi|1028121174|ref|WP_063874471.1| 1 non-ribosomal peptide synthetase [Nodularia spumigena]

Boqueirão gi|1060038990|ref|WP_069068159.1| 1 non-ribosomal peptide synthetase [Nostoc sp. KVJ20]

Boqueirão gi|1121324737|ref|WP_073634533.1| 1 non-ribosomal peptide synthetase [Scytonema sp. HK-05]

Boqueirão gi|1056313851|ref|WP_067768741.1| 1 hypothetical protein [Nostoc sp. NIES-3756]

Boqueirão gi|1011379802|ref|WP_062292567.1| 1 non-ribosomal peptide synthetase [Nostoc piscinale]

Boqueirão gi|1011379797|ref|WP_062292562.1| 1 non-ribosomal peptide synthetase [Nostoc piscinale]

Boqueirão gi|501377220|ref|WP_012408786.1| 1 non-ribosomal peptide synthetase [Nostoc punctiforme]

Boqueirão gi|1060047544|ref|WP_069074215.1| 1 non-ribosomal peptide synthetase [Nostoc sp. KVJ20]

Boqueirão gi|1121335212|ref|WP_073643111.1| 1 non-ribosomal peptide synthetase [Nostoc calcicola]

Boqueirão gi|1121335212|ref|WP_073643111.1| 1 non-ribosomal peptide synthetase [Nostoc calcicola]

Boqueirão gi|1062970892|ref|WP_069351502.1| 1 non-ribosomal peptide synthetase

Boqueirão gi|504209638|ref|WP_014396740.1| 1 non-ribosomal peptide synthetase [Corallococcus coralloides]

Boqueirão gi|504209638|ref|WP_014396740.1| 1 non-ribosomal peptide synthetase [Corallococcus coralloides]

Boqueirão gi|504209638|ref|WP_014396740.1| 1 non-ribosomal peptide synthetase [Corallococcus coralloides]

Boqueirão gi|504209638|ref|WP_014396740.1| 1 non-ribosomal peptide synthetase [Corallococcus coralloides]

Boqueirão gi|504209638|ref|WP_014396740.1| 1 non-ribosomal peptide synthetase [Corallococcus coralloides]

Boqueirão gi|504209638|ref|WP_014396740.1| 1 non-ribosomal peptide synthetase [Corallococcus coralloides]

Boqueirão gi|504209638|ref|WP_014396740.1| 1 non-ribosomal peptide synthetase [Corallococcus coralloides]

Boqueirão gi|504209638|ref|WP_014396740.1| 1 non-ribosomal peptide synthetase [Corallococcus coralloides]

Boqueirão gi|504209638|ref|WP_014396740.1| 1 non-ribosomal peptide synthetase [Corallococcus coralloides]

Boqueirão gi|515861073|ref|WP_017291701.1| 1 non-ribosomal peptide synthetase [Leptolyngbya boryana]

Boqueirão gi|757157445|ref|WP_042711533.1| 1 non-ribosomal peptide synthetase

Boqueirão gi|757157445|ref|WP_042711533.1| 1 non-ribosomal peptide synthetase

Boqueirão gi|757157445|ref|WP_042711533.1| 1 non-ribosomal peptide synthetase

Boqueirão gi|757157445|ref|WP_042711533.1| 1 non-ribosomal peptide synthetase

Boqueirão gi|1028121175|ref|WP_063874472.1| 1 non-ribosomal peptide synthetase [Nodularia spumigena]

Boqueirão gi|1028121175|ref|WP_063874472.1| 1 non-ribosomal peptide synthetase [Nodularia spumigena]

Boqueirão gi|493211327|ref|WP_006196339.1| 1 non-ribosomal peptide synthetase [Nodularia spumigena]

Boqueirão gi|493211327|ref|WP_006196339.1| 1 non-ribosomal peptide synthetase [Nodularia spumigena]

Boqueirão gi|653154928|ref|WP_027403945.1| 1 non-ribosomal peptide synthetase [Aphanizomenon flos-aquae]

Boqueirão gi|653154928|ref|WP_027403945.1| 1 non-ribosomal peptide synthetase [Aphanizomenon flos-aquae]

Boqueirão gi|504891874|ref|WP_015078976.1| 1 non-ribosomal peptide synthetase [Anabaena sp. 90]

Boqueirão gi|504891874|ref|WP_015078976.1| 1 non-ribosomal peptide synthetase [Anabaena sp. 90]

Boqueirão gi|919116792|ref|WP_052672433.1| 1 non-ribosomal peptide synthetase [Aliterella atlantica]

Boqueirão gi|919116792|ref|WP_052672433.1| 1 non-ribosomal peptide synthetase [Aliterella atlantica]

Boqueirão gi|919116792|ref|WP_052672433.1| 1 non-ribosomal peptide synthetase [Aliterella atlantica]

Boqueirão gi|553733676|ref|WP_023068313.1| 1 non-ribosomal peptide synthetase [Lyngbya aestuarii]

Boqueirão gi|916817351|ref|WP_051424407.1| 1 hypothetical protein [Aphanizomenon flos-aquae]

Boqueirão gi|488838362|ref|WP_002750768.1| 1 non-ribosomal peptide synthetase [Microcystis aeruginosa]

Boqueirão gi|652996399|ref|WP_027248866.1| 1 non-ribosomal peptide synthetase [Planktothrix agardhii]

Boqueirão gi|652996399|ref|WP_027248866.1| 1 non-ribosomal peptide synthetase [Planktothrix agardhii]

Boqueirão gi|652996399|ref|WP_027248866.1| 1 non-ribosomal peptide synthetase [Planktothrix agardhii]

Boqueirão gi|501377217|ref|WP_012408783.1| 1 non-ribosomal peptide synthetase [Nostoc punctiforme]

Boqueirão gi|501377217|ref|WP_012408783.1| 1 non-ribosomal peptide synthetase [Nostoc punctiforme]

Boqueirão gi|501377217|ref|WP_012408783.1| 1 non-ribosomal peptide synthetase [Nostoc punctiforme]

Boqueirão gi|501377217|ref|WP_012408783.1| 1 non-ribosomal peptide synthetase [Nostoc punctiforme]

Boqueirão gi|640537446|ref|WP_024970454.1| 1 McnA protein [Microcystis aeruginosa]

Boqueirão gi|493211328|ref|WP_006196340.1| 1 non-ribosomal peptide synthetase [Nodularia spumigena]

Boqueirão gi|1028121174|ref|WP_063874471.1| 1 non-ribosomal peptide synthetase [Nodularia spumigena]

Boqueirão gi|653154929|ref|WP_027403946.1| 1 non-ribosomal peptide synthetase [Aphanizomenon flos-aquae]

Boqueirão gi|504891875|ref|WP_015078977.1| 1 non-ribosomal peptide synthetase [Anabaena sp. 90]

Boqueirão gi|1060038990|ref|WP_069068159.1| 1 non-ribosomal peptide synthetase [Nostoc sp. KVJ20]

Boqueirão gi|1028121172|ref|WP_063874469.1| 1 non-ribosomal peptide synthetase [Nodularia spumigena]

Boqueirão gi|493211331|ref|WP_006196342.1| 1 non-ribosomal peptide synthetase [Nodularia spumigena]

Boqueirão gi|1121324733|ref|WP_073634530.1| 1 non-ribosomal peptide synthetase [Scytonema sp. HK-05]

Boqueirão gi|1121332335|ref|WP_073640234.1| 1 non-ribosomal peptide synthetase [Nostoc calcicola]

Boqueirão gi|516352606|ref|WP_017742639.1| 1 non-ribosomal peptide synthetase [Scytonema hofmannii]

Boqueirão gi|505027968|ref|WP_015215070.1| 1 non-ribosomal peptide synthetase [Anabaena cylindrica]

Boqueirão gi|505010331|ref|WP_015197433.1| 1 non-ribosomal peptide synthetase [Calothrix parietina]

Boqueirão gi|1096253773|ref|WP_071191769.1| 1 non-ribosomal peptide synthetase [Trichormus sp. NMC-1]

Boqueirão gi|504944771|ref|WP_015131873.1| 1 non-ribosomal peptide synthetase [Calothrix sp. PCC 7507]

Boqueirão gi|516330926|ref|WP_017721585.1| 1 hypothetical protein

Boqueirão gi|1028121174|ref|WP_063874471.1| 1 non-ribosomal peptide synthetase [Nodularia spumigena]

Boqueirão gi|653154929|ref|WP_027403946.1| 1 non-ribosomal peptide synthetase [Aphanizomenon flos-aquae]

Boqueirão gi|504891875|ref|WP_015078977.1| 1 non-ribosomal peptide synthetase [Anabaena sp. 90]

Boqueirão gi|1121332339|ref|WP_073640238.1| 1 non-ribosomal peptide synthetase [Nostoc calcicola]

Boqueirão gi|1121324737|ref|WP_073634533.1| 1 non-ribosomal peptide synthetase [Scytonema sp. HK-05]

Boqueirão gi|1056326623|ref|WP_067776983.1| 1 non-ribosomal peptide synthetase [Nostoc sp. NIES-3756]

Boqueirão gi|797211261|ref|WP_045872205.1| 1 non-ribosomal peptide synthetase [Tolypothrix sp. PCC 7601]

Boqueirão gi|1096251028|ref|WP_071189200.1| 1 non-ribosomal peptide synthetase [Trichormus sp. NMC-1]

Boqueirão gi|916581065|ref|WP_051188156.1| 1 non-ribosomal peptide synthetase [Brevibacillus thermoruber]

Boqueirão gi|515383321|ref|WP_016876680.1| 1 non-ribosomal peptide synthetase [Chlorogloeopsis fritschii]

Boqueirão gi|1028121172|ref|WP_063874469.1| 1 non-ribosomal peptide synthetase [Nodularia spumigena]

Boqueirão gi|653154931|ref|WP_027403948.1| 1 non-ribosomal peptide synthetase [Aphanizomenon flos-aquae]

Boqueirão gi|504891877|ref|WP_015078979.1| 1 non-ribosomal peptide synthetase [Anabaena sp. 90]

Boqueirão gi|493211331|ref|WP_006196342.1| 1 non-ribosomal peptide synthetase [Nodularia spumigena]

Boqueirão gi|1121324733|ref|WP_073634530.1| 1 non-ribosomal peptide synthetase [Scytonema sp. HK-05]

Boqueirão gi|499306023|ref|WP_010996798.1| 1 non-ribosomal peptide synthetase [Nostoc sp. PCC 7120]

Boqueirão gi|1028119247|ref|WP_063872544.1| 1 non-ribosomal peptide synthetase [Nodularia spumigena]

Boqueirão gi|493207403|ref|WP_006194156.1| 1 non-ribosomal peptide synthetase [Nodularia spumigena]

Boqueirão gi|493577043|ref|WP_006530164.1| 1 non-ribosomal peptide synthetase [Gloeocapsa sp. PCC 73106]

Boqueirão gi|750180047|ref|WP_040484121.1| 1 non-ribosomal peptide synthetase [Lyngbya aestuarii]

Boqueirão gi|653154931|ref|WP_027403948.1| 1 non-ribosomal peptide synthetase [Aphanizomenon flos-aquae]

Boqueirão gi|1028121172|ref|WP_063874469.1| 1 non-ribosomal peptide synthetase [Nodularia spumigena]

Boqueirão gi|493211329|ref|WP_006196341.1| 1 non-ribosomal peptide synthetase [Nodularia spumigena]

Boqueirão gi|493211329|ref|WP_006196341.1| 1 non-ribosomal peptide synthetase [Nodularia spumigena]

Boqueirão gi|504891877|ref|WP_015078979.1| 1 non-ribosomal peptide synthetase [Anabaena sp. 90]

Boqueirão gi|504891876|ref|WP_015078978.1| 1 non-ribosomal peptide synthetase [Anabaena sp. 90]

Boqueirão gi|516352623|ref|WP_017742656.1| 1 non-ribosomal peptide synthetase [Scytonema hofmannii]

Boqueirão gi|516352615|ref|WP_017742648.1| 1 non-ribosomal peptide synthetase [Scytonema hofmannii]

Boqueirão gi|503099779|ref|WP_013334576.1| 1 non-ribosomal peptide synthetase [Cyanothece sp. PCC 7822]

Boqueirão gi|652389981|ref|WP_026785829.1| 1 McnC protein [Planktothrix rubescens]

Boqueirão gi|653003249|ref|WP_027255435.1| 1 McnC protein [Planktothrix agardhii]

Boqueirão gi|653154930|ref|WP_027403947.1| 1 non-ribosomal peptide synthetase [Aphanizomenon flos-aquae]

Boqueirão gi|653154930|ref|WP_027403947.1| 1 non-ribosomal peptide synthetase [Aphanizomenon flos-aquae]

Boqueirão gi|1028121173|ref|WP_063874470.1| 1 non-ribosomal peptide synthetase [Nodularia spumigena]

Boqueirão gi|1028121173|ref|WP_063874470.1| 1 non-ribosomal peptide synthetase [Nodularia spumigena]

Boqueirão gi|501377451|ref|WP_012409017.1| 1 non-ribosomal peptide synthetase [Nostoc punctiforme]

Boqueirão gi|501377451|ref|WP_012409017.1| 1 non-ribosomal peptide synthetase [Nostoc punctiforme]

Boqueirão gi|1121324736|ref|WP_073634532.1| 1 non-ribosomal peptide synthetase [Scytonema sp. HK-05]

Boqueirão gi|1121324736|ref|WP_073634532.1| 1 non-ribosomal peptide synthetase [Scytonema sp. HK-05]

Boqueirão gi|493211329|ref|WP_006196341.1| 1 non-ribosomal peptide synthetase [Nodularia spumigena]

Boqueirão gi|493211329|ref|WP_006196341.1| 1 non-ribosomal peptide synthetase [Nodularia spumigena]

Boqueirão gi|516352615|ref|WP_017742648.1| 1 non-ribosomal peptide synthetase [Scytonema hofmannii]

Boqueirão gi|516352615|ref|WP_017742648.1| 1 non-ribosomal peptide synthetase [Scytonema hofmannii]

Boqueirão gi|516352615|ref|WP_017742648.1| 1 non-ribosomal peptide synthetase [Scytonema hofmannii]

Boqueirão gi|516352615|ref|WP_017742648.1| 1 non-ribosomal peptide synthetase [Scytonema hofmannii]

Boqueirão gi|516352615|ref|WP_017742648.1| 1 non-ribosomal peptide synthetase [Scytonema hofmannii]

Boqueirão gi|488839868|ref|WP_002752274.1| 1 MicC protein [Microcystis aeruginosa]

Boqueirão gi|1054850722|ref|WP_066612894.1| 1 hypothetical protein [Scytonema hofmannii]

Boqueirão gi|1054850722|ref|WP_066612894.1| 1 hypothetical protein [Scytonema hofmannii]

Boqueirão gi|1054850722|ref|WP_066612894.1| 1 hypothetical protein [Scytonema hofmannii]

Boqueirão gi|516352623|ref|WP_017742656.1| 1 non-ribosomal peptide synthetase [Scytonema hofmannii]

Boqueirão gi|516352623|ref|WP_017742656.1| 1 non-ribosomal peptide synthetase [Scytonema hofmannii]

Boqueirão gi|516352623|ref|WP_017742656.1| 1 non-ribosomal peptide synthetase [Scytonema hofmannii]

Boqueirão gi|516352623|ref|WP_017742656.1| 1 non-ribosomal peptide synthetase [Scytonema hofmannii]

Boqueirão gi|504892624|ref|WP_015079726.1| 1 anabaenopeptilide synthetase ApdB [Anabaena sp. 90]

Boqueirão gi|504892624|ref|WP_015079726.1| 1 anabaenopeptilide synthetase ApdB [Anabaena sp. 90]

Boqueirão gi|504892624|ref|WP_015079726.1| 1 anabaenopeptilide synthetase ApdB [Anabaena sp. 90]

Boqueirão gi|504892624|ref|WP_015079726.1| 1 anabaenopeptilide synthetase ApdB [Anabaena sp. 90]

Boqueirão gi|516352623|ref|WP_017742656.1| 1 non-ribosomal peptide synthetase [Scytonema hofmannii]

Boqueirão gi|516352623|ref|WP_017742656.1| 1 non-ribosomal peptide synthetase [Scytonema hofmannii]

Boqueirão gi|516352623|ref|WP_017742656.1| 1 non-ribosomal peptide synthetase [Scytonema hofmannii]

Boqueirão gi|516352623|ref|WP_017742656.1| 1 non-ribosomal peptide synthetase [Scytonema hofmannii]

Boqueirão gi|504892624|ref|WP_015079726.1| 1 anabaenopeptilide synthetase ApdB [Anabaena sp. 90]

Boqueirão gi|504892624|ref|WP_015079726.1| 1 anabaenopeptilide synthetase ApdB [Anabaena sp. 90]

Boqueirão gi|504892624|ref|WP_015079726.1| 1 anabaenopeptilide synthetase ApdB [Anabaena sp. 90]

Boqueirão gi|504892624|ref|WP_015079726.1| 1 anabaenopeptilide synthetase ApdB [Anabaena sp. 90]

Boqueirão gi|501377220|ref|WP_012408786.1| 1 non-ribosomal peptide synthetase [Nostoc punctiforme]

Boqueirão gi|501377220|ref|WP_012408786.1| 1 non-ribosomal peptide synthetase [Nostoc punctiforme]

Boqueirão gi|1011379802|ref|WP_062292567.1| 1 non-ribosomal peptide synthetase [Nostoc piscinale]

Boqueirão gi|1011379802|ref|WP_062292567.1| 1 non-ribosomal peptide synthetase [Nostoc piscinale]

Boqueirão gi|640535236|ref|WP_024968868.1| 1 McnC protein [Microcystis aeruginosa]

Boqueirão gi|640535236|ref|WP_024968868.1| 1 McnC protein [Microcystis aeruginosa]

Boqueirão gi|640535236|ref|WP_024968868.1| 1 McnC protein [Microcystis aeruginosa]

Boqueirão gi|640535236|ref|WP_024968868.1| 1 McnC protein [Microcystis aeruginosa]

Boqueirão gi|1028121172|ref|WP_063874469.1| 1 non-ribosomal peptide synthetase [Nodularia spumigena]

Boqueirão gi|1121324733|ref|WP_073634530.1| 1 non-ribosomal peptide synthetase [Scytonema sp. HK-05]

Boqueirão gi|516352606|ref|WP_017742639.1| 1 non-ribosomal peptide synthetase [Scytonema hofmannii]

Boqueirão gi|493211331|ref|WP_006196342.1| 1 non-ribosomal peptide synthetase [Nodularia spumigena]

Boqueirão gi|653154931|ref|WP_027403948.1| 1 non-ribosomal peptide synthetase [Aphanizomenon flos-aquae]

Boqueirão gi|501377220|ref|WP_012408786.1| 1 non-ribosomal peptide synthetase [Nostoc punctiforme]

Boqueirão gi|501377220|ref|WP_012408786.1| 1 non-ribosomal peptide synthetase [Nostoc punctiforme]

Boqueirão gi|923072503|ref|WP_053455917.1| 1 non-ribosomal peptide synthase [Hapalosiphon sp. MRB220]

Boqueirão gi|916599636|ref|WP_051206727.1| 1 non-ribosomal peptide synthetase [Fischerella sp. PCC 9431]

Boqueirão gi|1121319237|ref|WP_073629376.1| 1 non-ribosomal peptide synthetase [Scytonema sp. HK-05]

Boqueirão gi|1121319237|ref|WP_073629376.1| 1 non-ribosomal peptide synthetase [Scytonema sp. HK-05]

Boqueirão gi|1121319237|ref|WP_073629376.1| 1 non-ribosomal peptide synthetase [Scytonema sp. HK-05]

Boqueirão gi|652399816|ref|WP_026795618.1| 1 MULTISPECIES: McnB protein [Planktothrix]

Boqueirão gi|653154928|ref|WP_027403945.1| 1 non-ribosomal peptide synthetase [Aphanizomenon flos-aquae]

Boqueirão gi|653154928|ref|WP_027403945.1| 1 non-ribosomal peptide synthetase [Aphanizomenon flos-aquae]

Boqueirão gi|1028121175|ref|WP_063874472.1| 1 non-ribosomal peptide synthetase [Nodularia spumigena]

Boqueirão gi|1028121175|ref|WP_063874472.1| 1 non-ribosomal peptide synthetase [Nodularia spumigena]

Boqueirão gi|493211327|ref|WP_006196339.1| 1 non-ribosomal peptide synthetase [Nodularia spumigena]

Boqueirão gi|493211327|ref|WP_006196339.1| 1 non-ribosomal peptide synthetase [Nodularia spumigena]

Boqueirão gi|1121324739|ref|WP_073634534.1| 1 non-ribosomal peptide synthetase [Scytonema sp. HK-05]

Boqueirão gi|1121324739|ref|WP_073634534.1| 1 non-ribosomal peptide synthetase [Scytonema sp. HK-05]

Boqueirão gi|516352609|ref|WP_017742642.1| 1 non-ribosomal peptide synthetase [Scytonema hofmannii]

Boqueirão gi|516352609|ref|WP_017742642.1| 1 non-ribosomal peptide synthetase [Scytonema hofmannii]

Boqueirão gi|653154930|ref|WP_027403947.1| 1 non-ribosomal peptide synthetase [Aphanizomenon flos-aquae]

Boqueirão gi|653154930|ref|WP_027403947.1| 1 non-ribosomal peptide synthetase [Aphanizomenon flos-aquae]

Boqueirão gi|1028121173|ref|WP_063874470.1| 1 non-ribosomal peptide synthetase [Nodularia spumigena]

Boqueirão gi|1028121173|ref|WP_063874470.1| 1 non-ribosomal peptide synthetase [Nodularia spumigena]

Boqueirão gi|1121324736|ref|WP_073634532.1| 1 non-ribosomal peptide synthetase [Scytonema sp. HK-05]

Boqueirão gi|1121324736|ref|WP_073634532.1| 1 non-ribosomal peptide synthetase [Scytonema sp. HK-05]

Boqueirão gi|501377451|ref|WP_012409017.1| 1 non-ribosomal peptide synthetase [Nostoc punctiforme]

Boqueirão gi|501377451|ref|WP_012409017.1| 1 non-ribosomal peptide synthetase [Nostoc punctiforme]

Boqueirão gi|493211329|ref|WP_006196341.1| 1 non-ribosomal peptide synthetase [Nodularia spumigena]

Boqueirão gi|493211329|ref|WP_006196341.1| 1 non-ribosomal peptide synthetase [Nodularia spumigena]

Boqueirão gi|740464744|ref|WP_038296138.1| 1 non-ribosomal peptide synthetase [[Scytonema hofmanni] UTEX B 1581]

Boqueirão gi|515878078|ref|WP_017308661.1| 1 non-ribosomal peptide synthetase [Fischerella sp. PCC 9339]

Boqueirão gi|515878078|ref|WP_017308661.1| 1 non-ribosomal peptide synthetase [Fischerella sp. PCC 9339]

Boqueirão gi|515878078|ref|WP_017308661.1| 1 non-ribosomal peptide synthetase [Fischerella sp. PCC 9339]

Boqueirão gi|488879468|ref|WP_002791693.1| 1 non-ribosomal peptide synthetase [Microcystis aeruginosa]

Boqueirão gi|490389201|ref|WP_004268656.1| 1 non-ribosomal peptide synthetase [Microcystis aeruginosa]

Boqueirão gi|916817351|ref|WP_051424407.1| 1 hypothetical protein [Aphanizomenon flos-aquae]

Boqueirão gi|763119425|ref|WP_043999339.1| 1 non-ribosomal peptide synthetase

Boqueirão gi|488864799|ref|WP_002777038.1| 1 non-ribosomal peptide synthetase [Microcystis aeruginosa]

Boqueirão gi|1114016452|ref|WP_072040811.1| 1 hypothetical protein [Tolypothrix bouteillei]

Boqueirão gi|1028121179|ref|WP_063874476.1| 1 non-ribosomal peptide synthetase [Nodularia spumigena]

Boqueirão gi|493215585|ref|WP_006198652.1| 1 non-ribosomal peptide synthetase [Nodularia spumigena]

Boqueirão gi|501377949|ref|WP_012409515.1| 1 non-ribosomal peptide synthetase [Nostoc punctiforme]

Boqueirão gi|501377949|ref|WP_012409515.1| 1 non-ribosomal peptide synthetase [Nostoc punctiforme]

Boqueirão gi|501377949|ref|WP_012409515.1| 1 non-ribosomal peptide synthetase [Nostoc punctiforme]

Boqueirão gi|640537447|ref|WP_024970455.1| 1 McnB protein

Boqueirão gi|504991557|ref|WP_015178659.1| 1 non-ribosomal peptide synthetase [Oscillatoria nigro-viridis]

Boqueirão gi|499306028|ref|WP_010996803.1| 1 non-ribosomal peptide synthase [Nostoc sp. PCC 7120]

Boqueirão gi|917354840|ref|WP_051961552.1| 1 non-ribosomal peptide synthetase [Methylobacter sp. BBA5.1]

Boqueirão gi|916410567|ref|WP_051114311.1| 1 non-ribosomal peptide synthetase [Methylobacter marinus]

Boqueirão gi|860409681|ref|WP_048415978.1| 1 non-ribosomal peptide synthetase [Chromobacterium sp. LK11]

Boqueirão gi|515359317|ref|WP_016866913.1| 1 non-ribosomal peptide synthetase [Fischerella muscicola]

Boqueirão gi|1121324807|ref|WP_073634573.1| 1 non-ribosomal peptide synthetase [Scytonema sp. HK-05]

Boqueirão gi|497068867|ref|WP_009456266.1| 1 MULTISPECIES: non-ribosomal peptide synthetase [Fischerella]

Boqueirão gi|1011331875|ref|WP_062246093.1| 1 non-ribosomal peptide synthetase [Fischerella sp. NIES-3754]

Boqueirão gi|504968597|ref|WP_015155699.1| 1 non-ribosomal peptide synthetase [Chroococcidiopsis thermalis]

Boqueirão gi|640535236|ref|WP_024968868.1| 1 McnC protein [Microcystis aeruginosa]

Boqueirão gi|640535236|ref|WP_024968868.1| 1 McnC protein [Microcystis aeruginosa]

Boqueirão gi|640535236|ref|WP_024968868.1| 1 McnC protein [Microcystis aeruginosa]

Boqueirão gi|640535236|ref|WP_024968868.1| 1 McnC protein [Microcystis aeruginosa]

Boqueirão gi|516352623|ref|WP_017742656.1| 1 non-ribosomal peptide synthetase [Scytonema hofmannii]

Boqueirão gi|516352623|ref|WP_017742656.1| 1 non-ribosomal peptide synthetase [Scytonema hofmannii]

Boqueirão gi|516352623|ref|WP_017742656.1| 1 non-ribosomal peptide synthetase [Scytonema hofmannii]

Boqueirão gi|516352623|ref|WP_017742656.1| 1 non-ribosomal peptide synthetase [Scytonema hofmannii]

Boqueirão gi|488829431|ref|WP_002741837.1| 1 McnC protein [Microcystis aeruginosa]

Boqueirão gi|488829431|ref|WP_002741837.1| 1 McnC protein [Microcystis aeruginosa]

Boqueirão gi|488829431|ref|WP_002741837.1| 1 McnC protein [Microcystis aeruginosa]

Boqueirão gi|488829431|ref|WP_002741837.1| 1 McnC protein [Microcystis aeruginosa]

Boqueirão gi|516352615|ref|WP_017742648.1| 1 non-ribosomal peptide synthetase [Scytonema hofmannii]

Boqueirão gi|516352615|ref|WP_017742648.1| 1 non-ribosomal peptide synthetase [Scytonema hofmannii]

Boqueirão gi|516352615|ref|WP_017742648.1| 1 non-ribosomal peptide synthetase [Scytonema hofmannii]

Boqueirão gi|516352615|ref|WP_017742648.1| 1 non-ribosomal peptide synthetase [Scytonema hofmannii]

Boqueirão gi|488864797|ref|WP_002777036.1| 1 McnC protein [Microcystis aeruginosa]

Boqueirão gi|488864797|ref|WP_002777036.1| 1 McnC protein [Microcystis aeruginosa]

Boqueirão gi|488864797|ref|WP_002777036.1| 1 McnC protein [Microcystis aeruginosa]

Boqueirão gi|488864797|ref|WP_002777036.1| 1 McnC protein [Microcystis aeruginosa]

Boqueirão gi|516352615|ref|WP_017742648.1| 1 non-ribosomal peptide synthetase [Scytonema hofmannii]

Boqueirão gi|910241529|ref|WP_050046520.1| 1 non-ribosomal peptide synthetase [Tolypothrix bouteillei]

Boqueirão gi|504892624|ref|WP_015079726.1| 1 anabaenopeptilide synthetase ApdB [Anabaena sp. 90]

Boqueirão gi|504892624|ref|WP_015079726.1| 1 anabaenopeptilide synthetase ApdB [Anabaena sp. 90]

Boqueirão gi|504892624|ref|WP_015079726.1| 1 anabaenopeptilide synthetase ApdB [Anabaena sp. 90]

Boqueirão gi|503099779|ref|WP_013334576.1| 1 non-ribosomal peptide synthetase [Cyanothece sp. PCC 7822]

Boqueirão gi|501601441|ref|WP_012599817.1| 1 non-ribosomal peptide synthetase [Cyanothece sp. PCC 7424]

Boqueirão gi|653154931|ref|WP_027403948.1| 1 non-ribosomal peptide synthetase [Aphanizomenon flos-aquae]

Boqueirão gi|653154930|ref|WP_027403947.1| 1 non-ribosomal peptide synthetase [Aphanizomenon flos-aquae]

Boqueirão gi|653154930|ref|WP_027403947.1| 1 non-ribosomal peptide synthetase [Aphanizomenon flos-aquae]

Boqueirão gi|1028121172|ref|WP_063874469.1| 1 non-ribosomal peptide synthetase [Nodularia spumigena]

Boqueirão gi|1028121173|ref|WP_063874470.1| 1 non-ribosomal peptide synthetase [Nodularia spumigena]

Boqueirão gi|1028121173|ref|WP_063874470.1| 1 non-ribosomal peptide synthetase [Nodularia spumigena]

Boqueirão gi|516352606|ref|WP_017742639.1| 1 non-ribosomal peptide synthetase [Scytonema hofmannii]

Boqueirão gi|1028121172|ref|WP_063874469.1| 1 non-ribosomal peptide synthetase [Nodularia spumigena]

Boqueirão gi|1060038995|ref|WP_069068162.1| 1 non-ribosomal peptide synthetase [Nostoc sp. KVJ20]

Boqueirão gi|516352606|ref|WP_017742639.1| 1 non-ribosomal peptide synthetase [Scytonema hofmannii]

Boqueirão gi|1121332335|ref|WP_073640234.1| 1 non-ribosomal peptide synthetase [Nostoc calcicola]

Boqueirão gi|1121324733|ref|WP_073634530.1| 1 non-ribosomal peptide synthetase [Scytonema sp. HK-05]

Boqueirão gi|518326645|ref|WP_019496852.1| 1 non-ribosomal peptide synthetase [Calothrix sp. PCC 7103]

Boqueirão gi|518326645|ref|WP_019496852.1| 1 non-ribosomal peptide synthetase [Calothrix sp. PCC 7103]

Boqueirão gi|959925032|ref|WP_058184099.1| 1 non-ribosomal peptide synthetase [Mastigocoleus testarum]

Boqueirão gi|959925032|ref|WP_058184099.1| 1 non-ribosomal peptide synthetase [Mastigocoleus testarum]

Boqueirão gi|959925032|ref|WP_058184099.1| 1 non-ribosomal peptide synthetase [Mastigocoleus testarum]

Boqueirão gi|1094112871|ref|WP_071102370.1| 1 hypothetical protein [Moorea producens]

Boqueirão gi|1084331025|ref|WP_070395084.1| 1 hypothetical protein [Moorea producens]

Boqueirão gi|515877169|ref|WP_017307752.1| 1 hypothetical protein [Fischerella sp. PCC 9339]

Boqueirão gi|1028121172|ref|WP_063874469.1| 1 non-ribosomal peptide synthetase [Nodularia spumigena]

Boqueirão gi|653154931|ref|WP_027403948.1| 1 non-ribosomal peptide synthetase [Aphanizomenon flos-aquae]

Boqueirão gi|504891877|ref|WP_015078979.1| 1 non-ribosomal peptide synthetase [Anabaena sp. 90]

Boqueirão gi|493211331|ref|WP_006196342.1| 1 non-ribosomal peptide synthetase [Nodularia spumigena]

Boqueirão gi|1121324733|ref|WP_073634530.1| 1 non-ribosomal peptide synthetase [Scytonema sp. HK-05]

Boqueirão gi|653154930|ref|WP_027403947.1| 1 non-ribosomal peptide synthetase [Aphanizomenon flos-aquae]

Boqueirão gi|653154930|ref|WP_027403947.1| 1 non-ribosomal peptide synthetase [Aphanizomenon flos-aquae]

Boqueirão gi|493211329|ref|WP_006196341.1| 1 non-ribosomal peptide synthetase [Nodularia spumigena]

Boqueirão gi|493211329|ref|WP_006196341.1| 1 non-ribosomal peptide synthetase [Nodularia spumigena]

Boqueirão gi|504891876|ref|WP_015078978.1| 1 non-ribosomal peptide synthetase [Anabaena sp. 90]

Boqueirão gi|504891876|ref|WP_015078978.1| 1 non-ribosomal peptide synthetase [Anabaena sp. 90]

Boqueirão gi|1121332337|ref|WP_073640236.1| 1 non-ribosomal peptide synthetase [Nostoc calcicola]

Boqueirão gi|1121332337|ref|WP_073640236.1| 1 non-ribosomal peptide synthetase [Nostoc calcicola]

Boqueirão gi|1121324736|ref|WP_073634532.1| 1 non-ribosomal peptide synthetase [Scytonema sp. HK-05]

Boqueirão gi|1121324736|ref|WP_073634532.1| 1 non-ribosomal peptide synthetase [Scytonema sp. HK-05]

Boqueirão gi|1114016452|ref|WP_072040811.1| 1 hypothetical protein [Tolypothrix bouteillei]

Boqueirão gi|1114016452|ref|WP_072040811.1| 1 hypothetical protein [Tolypothrix bouteillei]

Boqueirão gi|496174182|ref|WP_008898689.1| 1 non-ribosomal peptide synthetase [Rheinheimera sp. A13L]

Boqueirão gi|1021719958|ref|WP_063364357.1| 1 non-ribosomal peptide synthetase [Pseudoalteromonas luteoviolacea]

Boqueirão gi|1062970745|ref|WP_069351442.1| 1 non-ribosomal peptide synthetase

Boqueirão gi|910210240|ref|WP_050021259.1| 1 non-ribosomal peptide synthetase [Chryseobacterium sp. P1-3]

Boqueirão gi|916817351|ref|WP_051424407.1| 1 hypothetical protein [Aphanizomenon flos-aquae]

Boqueirão gi|910240616|ref|WP_050045607.1| 1 non-ribosomal peptide synthetase

Boqueirão gi|910240616|ref|WP_050045607.1| 1 non-ribosomal peptide synthetase

Boqueirão gi|910240616|ref|WP_050045607.1| 1 non-ribosomal peptide synthetase

Boqueirão gi|910240616|ref|WP_050045607.1| 1 non-ribosomal peptide synthetase

Boqueirão gi|1062968423|ref|WP_069350655.1| 1 hypothetical protein [Scytonema millei]

Boqueirão gi|501381339|ref|WP_012412905.1| 1 non-ribosomal peptide synthetase [Nostoc punctiforme]

Boqueirão gi|501381339|ref|WP_012412905.1| 1 non-ribosomal peptide synthetase [Nostoc punctiforme]

Boqueirão gi|501381339|ref|WP_012412905.1| 1 non-ribosomal peptide synthetase [Nostoc punctiforme]

Boqueirão gi|516352615|ref|WP_017742648.1| 1 non-ribosomal peptide synthetase [Scytonema hofmannii]

Boqueirão gi|516352615|ref|WP_017742648.1| 1 non-ribosomal peptide synthetase [Scytonema hofmannii]

Boqueirão gi|516352615|ref|WP_017742648.1| 1 non-ribosomal peptide synthetase [Scytonema hofmannii]

Boqueirão gi|516352615|ref|WP_017742648.1| 1 non-ribosomal peptide synthetase [Scytonema hofmannii]

Boqueirão gi|516352615|ref|WP_017742648.1| 1 non-ribosomal peptide synthetase [Scytonema hofmannii]

Boqueirão gi|516352623|ref|WP_017742656.1| 1 non-ribosomal peptide synthetase [Scytonema hofmannii]

Boqueirão gi|516352623|ref|WP_017742656.1| 1 non-ribosomal peptide synthetase [Scytonema hofmannii]

Boqueirão gi|516352623|ref|WP_017742656.1| 1 non-ribosomal peptide synthetase [Scytonema hofmannii]

Boqueirão gi|516352623|ref|WP_017742656.1| 1 non-ribosomal peptide synthetase [Scytonema hofmannii]

Boqueirão gi|504892624|ref|WP_015079726.1| 1 anabaenopeptilide synthetase ApdB [Anabaena sp. 90]

Boqueirão gi|504892624|ref|WP_015079726.1| 1 anabaenopeptilide synthetase ApdB [Anabaena sp. 90]

Boqueirão gi|504892624|ref|WP_015079726.1| 1 anabaenopeptilide synthetase ApdB [Anabaena sp. 90]

Boqueirão gi|1121319237|ref|WP_073629376.1| 1 non-ribosomal peptide synthetase [Scytonema sp. HK-05]

Boqueirão gi|1121319237|ref|WP_073629376.1| 1 non-ribosomal peptide synthetase [Scytonema sp. HK-05]

Boqueirão gi|1121319237|ref|WP_073629376.1| 1 non-ribosomal peptide synthetase [Scytonema sp. HK-05]

Boqueirão gi|516352615|ref|WP_017742648.1| 1 non-ribosomal peptide synthetase [Scytonema hofmannii]

Boqueirão gi|516352615|ref|WP_017742648.1| 1 non-ribosomal peptide synthetase [Scytonema hofmannii]

Boqueirão gi|516352615|ref|WP_017742648.1| 1 non-ribosomal peptide synthetase [Scytonema hofmannii]

Boqueirão gi|516352615|ref|WP_017742648.1| 1 non-ribosomal peptide synthetase [Scytonema hofmannii]

Boqueirão gi|516352615|ref|WP_017742648.1| 1 non-ribosomal peptide synthetase [Scytonema hofmannii]

Boqueirão gi|1056313851|ref|WP_067768741.1| 1 hypothetical protein [Nostoc sp. NIES-3756]

Boqueirão gi|1028121172|ref|WP_063874469.1| 1 non-ribosomal peptide synthetase [Nodularia spumigena]

Boqueirão gi|1121332335|ref|WP_073640234.1| 1 non-ribosomal peptide synthetase [Nostoc calcicola]

Boqueirão gi|493211331|ref|WP_006196342.1| 1 non-ribosomal peptide synthetase [Nodularia spumigena]

Boqueirão gi|501377452|ref|WP_012409018.1| 1 non-ribosomal peptide synthetase [Nostoc punctiforme]

Boqueirão gi|1060038995|ref|WP_069068162.1| 1 non-ribosomal peptide synthetase [Nostoc sp. KVJ20]

Boqueirão gi|916817351|ref|WP_051424407.1| 1 hypothetical protein [Aphanizomenon flos-aquae]

Boqueirão gi|488879468|ref|WP_002791693.1| 1 non-ribosomal peptide synthetase [Microcystis aeruginosa]

Boqueirão gi|501377217|ref|WP_012408783.1| 1 non-ribosomal peptide synthetase [Nostoc punctiforme]

Boqueirão gi|501225155|ref|WP_012268173.1| 1 McnA protein [Microcystis aeruginosa]

Boqueirão gi|1054850717|ref|WP_066612889.1| 1 non-ribosomal peptide synthetase [Scytonema hofmannii]

Boqueirão gi|1011379822|ref|WP_062292587.1| 1 AMP-dependent synthetase [Nostoc piscinale]

Boqueirão gi|916817352|ref|WP_051424408.1| 1 AMP-dependent synthetase [Aphanizomenon flos-aquae]

Boqueirão gi|1102823728|ref|WP_071604959.1| 1 hypothetical protein [Dolichospermum circinale]

Boqueirão gi|924655931|ref|WP_053538952.1| 1 non-ribosomal peptide synthase [Anabaena sp. wa102]

Boqueirão gi|924655931|ref|WP_053538952.1| 1 non-ribosomal peptide synthase [Anabaena sp. wa102]

Boqueirão gi|1121324727|ref|WP_073634527.1| 1 AMP-dependent synthetase [Scytonema sp. HK-05]

Boqueirão gi|493211327|ref|WP_006196339.1| 1 non-ribosomal peptide synthetase [Nodularia spumigena]

Boqueirão gi|1028121175|ref|WP_063874472.1| 1 non-ribosomal peptide synthetase [Nodularia spumigena]

Boqueirão gi|653154928|ref|WP_027403945.1| 1 non-ribosomal peptide synthetase [Aphanizomenon flos-aquae]

Boqueirão gi|504891874|ref|WP_015078976.1| 1 non-ribosomal peptide synthetase [Anabaena sp. 90]

Boqueirão gi|1121324739|ref|WP_073634534.1| 1 non-ribosomal peptide synthetase [Scytonema sp. HK-05]

Boqueirão gi|1130427778|ref|WP_075596525.1| 1 non-ribosomal peptide synthetase [Oscillatoriales cyanobacterium 'hensonii']

Boqueirão gi|515897710|ref|WP_017328293.1| 1 non-ribosomal peptide synthetase [Synechococcus sp. PCC 7336]

Boqueirão gi|505022645|ref|WP_015209747.1| 1 non-ribosomal peptide synthetase [Cylindrospermum stagnale]

Boqueirão gi|504990110|ref|WP_015177212.1| 1 non-ribosomal peptide synthetase [Oscillatoria nigro-viridis]

Boqueirão gi|494594494|ref|WP_007353060.1| 1 MULTISPECIES: non-ribosomal peptide synthetase [Kamptonema]

Boqueirão gi|653154928|ref|WP_027403945.1| 1 non-ribosomal peptide synthetase [Aphanizomenon flos-aquae]

Boqueirão gi|1028121175|ref|WP_063874472.1| 1 non-ribosomal peptide synthetase [Nodularia spumigena]

Boqueirão gi|493211327|ref|WP_006196339.1| 1 non-ribosomal peptide synthetase [Nodularia spumigena]

Boqueirão gi|1121324739|ref|WP_073634534.1| 1 non-ribosomal peptide synthetase [Scytonema sp. HK-05]

Boqueirão gi|1121332341|ref|WP_073640240.1| 1 non-ribosomal peptide synthetase [Nostoc calcicola]

Boqueirão gi|1028121173|ref|WP_063874470.1| 1 non-ribosomal peptide synthetase [Nodularia spumigena]

Boqueirão gi|1121324736|ref|WP_073634532.1| 1 non-ribosomal peptide synthetase [Scytonema sp. HK-05]

Boqueirão gi|653154930|ref|WP_027403947.1| 1 non-ribosomal peptide synthetase [Aphanizomenon flos-aquae]

Boqueirão gi|501377451|ref|WP_012409017.1| 1 non-ribosomal peptide synthetase [Nostoc punctiforme]

Boqueirão gi|493036905|ref|WP_006104279.1| 1 non-ribosomal peptide synthetase [Coleofasciculus chthonoplastes]

Boqueirão gi|493036905|ref|WP_006104279.1| 1 non-ribosomal peptide synthetase [Coleofasciculus chthonoplastes]

Boqueirão gi|516352615|ref|WP_017742648.1| 1 non-ribosomal peptide synthetase [Scytonema hofmannii]

Boqueirão gi|754792464|ref|WP_042156042.1| 1 McnC protein [Planktothrix agardhii]

Boqueirão gi|653003249|ref|WP_027255435.1| 1 McnC protein [Planktothrix agardhii]

Boqueirão gi|652997906|ref|WP_027250316.1| 1 McnC protein [Planktothrix agardhii]

Boqueirão gi|652402732|ref|WP_026798523.1| 1 McnC protein [Planktothrix prolifica]

Boqueirão gi|1028121174|ref|WP_063874471.1| 1 non-ribosomal peptide synthetase [Nodularia spumigena]

Boqueirão gi|653154929|ref|WP_027403946.1| 1 non-ribosomal peptide synthetase [Aphanizomenon flos-aquae]

Boqueirão gi|493211328|ref|WP_006196340.1| 1 non-ribosomal peptide synthetase [Nodularia spumigena]

Boqueirão gi|1060038990|ref|WP_069068159.1| 1 non-ribosomal peptide synthetase [Nostoc sp. KVJ20]

Boqueirão gi|504891875|ref|WP_015078977.1| 1 non-ribosomal peptide synthetase [Anabaena sp. 90]

Boqueirão gi|916817351|ref|WP_051424407.1| 1 hypothetical protein [Aphanizomenon flos-aquae]

Boqueirão gi|501377217|ref|WP_012408783.1| 1 non-ribosomal peptide synthetase [Nostoc punctiforme]

Boqueirão gi|1054850717|ref|WP_066612889.1| 1 non-ribosomal peptide synthetase [Scytonema hofmannii]

Boqueirão gi|488864799|ref|WP_002777038.1| 1 non-ribosomal peptide synthetase [Microcystis aeruginosa]

Boqueirão gi|488879468|ref|WP_002791693.1| 1 non-ribosomal peptide synthetase [Microcystis aeruginosa]

Boqueirão gi|1028121174|ref|WP_063874471.1| 1 non-ribosomal peptide synthetase [Nodularia spumigena]

Boqueirão gi|501377450|ref|WP_012409016.1| 1 non-ribosomal peptide synthetase [Nostoc punctiforme]

Boqueirão gi|1121332339|ref|WP_073640238.1| 1 non-ribosomal peptide synthetase [Nostoc calcicola]

Boqueirão gi|653154929|ref|WP_027403946.1| 1 non-ribosomal peptide synthetase [Aphanizomenon flos-aquae]

Boqueirão gi|493211328|ref|WP_006196340.1| 1 non-ribosomal peptide synthetase [Nodularia spumigena]

Boqueirão gi|1056313851|ref|WP_067768741.1| 1 hypothetical protein [Nostoc sp. NIES-3756]

Boqueirão gi|1056313851|ref|WP_067768741.1| 1 hypothetical protein [Nostoc sp. NIES-3756]

Boqueirão gi|504892625|ref|WP_015079727.1| 1 anabaenopeptilide synthetase ApdA [Anabaena sp. 90]

Boqueirão gi|501377219|ref|WP_012408785.1| 1 non-ribosomal peptide synthetase [Nostoc punctiforme]

Boqueirão gi|501377219|ref|WP_012408785.1| 1 non-ribosomal peptide synthetase [Nostoc punctiforme]

Boqueirão gi|516352623|ref|WP_017742656.1| 1 non-ribosomal peptide synthetase [Scytonema hofmannii]

Boqueirão gi|516352623|ref|WP_017742656.1| 1 non-ribosomal peptide synthetase [Scytonema hofmannii]

Boqueirão gi|516352623|ref|WP_017742656.1| 1 non-ribosomal peptide synthetase [Scytonema hofmannii]

Boqueirão gi|516352623|ref|WP_017742656.1| 1 non-ribosomal peptide synthetase [Scytonema hofmannii]

Boqueirão gi|653154922|ref|WP_027403939.1| 1 non-ribosomal peptide synthetase [Aphanizomenon flos-aquae]

Boqueirão gi|653154922|ref|WP_027403939.1| 1 non-ribosomal peptide synthetase [Aphanizomenon flos-aquae]

Boqueirão gi|493564253|ref|WP_006517624.1| 1 non-ribosomal peptide synthase [Leptolyngbya sp. PCC 7375]

Boqueirão gi|1028121172|ref|WP_063874469.1| 1 non-ribosomal peptide synthetase [Nodularia spumigena]

Boqueirão gi|493211331|ref|WP_006196342.1| 1 non-ribosomal peptide synthetase [Nodularia spumigena]

Boqueirão gi|516352606|ref|WP_017742639.1| 1 non-ribosomal peptide synthetase [Scytonema hofmannii]

Boqueirão gi|653154931|ref|WP_027403948.1| 1 non-ribosomal peptide synthetase [Aphanizomenon flos-aquae]

Boqueirão gi|501377452|ref|WP_012409018.1| 1 non-ribosomal peptide synthetase [Nostoc punctiforme]

Boqueirão gi|518316706|ref|WP_019486913.1| 1 non-ribosomal peptide synthetase [Kamptonema formosum]

Boqueirão gi|959924793|ref|WP_058183914.1| 1 non-ribosomal peptide synthetase [Mastigocoleus testarum]

Boqueirão gi|959924793|ref|WP_058183914.1| 1 non-ribosomal peptide synthetase [Mastigocoleus testarum]

Boqueirão gi|959924793|ref|WP_058183914.1| 1 non-ribosomal peptide synthetase [Mastigocoleus testarum]

Boqueirão gi|752554753|ref|WP_041225985.1| 1 non-ribosomal peptide synthetase [Crinalium epipsammum]

Boqueirão gi|752554753|ref|WP_041225985.1| 1 non-ribosomal peptide synthetase [Crinalium epipsammum]

Boqueirão gi|752554753|ref|WP_041225985.1| 1 non-ribosomal peptide synthetase [Crinalium epipsammum]

Boqueirão gi|752554753|ref|WP_041225985.1| 1 non-ribosomal peptide synthetase [Crinalium epipsammum]

Boqueirão gi|752554753|ref|WP_041225985.1| 1 non-ribosomal peptide synthetase [Crinalium epipsammum]

Boqueirão gi|1132228316|ref|WP_075896497.1| 1 hypothetical protein [Moorea bouillonii]

Boqueirão gi|1132228316|ref|WP_075896497.1| 1 hypothetical protein [Moorea bouillonii]

Boqueirão gi|916817351|ref|WP_051424407.1| 1 hypothetical protein [Aphanizomenon flos-aquae]

Boqueirão gi|488879468|ref|WP_002791693.1| 1 non-ribosomal peptide synthetase [Microcystis aeruginosa]

Boqueirão gi|490389201|ref|WP_004268656.1| 1 non-ribosomal peptide synthetase [Microcystis aeruginosa]

Boqueirão gi|488864799|ref|WP_002777038.1| 1 non-ribosomal peptide synthetase [Microcystis aeruginosa]

Boqueirão gi|763119425|ref|WP_043999339.1| 1 non-ribosomal peptide synthetase

Boqueirão gi|653154922|ref|WP_027403939.1| 1 non-ribosomal peptide synthetase [Aphanizomenon flos-aquae]

Boqueirão gi|653154922|ref|WP_027403939.1| 1 non-ribosomal peptide synthetase [Aphanizomenon flos-aquae]

Boqueirão gi|1062968423|ref|WP_069350655.1| 1 hypothetical protein [Scytonema millei]

Boqueirão gi|1062968423|ref|WP_069350655.1| 1 hypothetical protein [Scytonema millei]

Boqueirão gi|516352615|ref|WP_017742648.1| 1 non-ribosomal peptide synthetase [Scytonema hofmannii]

Boqueirão gi|516352615|ref|WP_017742648.1| 1 non-ribosomal peptide synthetase [Scytonema hofmannii]

Boqueirão gi|516352615|ref|WP_017742648.1| 1 non-ribosomal peptide synthetase [Scytonema hofmannii]

Boqueirão gi|516352615|ref|WP_017742648.1| 1 non-ribosomal peptide synthetase [Scytonema hofmannii]

Boqueirão gi|516352615|ref|WP_017742648.1| 1 non-ribosomal peptide synthetase [Scytonema hofmannii]

Boqueirão gi|910240616|ref|WP_050045607.1| 1 non-ribosomal peptide synthetase

Boqueirão gi|910240616|ref|WP_050045607.1| 1 non-ribosomal peptide synthetase

Boqueirão gi|910240616|ref|WP_050045607.1| 1 non-ribosomal peptide synthetase

Boqueirão gi|910240616|ref|WP_050045607.1| 1 non-ribosomal peptide synthetase

Boqueirão gi|516352616|ref|WP_017742649.1| 1 non-ribosomal peptide synthetase [Scytonema hofmannii]

Boqueirão gi|653154931|ref|WP_027403948.1| 1 non-ribosomal peptide synthetase [Aphanizomenon flos-aquae]

Boqueirão gi|493211329|ref|WP_006196341.1| 1 non-ribosomal peptide synthetase [Nodularia spumigena]

Boqueirão gi|493211329|ref|WP_006196341.1| 1 non-ribosomal peptide synthetase [Nodularia spumigena]

Boqueirão gi|1028121172|ref|WP_063874469.1| 1 non-ribosomal peptide synthetase [Nodularia spumigena]

Boqueirão gi|504891876|ref|WP_015078978.1| 1 non-ribosomal peptide synthetase [Anabaena sp. 90]

Boqueirão gi|504891876|ref|WP_015078978.1| 1 non-ribosomal peptide synthetase [Anabaena sp. 90]

Boqueirão gi|493211331|ref|WP_006196342.1| 1 non-ribosomal peptide synthetase [Nodularia spumigena]

Boqueirão gi|653154928|ref|WP_027403945.1| 1 non-ribosomal peptide synthetase [Aphanizomenon flos-aquae]

Boqueirão gi|493211327|ref|WP_006196339.1| 1 non-ribosomal peptide synthetase [Nodularia spumigena]

Boqueirão gi|1028121175|ref|WP_063874472.1| 1 non-ribosomal peptide synthetase [Nodularia spumigena]

Boqueirão gi|1121324739|ref|WP_073634534.1| 1 non-ribosomal peptide synthetase [Scytonema sp. HK-05]

Boqueirão gi|501377948|ref|WP_012409514.1| 1 non-ribosomal peptide synthetase [Nostoc punctiforme]

Boqueirão gi|1028121172|ref|WP_063874469.1| 1 non-ribosomal peptide synthetase [Nodularia spumigena]

Boqueirão gi|501377452|ref|WP_012409018.1| 1 non-ribosomal peptide synthetase [Nostoc punctiforme]

Boqueirão gi|1121332335|ref|WP_073640234.1| 1 non-ribosomal peptide synthetase [Nostoc calcicola]

Boqueirão gi|493211331|ref|WP_006196342.1| 1 non-ribosomal peptide synthetase [Nodularia spumigena]

Boqueirão gi|504891877|ref|WP_015078979.1| 1 non-ribosomal peptide synthetase [Anabaena sp. 90]

Boqueirão gi|653154928|ref|WP_027403945.1| 1 non-ribosomal peptide synthetase [Aphanizomenon flos-aquae]

Boqueirão gi|653154928|ref|WP_027403945.1| 1 non-ribosomal peptide synthetase [Aphanizomenon flos-aquae]

Boqueirão gi|1028121175|ref|WP_063874472.1| 1 non-ribosomal peptide synthetase [Nodularia spumigena]

Boqueirão gi|1028121175|ref|WP_063874472.1| 1 non-ribosomal peptide synthetase [Nodularia spumigena]

Boqueirão gi|493211327|ref|WP_006196339.1| 1 non-ribosomal peptide synthetase [Nodularia spumigena]

Boqueirão gi|493211327|ref|WP_006196339.1| 1 non-ribosomal peptide synthetase [Nodularia spumigena]

Boqueirão gi|504891874|ref|WP_015078976.1| 1 non-ribosomal peptide synthetase [Anabaena sp. 90]

Boqueirão gi|504891874|ref|WP_015078976.1| 1 non-ribosomal peptide synthetase [Anabaena sp. 90]

Boqueirão gi|1121332340|ref|WP_073640239.1| 1 non-ribosomal peptide synthetase [Nostoc calcicola]

Boqueirão gi|1121332340|ref|WP_073640239.1| 1 non-ribosomal peptide synthetase [Nostoc calcicola]

Boqueirão gi|516352615|ref|WP_017742648.1| 1 non-ribosomal peptide synthetase [Scytonema hofmannii]

Boqueirão gi|516352615|ref|WP_017742648.1| 1 non-ribosomal peptide synthetase [Scytonema hofmannii]

Boqueirão gi|516352615|ref|WP_017742648.1| 1 non-ribosomal peptide synthetase [Scytonema hofmannii]

Boqueirão gi|516352615|ref|WP_017742648.1| 1 non-ribosomal peptide synthetase [Scytonema hofmannii]

Boqueirão gi|516352615|ref|WP_017742648.1| 1 non-ribosomal peptide synthetase [Scytonema hofmannii]

Boqueirão gi|516352623|ref|WP_017742656.1| 1 non-ribosomal peptide synthetase [Scytonema hofmannii]

Boqueirão gi|516352623|ref|WP_017742656.1| 1 non-ribosomal peptide synthetase [Scytonema hofmannii]

Boqueirão gi|516352623|ref|WP_017742656.1| 1 non-ribosomal peptide synthetase [Scytonema hofmannii]

Boqueirão gi|516352623|ref|WP_017742656.1| 1 non-ribosomal peptide synthetase [Scytonema hofmannii]

Boqueirão gi|1054850722|ref|WP_066612894.1| 1 hypothetical protein [Scytonema hofmannii]

Boqueirão gi|1054850722|ref|WP_066612894.1| 1 hypothetical protein [Scytonema hofmannii]

Boqueirão gi|1054850722|ref|WP_066612894.1| 1 hypothetical protein [Scytonema hofmannii]

Boqueirão gi|488839868|ref|WP_002752274.1| 1 MicC protein [Microcystis aeruginosa]

Boqueirão gi|652997906|ref|WP_027250316.1| 1 McnC protein [Planktothrix agardhii]

Boqueirão gi|652997906|ref|WP_027250316.1| 1 McnC protein [Planktothrix agardhii]

Boqueirão gi|652997906|ref|WP_027250316.1| 1 McnC protein [Planktothrix agardhii]

Boqueirão gi|652997906|ref|WP_027250316.1| 1 McnC protein [Planktothrix agardhii]

Boqueirão gi|501377219|ref|WP_012408785.1| 1 non-ribosomal peptide synthetase [Nostoc punctiforme]

Boqueirão gi|1121319243|ref|WP_073629382.1| 1 hypothetical protein [Scytonema sp. HK-05]

Boqueirão gi|1114016452|ref|WP_072040811.1| 1 hypothetical protein [Tolypothrix bouteillei]

Boqueirão gi|1054850722|ref|WP_066612894.1| 1 hypothetical protein [Scytonema hofmannii]

Boqueirão gi|1054850725|ref|WP_066612897.1| 1 hypothetical protein

Boqueirão gi|493211327|ref|WP_006196339.1| 1 non-ribosomal peptide synthetase [Nodularia spumigena]

Boqueirão gi|493211327|ref|WP_006196339.1| 1 non-ribosomal peptide synthetase [Nodularia spumigena]

Boqueirão gi|493211327|ref|WP_006196339.1| 1 non-ribosomal peptide synthetase [Nodularia spumigena]

Boqueirão gi|493211327|ref|WP_006196339.1| 1 non-ribosomal peptide synthetase [Nodularia spumigena]

Boqueirão gi|1028121175|ref|WP_063874472.1| 1 non-ribosomal peptide synthetase [Nodularia spumigena]

Boqueirão gi|1028121175|ref|WP_063874472.1| 1 non-ribosomal peptide synthetase [Nodularia spumigena]

Boqueirão gi|1028121175|ref|WP_063874472.1| 1 non-ribosomal peptide synthetase [Nodularia spumigena]

Boqueirão gi|1028121175|ref|WP_063874472.1| 1 non-ribosomal peptide synthetase [Nodularia spumigena]

Boqueirão gi|653154928|ref|WP_027403945.1| 1 non-ribosomal peptide synthetase [Aphanizomenon flos-aquae]

Boqueirão gi|653154928|ref|WP_027403945.1| 1 non-ribosomal peptide synthetase [Aphanizomenon flos-aquae]

Boqueirão gi|653154928|ref|WP_027403945.1| 1 non-ribosomal peptide synthetase [Aphanizomenon flos-aquae]

Boqueirão gi|653154928|ref|WP_027403945.1| 1 non-ribosomal peptide synthetase [Aphanizomenon flos-aquae]

Boqueirão gi|516352640|ref|WP_017742673.1| 1 non-ribosomal peptide synthetase [Scytonema hofmannii]

Boqueirão gi|516352640|ref|WP_017742673.1| 1 non-ribosomal peptide synthetase [Scytonema hofmannii]

Boqueirão gi|516352640|ref|WP_017742673.1| 1 non-ribosomal peptide synthetase [Scytonema hofmannii]

Boqueirão gi|516352640|ref|WP_017742673.1| 1 non-ribosomal peptide synthetase [Scytonema hofmannii]

Boqueirão gi|516352640|ref|WP_017742673.1| 1 non-ribosomal peptide synthetase [Scytonema hofmannii]

Boqueirão gi|516352640|ref|WP_017742673.1| 1 non-ribosomal peptide synthetase [Scytonema hofmannii]

Boqueirão gi|516352640|ref|WP_017742673.1| 1 non-ribosomal peptide synthetase [Scytonema hofmannii]

Boqueirão gi|516352640|ref|WP_017742673.1| 1 non-ribosomal peptide synthetase [Scytonema hofmannii]

Boqueirão gi|959925032|ref|WP_058184099.1| 1 non-ribosomal peptide synthetase [Mastigocoleus testarum]

Boqueirão gi|959925032|ref|WP_058184099.1| 1 non-ribosomal peptide synthetase [Mastigocoleus testarum]

Boqueirão gi|959925032|ref|WP_058184099.1| 1 non-ribosomal peptide synthetase [Mastigocoleus testarum]

Boqueirão gi|959925032|ref|WP_058184099.1| 1 non-ribosomal peptide synthetase [Mastigocoleus testarum]

Boqueirão gi|959925032|ref|WP_058184099.1| 1 non-ribosomal peptide synthetase [Mastigocoleus testarum]

Boqueirão gi|959925032|ref|WP_058184099.1| 1 non-ribosomal peptide synthetase [Mastigocoleus testarum]

Boqueirão gi|504892624|ref|WP_015079726.1| 1 anabaenopeptilide synthetase ApdB [Anabaena sp. 90]

Boqueirão gi|504892624|ref|WP_015079726.1| 1 anabaenopeptilide synthetase ApdB [Anabaena sp. 90]

Boqueirão gi|504892624|ref|WP_015079726.1| 1 anabaenopeptilide synthetase ApdB [Anabaena sp. 90]

Boqueirão gi|504892624|ref|WP_015079726.1| 1 anabaenopeptilide synthetase ApdB [Anabaena sp. 90]

Boqueirão gi|516352623|ref|WP_017742656.1| 1 non-ribosomal peptide synthetase [Scytonema hofmannii]

Boqueirão gi|516352623|ref|WP_017742656.1| 1 non-ribosomal peptide synthetase [Scytonema hofmannii]

Boqueirão gi|516352623|ref|WP_017742656.1| 1 non-ribosomal peptide synthetase [Scytonema hofmannii]

Boqueirão gi|516352623|ref|WP_017742656.1| 1 non-ribosomal peptide synthetase [Scytonema hofmannii]

Boqueirão gi|652996400|ref|WP_027248867.1| 1 McnC protein [Planktothrix agardhii]

Boqueirão gi|652996400|ref|WP_027248867.1| 1 McnC protein [Planktothrix agardhii]

Boqueirão gi|652996400|ref|WP_027248867.1| 1 McnC protein [Planktothrix agardhii]

Boqueirão gi|652996400|ref|WP_027248867.1| 1 McnC protein [Planktothrix agardhii]

Boqueirão gi|652997906|ref|WP_027250316.1| 1 McnC protein [Planktothrix agardhii]

Boqueirão gi|652997906|ref|WP_027250316.1| 1 McnC protein [Planktothrix agardhii]

Boqueirão gi|652997906|ref|WP_027250316.1| 1 McnC protein [Planktothrix agardhii]

Boqueirão gi|652997906|ref|WP_027250316.1| 1 McnC protein [Planktothrix agardhii]

Boqueirão gi|652402429|ref|WP_026798225.1| 1 McnC protein [Planktothrix prolifica]

Boqueirão gi|652402429|ref|WP_026798225.1| 1 McnC protein [Planktothrix prolifica]

Boqueirão gi|652402429|ref|WP_026798225.1| 1 McnC protein [Planktothrix prolifica]

Boqueirão gi|652402429|ref|WP_026798225.1| 1 McnC protein [Planktothrix prolifica]

Boqueirão gi|488864799|ref|WP_002777038.1| 1 non-ribosomal peptide synthetase [Microcystis aeruginosa]

Boqueirão gi|490389201|ref|WP_004268656.1| 1 non-ribosomal peptide synthetase [Microcystis aeruginosa]

Boqueirão gi|916817351|ref|WP_051424407.1| 1 hypothetical protein [Aphanizomenon flos-aquae]

Boqueirão gi|488838362|ref|WP_002750768.1| 1 non-ribosomal peptide synthetase [Microcystis aeruginosa]

Boqueirão gi|652996399|ref|WP_027248866.1| 1 non-ribosomal peptide synthetase [Planktothrix agardhii]

Boqueirão gi|505006764|ref|WP_015193866.1| 1 non-ribosomal peptide synthetase [Stanieria cyanosphaera]

Boqueirão gi|1011354298|ref|WP_062268172.1| 1 non-ribosomal peptide synthetase [Caballeronia megalochromosomata]

Boqueirão gi|1011354298|ref|WP_062268172.1| 1 non-ribosomal peptide synthetase [Caballeronia megalochromosomata]

Boqueirão gi|1121319251|ref|WP_073629390.1| 1 non-ribosomal peptide synthetase [Scytonema sp. HK-05]

Boqueirão gi|1121324035|ref|WP_073634136.1| 1 non-ribosomal peptide synthetase [Scytonema sp. HK-05]

Boqueirão gi|518316706|ref|WP_019486913.1| 1 non-ribosomal peptide synthetase [Kamptonema formosum]

Boqueirão gi|1054850722|ref|WP_066612894.1| 1 hypothetical protein [Scytonema hofmannii]

Boqueirão gi|1054850722|ref|WP_066612894.1| 1 hypothetical protein [Scytonema hofmannii]

Boqueirão gi|516352623|ref|WP_017742656.1| 1 non-ribosomal peptide synthetase [Scytonema hofmannii]

Boqueirão gi|516352623|ref|WP_017742656.1| 1 non-ribosomal peptide synthetase [Scytonema hofmannii]

Boqueirão gi|516352623|ref|WP_017742656.1| 1 non-ribosomal peptide synthetase [Scytonema hofmannii]

Boqueirão gi|516352623|ref|WP_017742656.1| 1 non-ribosomal peptide synthetase [Scytonema hofmannii]

Boqueirão gi|652996399|ref|WP_027248866.1| 1 non-ribosomal peptide synthetase [Planktothrix agardhii]

Boqueirão gi|652996399|ref|WP_027248866.1| 1 non-ribosomal peptide synthetase [Planktothrix agardhii]

Boqueirão gi|652996399|ref|WP_027248866.1| 1 non-ribosomal peptide synthetase [Planktothrix agardhii]

Boqueirão gi|504892624|ref|WP_015079726.1| 1 anabaenopeptilide synthetase ApdB [Anabaena sp. 90]

Boqueirão gi|504892624|ref|WP_015079726.1| 1 anabaenopeptilide synthetase ApdB [Anabaena sp. 90]

Boqueirão gi|504892624|ref|WP_015079726.1| 1 anabaenopeptilide synthetase ApdB [Anabaena sp. 90]

Boqueirão gi|504892624|ref|WP_015079726.1| 1 anabaenopeptilide synthetase ApdB [Anabaena sp. 90]

Boqueirão gi|1011379797|ref|WP_062292562.1| 1 non-ribosomal peptide synthetase [Nostoc piscinale]

Boqueirão gi|1011379797|ref|WP_062292562.1| 1 non-ribosomal peptide synthetase [Nostoc piscinale]

Boqueirão gi|1011379797|ref|WP_062292562.1| 1 non-ribosomal peptide synthetase [Nostoc piscinale]

Boqueirão gi|504891877|ref|WP_015078979.1| 1 non-ribosomal peptide synthetase [Anabaena sp. 90]

Boqueirão gi|1028121172|ref|WP_063874469.1| 1 non-ribosomal peptide synthetase [Nodularia spumigena]

Boqueirão gi|653154931|ref|WP_027403948.1| 1 non-ribosomal peptide synthetase [Aphanizomenon flos-aquae]

Boqueirão gi|493211331|ref|WP_006196342.1| 1 non-ribosomal peptide synthetase [Nodularia spumigena]

Boqueirão gi|501377452|ref|WP_012409018.1| 1 non-ribosomal peptide synthetase [Nostoc punctiforme]

Boqueirão gi|501223609|ref|WP_012266627.1| 1 McyB protein [Microcystis aeruginosa]

Boqueirão gi|1120049985|ref|WP_072923808.1| 1 non-ribosomal peptide synthetase [Microcystis aeruginosa]

Boqueirão gi|1105172442|ref|WP_071825102.1| 1 non-ribosomal peptide synthetase

Boqueirão gi|1002987252|ref|WP_061431779.1| 1 non-ribosomal peptide synthetase [Microcystis aeruginosa]

Boqueirão gi|499306023|ref|WP_010996798.1| 1 non-ribosomal peptide synthetase [Nostoc sp. PCC 7120]

Boqueirão gi|493207403|ref|WP_006194156.1| 1 non-ribosomal peptide synthetase [Nodularia spumigena]

Boqueirão gi|1028119247|ref|WP_063872544.1| 1 non-ribosomal peptide synthetase [Nodularia spumigena]

Boqueirão gi|916303484|ref|WP_051038530.1| 1 non-ribosomal peptide synthetase [Gloeocapsa sp. PCC 7428]

Boqueirão gi|504944771|ref|WP_015131873.1| 1 non-ribosomal peptide synthetase [Calothrix sp. PCC 7507]

Boqueirão gi|501377219|ref|WP_012408785.1| 1 non-ribosomal peptide synthetase [Nostoc punctiforme]

Boqueirão gi|1028121179|ref|WP_063874476.1| 1 non-ribosomal peptide synthetase [Nodularia spumigena]

Boqueirão gi|493215585|ref|WP_006198652.1| 1 non-ribosomal peptide synthetase [Nodularia spumigena]

Boqueirão gi|1121319243|ref|WP_073629382.1| 1 hypothetical protein [Scytonema sp. HK-05]

Boqueirão gi|1114016452|ref|WP_072040811.1| 1 hypothetical protein [Tolypothrix bouteillei]

Boqueirão gi|1028121175|ref|WP_063874472.1| 1 non-ribosomal peptide synthetase [Nodularia spumigena]

Boqueirão gi|493211327|ref|WP_006196339.1| 1 non-ribosomal peptide synthetase [Nodularia spumigena]

Boqueirão gi|653154928|ref|WP_027403945.1| 1 non-ribosomal peptide synthetase [Aphanizomenon flos-aquae]

Boqueirão gi|504891874|ref|WP_015078976.1| 1 non-ribosomal peptide synthetase [Anabaena sp. 90]

Boqueirão gi|652389975|ref|WP_026785823.1| 1 non-ribosomal peptide synthetase [Planktothrix rubescens]

Boqueirão gi|653154928|ref|WP_027403945.1| 1 non-ribosomal peptide synthetase [Aphanizomenon flos-aquae]

Boqueirão gi|653154928|ref|WP_027403945.1| 1 non-ribosomal peptide synthetase [Aphanizomenon flos-aquae]

Boqueirão gi|493211327|ref|WP_006196339.1| 1 non-ribosomal peptide synthetase [Nodularia spumigena]

Boqueirão gi|493211327|ref|WP_006196339.1| 1 non-ribosomal peptide synthetase [Nodularia spumigena]

Boqueirão gi|1028121175|ref|WP_063874472.1| 1 non-ribosomal peptide synthetase [Nodularia spumigena]

Boqueirão gi|1028121175|ref|WP_063874472.1| 1 non-ribosomal peptide synthetase [Nodularia spumigena]

Boqueirão gi|1121332340|ref|WP_073640239.1| 1 non-ribosomal peptide synthetase [Nostoc calcicola]

Boqueirão gi|1121332340|ref|WP_073640239.1| 1 non-ribosomal peptide synthetase [Nostoc calcicola]

Boqueirão gi|504891874|ref|WP_015078976.1| 1 non-ribosomal peptide synthetase [Anabaena sp. 90]

Boqueirão gi|504891874|ref|WP_015078976.1| 1 non-ribosomal peptide synthetase [Anabaena sp. 90]

Boqueirão gi|501377219|ref|WP_012408785.1| 1 non-ribosomal peptide synthetase [Nostoc punctiforme]

Boqueirão gi|493215585|ref|WP_006198652.1| 1 non-ribosomal peptide synthetase [Nodularia spumigena]

Boqueirão gi|1028121179|ref|WP_063874476.1| 1 non-ribosomal peptide synthetase [Nodularia spumigena]

Boqueirão gi|1114016452|ref|WP_072040811.1| 1 hypothetical protein [Tolypothrix bouteillei]

Boqueirão gi|1121319243|ref|WP_073629382.1| 1 hypothetical protein [Scytonema sp. HK-05]

Boqueirão gi|1028121175|ref|WP_063874472.1| 1 non-ribosomal peptide synthetase [Nodularia spumigena]

Boqueirão gi|1028121175|ref|WP_063874472.1| 1 non-ribosomal peptide synthetase [Nodularia spumigena]

Boqueirão gi|493211327|ref|WP_006196339.1| 1 non-ribosomal peptide synthetase [Nodularia spumigena]

Boqueirão gi|493211327|ref|WP_006196339.1| 1 non-ribosomal peptide synthetase [Nodularia spumigena]

Boqueirão gi|653154928|ref|WP_027403945.1| 1 non-ribosomal peptide synthetase [Aphanizomenon flos-aquae]

Boqueirão gi|653154928|ref|WP_027403945.1| 1 non-ribosomal peptide synthetase [Aphanizomenon flos-aquae]

Boqueirão gi|504891874|ref|WP_015078976.1| 1 non-ribosomal peptide synthetase [Anabaena sp. 90]

Boqueirão gi|504891874|ref|WP_015078976.1| 1 non-ribosomal peptide synthetase [Anabaena sp. 90]

Boqueirão gi|919116792|ref|WP_052672433.1| 1 non-ribosomal peptide synthetase [Aliterella atlantica]

Boqueirão gi|919116792|ref|WP_052672433.1| 1 non-ribosomal peptide synthetase [Aliterella atlantica]

Boqueirão gi|919116792|ref|WP_052672433.1| 1 non-ribosomal peptide synthetase [Aliterella atlantica]

Boqueirão gi|493211327|ref|WP_006196339.1| 1 non-ribosomal peptide synthetase [Nodularia spumigena]

Boqueirão gi|493211327|ref|WP_006196339.1| 1 non-ribosomal peptide synthetase [Nodularia spumigena]

Boqueirão gi|1028121175|ref|WP_063874472.1| 1 non-ribosomal peptide synthetase [Nodularia spumigena]

Boqueirão gi|1028121175|ref|WP_063874472.1| 1 non-ribosomal peptide synthetase [Nodularia spumigena]

Boqueirão gi|653154928|ref|WP_027403945.1| 1 non-ribosomal peptide synthetase [Aphanizomenon flos-aquae]

Boqueirão gi|653154928|ref|WP_027403945.1| 1 non-ribosomal peptide synthetase [Aphanizomenon flos-aquae]

Boqueirão gi|504991557|ref|WP_015178659.1| 1 non-ribosomal peptide synthetase [Oscillatoria nigro-viridis]

Boqueirão gi|1096253496|ref|WP_071191492.1| 1 hypothetical protein [Trichormus sp. NMC-1]

Boqueirão gi|653154929|ref|WP_027403946.1| 1 non-ribosomal peptide synthetase [Aphanizomenon flos-aquae]

Boqueirão gi|1028121174|ref|WP_063874471.1| 1 non-ribosomal peptide synthetase [Nodularia spumigena]

Boqueirão gi|493211328|ref|WP_006196340.1| 1 non-ribosomal peptide synthetase [Nodularia spumigena]

Boqueirão gi|501377450|ref|WP_012409016.1| 1 non-ribosomal peptide synthetase [Nostoc punctiforme]

Boqueirão gi|516352608|ref|WP_017742641.1| 1 non-ribosomal peptide synthetase [Scytonema hofmannii]

Boqueirão gi|653154930|ref|WP_027403947.1| 1 non-ribosomal peptide synthetase [Aphanizomenon flos-aquae]

Boqueirão gi|653154930|ref|WP_027403947.1| 1 non-ribosomal peptide synthetase [Aphanizomenon flos-aquae]

Boqueirão gi|1028121173|ref|WP_063874470.1| 1 non-ribosomal peptide synthetase [Nodularia spumigena]

Boqueirão gi|1028121173|ref|WP_063874470.1| 1 non-ribosomal peptide synthetase [Nodularia spumigena]

Boqueirão gi|504891876|ref|WP_015078978.1| 1 non-ribosomal peptide synthetase [Anabaena sp. 90]

Boqueirão gi|504891876|ref|WP_015078978.1| 1 non-ribosomal peptide synthetase [Anabaena sp. 90]

Boqueirão gi|501377451|ref|WP_012409017.1| 1 non-ribosomal peptide synthetase [Nostoc punctiforme]

Boqueirão gi|501377451|ref|WP_012409017.1| 1 non-ribosomal peptide synthetase [Nostoc punctiforme]

Boqueirão gi|493211329|ref|WP_006196341.1| 1 non-ribosomal peptide synthetase [Nodularia spumigena]

Boqueirão gi|493211329|ref|WP_006196341.1| 1 non-ribosomal peptide synthetase [Nodularia spumigena]

Boqueirão gi|504891876|ref|WP_015078978.1| 1 non-ribosomal peptide synthetase [Anabaena sp. 90]

Boqueirão gi|493211329|ref|WP_006196341.1| 1 non-ribosomal peptide synthetase [Nodularia spumigena]

Boqueirão gi|493211329|ref|WP_006196341.1| 1 non-ribosomal peptide synthetase [Nodularia spumigena]

Boqueirão gi|1028121173|ref|WP_063874470.1| 1 non-ribosomal peptide synthetase [Nodularia spumigena]

Boqueirão gi|653154930|ref|WP_027403947.1| 1 non-ribosomal peptide synthetase [Aphanizomenon flos-aquae]

Boqueirão gi|501377452|ref|WP_012409018.1| 1 non-ribosomal peptide synthetase [Nostoc punctiforme]

Boqueirão gi|1028121172|ref|WP_063874469.1| 1 non-ribosomal peptide synthetase [Nodularia spumigena]

Boqueirão gi|1121324733|ref|WP_073634530.1| 1 non-ribosomal peptide synthetase [Scytonema sp. HK-05]

Boqueirão gi|1121332335|ref|WP_073640234.1| 1 non-ribosomal peptide synthetase [Nostoc calcicola]

Boqueirão gi|493211331|ref|WP_006196342.1| 1 non-ribosomal peptide synthetase [Nodularia spumigena]

Boqueirão gi|516352606|ref|WP_017742639.1| 1 non-ribosomal peptide synthetase [Scytonema hofmannii]

Boqueirão gi|653154922|ref|WP_027403939.1| 1 non-ribosomal peptide synthetase [Aphanizomenon flos-aquae]

Boqueirão gi|653154922|ref|WP_027403939.1| 1 non-ribosomal peptide synthetase [Aphanizomenon flos-aquae]

Boqueirão gi|1121319237|ref|WP_073629376.1| 1 non-ribosomal peptide synthetase [Scytonema sp. HK-05]

Boqueirão gi|1121319237|ref|WP_073629376.1| 1 non-ribosomal peptide synthetase [Scytonema sp. HK-05]

Boqueirão gi|652402430|ref|WP_026798226.1| 1 non-ribosomal peptide synthetase

Boqueirão gi|918105860|ref|WP_052331149.1| 1 non-ribosomal peptide synthetase [Planktothrix agardhii]

Boqueirão gi|918105860|ref|WP_052331149.1| 1 non-ribosomal peptide synthetase [Planktothrix agardhii]

Boqueirão gi|516352615|ref|WP_017742648.1| 1 non-ribosomal peptide synthetase [Scytonema hofmannii]

Boqueirão gi|516352615|ref|WP_017742648.1| 1 non-ribosomal peptide synthetase [Scytonema hofmannii]

Boqueirão gi|516352615|ref|WP_017742648.1| 1 non-ribosomal peptide synthetase [Scytonema hofmannii]

Boqueirão gi|516352615|ref|WP_017742648.1| 1 non-ribosomal peptide synthetase [Scytonema hofmannii]

Saulo Maia gi|1054659674|ref|WP_066426813.1| 1 non-ribosomal peptide synthetase [Anabaena sp. 4-3]

Saulo Maia gi|1054615292|ref|WP_066383174.1| 1 non-ribosomal peptide synthetase [Anabaena sp. CA = ATCC 33047]

Saulo Maia gi|501381216|ref|WP_012412782.1| 1 non-ribosomal peptide synthetase [Nostoc punctiforme]

Saulo Maia gi|516355639|ref|WP_017745672.1| 1 non-ribosomal peptide synthetase [Scytonema hofmannii]

Saulo Maia gi|516355639|ref|WP_017745672.1| 1 non-ribosomal peptide synthetase [Scytonema hofmannii]

Saulo Maia gi|488880990|ref|WP_002793215.1| 1 Microcystin synthetase A [Microcystis aeruginosa]

Saulo Maia gi|1121307927|ref|WP_073619435.1| 1 non-ribosomal peptide synthetase [Calothrix sp. HK-06]

Saulo Maia gi|518321029|ref|WP_019491236.1| 1 non-ribosomal peptide synthetase [Calothrix sp. PCC 7103]

Saulo Maia gi|515859364|ref|WP_017289992.1| 1 hypothetical protein [Leptolyngbya boryana]

Saulo Maia gi|1084328748|ref|WP_070392807.1| 1 hypothetical protein [Moorea producens]

Saulo Maia gi|1084328752|ref|WP_070392811.1| 1 hypothetical protein [Moorea producens]

Saulo Maia gi|1084328752|ref|WP_070392811.1| 1 hypothetical protein [Moorea producens]

Saulo Maia gi|488829433|ref|WP_002741839.1| 1 McnE protein [Microcystis aeruginosa]

Saulo Maia gi|640535235|ref|WP_024968867.1| 1 non-ribosomal peptide synthetase [Microcystis aeruginosa]

Saulo Maia gi|640535236|ref|WP_024968868.1| 1 McnC protein [Microcystis aeruginosa]

Saulo Maia gi|640535236|ref|WP_024968868.1| 1 McnC protein [Microcystis aeruginosa]

Saulo Maia gi|501225153|ref|WP_012268171.1| 1 McnC protein [Microcystis aeruginosa]

Saulo Maia gi|501225153|ref|WP_012268171.1| 1 McnC protein [Microcystis aeruginosa]

Saulo Maia gi|488879470|ref|WP_002791695.1| 1 non-ribosomal peptide synthetase [Microcystis aeruginosa]

Saulo Maia gi|488879470|ref|WP_002791695.1| 1 non-ribosomal peptide synthetase [Microcystis aeruginosa]

Saulo Maia gi|488864797|ref|WP_002777036.1| 1 McnC protein [Microcystis aeruginosa]

Saulo Maia gi|488864797|ref|WP_002777036.1| 1 McnC protein [Microcystis aeruginosa]

Saulo Maia gi|488829431|ref|WP_002741837.1| 1 McnC protein [Microcystis aeruginosa]

Saulo Maia gi|488829431|ref|WP_002741837.1| 1 McnC protein [Microcystis aeruginosa]

Saulo Maia gi|504941889|ref|WP_015128991.1| 1 non-ribosomal peptide synthetase [Calothrix sp. PCC 7507]

Saulo Maia gi|501380182|ref|WP_012411748.1| 1 amino acid adenylation domain-containing protein [Nostoc punctiforme]

Saulo Maia gi|1084328881|ref|WP_070392940.1| 1 hypothetical protein [Moorea producens]

Saulo Maia gi|502446269|ref|WP_012789107.1| 1 amino acid adenylation protein [Chitinophaga pinensis]

Saulo Maia gi|1062970884|ref|WP_069351498.1| 1 hypothetical protein [Scytonema millei]

Saulo Maia gi|546233813|ref|WP_021836626.1| 1 non-ribosomal peptide synthetase [Crocosphaera watsonii]

Saulo Maia gi|757157445|ref|WP_042711533.1| 1 non-ribosomal peptide synthetase

Saulo Maia gi|757157445|ref|WP_042711533.1| 1 non-ribosomal peptide synthetase

Saulo Maia gi|757157445|ref|WP_042711533.1| 1 non-ribosomal peptide synthetase

Saulo Maia gi|757157445|ref|WP_042711533.1| 1 non-ribosomal peptide synthetase

Saulo Maia gi|657933313|ref|WP_029634518.1| 1 non-ribosomal peptide synthetase [[Scytonema hofmanni] UTEX B 1581]

Saulo Maia gi|1062969311|ref|WP_069350916.1| 1 non-ribosomal peptide synthetase [Scytonema millei]

Saulo Maia gi|504994654|ref|WP_015181756.1| 1 non-ribosomal peptide synthetase [Microcoleus sp. PCC 7113]

Saulo Maia gi|515347460|ref|WP_016862252.1| 1 non-ribosomal peptide synthetase [Fischerella muscicola]

Saulo Maia gi|918157302|ref|WP_052335078.1| 1 non-ribosomal peptide synthetase [Tolypothrix sp. PCC 7601]

Saulo Maia gi|918157302|ref|WP_052335078.1| 1 non-ribosomal peptide synthetase [Tolypothrix sp. PCC 7601]

Saulo Maia gi|493986638|ref|WP_006929441.1| 1 non-ribosomal peptide synthetase [Caldithrix abyssi]

Saulo Maia gi|493986638|ref|WP_006929441.1| 1 non-ribosomal peptide synthetase [Caldithrix abyssi]

Saulo Maia gi|923072610|ref|WP_053456024.1| 1 non-ribosomal peptide synthase [Hapalosiphon sp. MRB220]

Saulo Maia gi|923072610|ref|WP_053456024.1| 1 non-ribosomal peptide synthase [Hapalosiphon sp. MRB220]

Saulo Maia gi|493040208|ref|WP_006106018.1| 1 non-ribosomal peptide synthetase [Coleofasciculus chthonoplastes]

Saulo Maia gi|1120050422|ref|WP_072924245.1| 1 non-ribosomal peptide synthetase [Microcystis aeruginosa]

Saulo Maia gi|817700114|ref|WP_046662633.1| 1 McnC protein [Microcystis aeruginosa]

Saulo Maia gi|488864797|ref|WP_002777036.1| 1 McnC protein [Microcystis aeruginosa]

Saulo Maia gi|488879470|ref|WP_002791695.1| 1 non-ribosomal peptide synthetase [Microcystis aeruginosa]

Saulo Maia gi|501225153|ref|WP_012268171.1| 1 McnC protein [Microcystis aeruginosa]

Saulo Maia gi|1114047701|ref|WP_072065595.1| 1 hypothetical protein [Crocosphaera watsonii]

Saulo Maia gi|1044603281|ref|WP_065418850.1| 1 hypothetical protein [Clostridium beijerinckii]

Saulo Maia gi|494515511|ref|WP_007304969.1| 1 non-ribosomal peptide synthetase [Crocosphaera watsonii]

Saulo Maia gi|852246069|ref|WP_048319466.1| 1 hypothetical protein

Saulo Maia gi|515861070|ref|WP_017291698.1| 1 non-ribosomal peptide synthetase [Leptolyngbya boryana]

**Supporting Table 5.** Concentrations of microcystins, nodularins and cylindrospermopsin obtained by ELISA test. Mean (± Standard Deviation).

| **Sample** | **Microcystins/Nodularins (ng.ml^-1^)** | **Cylindrospermopsin (ng.ml^-1^)** |
| --- | --- | --- |
| Araçagi 1 | 0.5 ± 0.2 | 0.08 |
| Araçagi 2 | 0.3 ± 0.07 | 0.05 |
| Saulo Maia 1 | 0.15 | <0.05 |
| Saulo Maia 2 | <0.15 | <0.05 |
| Mazagão | <0.15 | <0.05 |

**Supporting Figure 1.** Cell abundance counts obtained by flow cytometry. Comparison of abundance counts for total autotrophs (A) and cyanobacteria (B) in each pond. The values represent averages and standard deviation (SD). *P*-values of one-way analysis of ANOVA with Bonferroni–Holm test correction are denoted with asterisks (* ≤ 0.05). All sampling ponds were statistically significant compared to the control. The counts are displayed on logarithmically scaled axes.


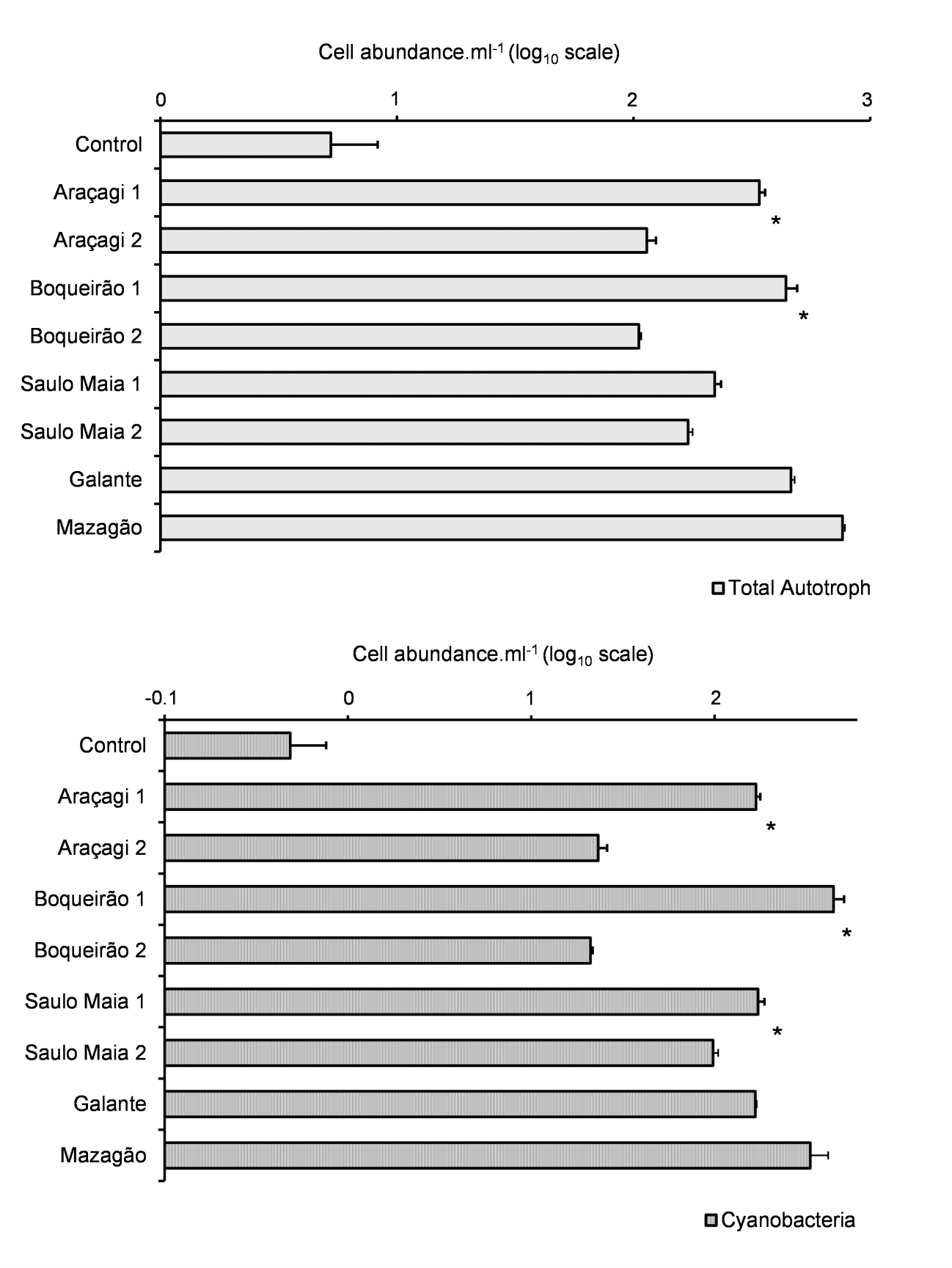


**Supporting Figure 2.** Major taxonomic contributors of the inland waters. A) The major phyla found in the metagenomes of Araçagi, Boqueirão, and Saulo Maia major ponds. B) The major phyla found in the metagenomes of Galante and Mazagão minor ponds. Control, bottled mineral water.


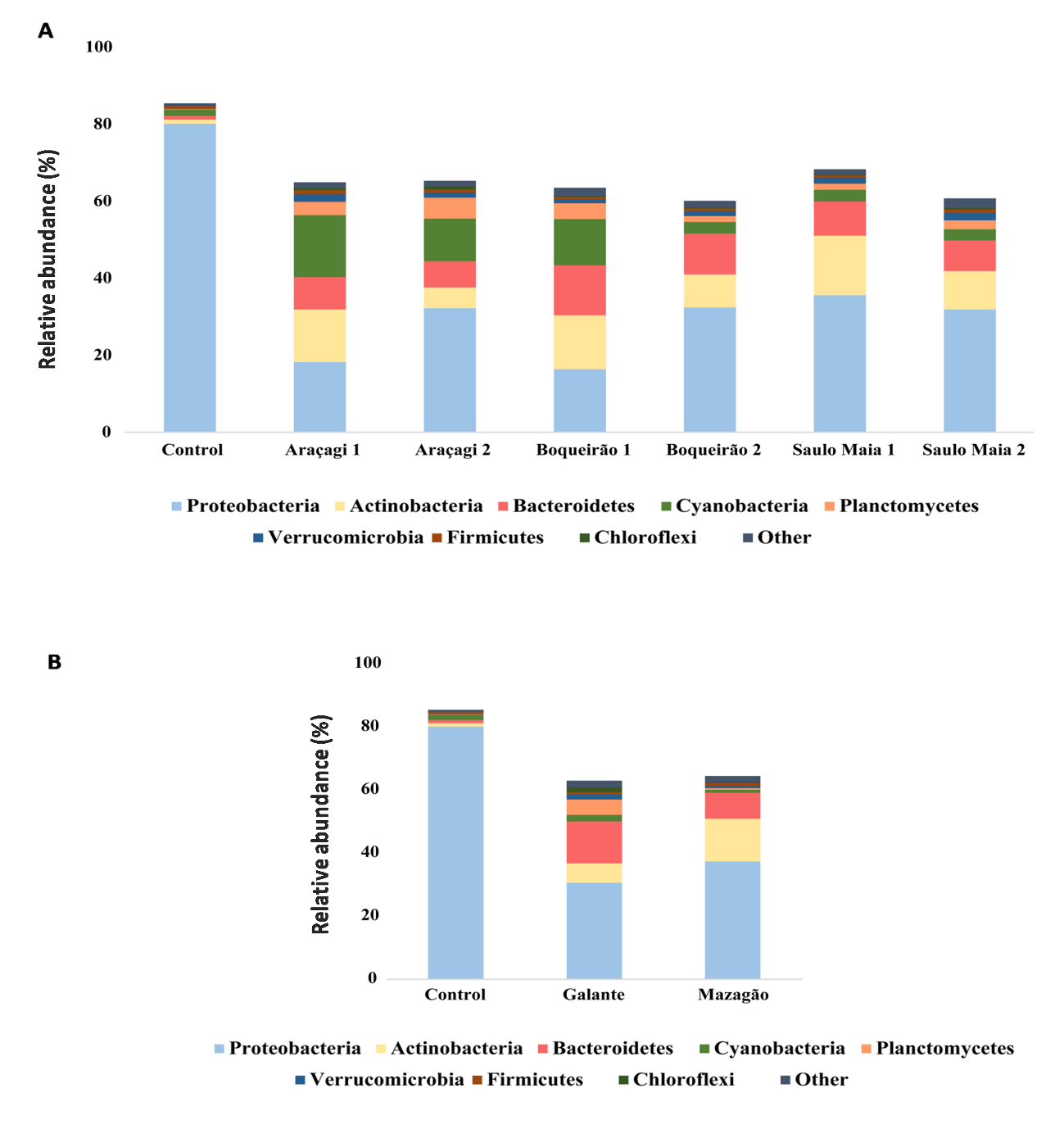


**Supporting Figure 3.** Subsystems analyses in the ponds. Relative abundance of metabolic potential based on SEED subsystems, level 1 (A), and level 2 (B) in all metagenomes. The assigned function for the metagenomes sequences showed a homogeneous profile. Most of the metagenomic sequences (52.7% from the total of annotated sequences) were assigned to one of the following categories: (i) cofactors, vitamins, prosthetic groups, pigments (9.15%; N = 38,669); (ii) carbohydrates (8.6%; N = 36,418); (iii) amino acids and derivatives (8.2%; N = 34,835); (iv) protein metabolism (8%; N = 34,150); (v) virulence, disease and defense (5.4%; N = 22,864); (vi) DNA metabolism (5.2%; N = 22,328); (vii) RNA metabolism (4.4%; N = 18,679); and (viii) respiration (3.5%; N = 14,794). DNA replication and repair, mismatch repair functions appears contributing with an average of 1.8% ± 0.03, and 1.36% ±1.11 of abundance considering the total of SEED level 2 and level 3, respectively. Boqueirão is also named Epitácio Pessoa pond.


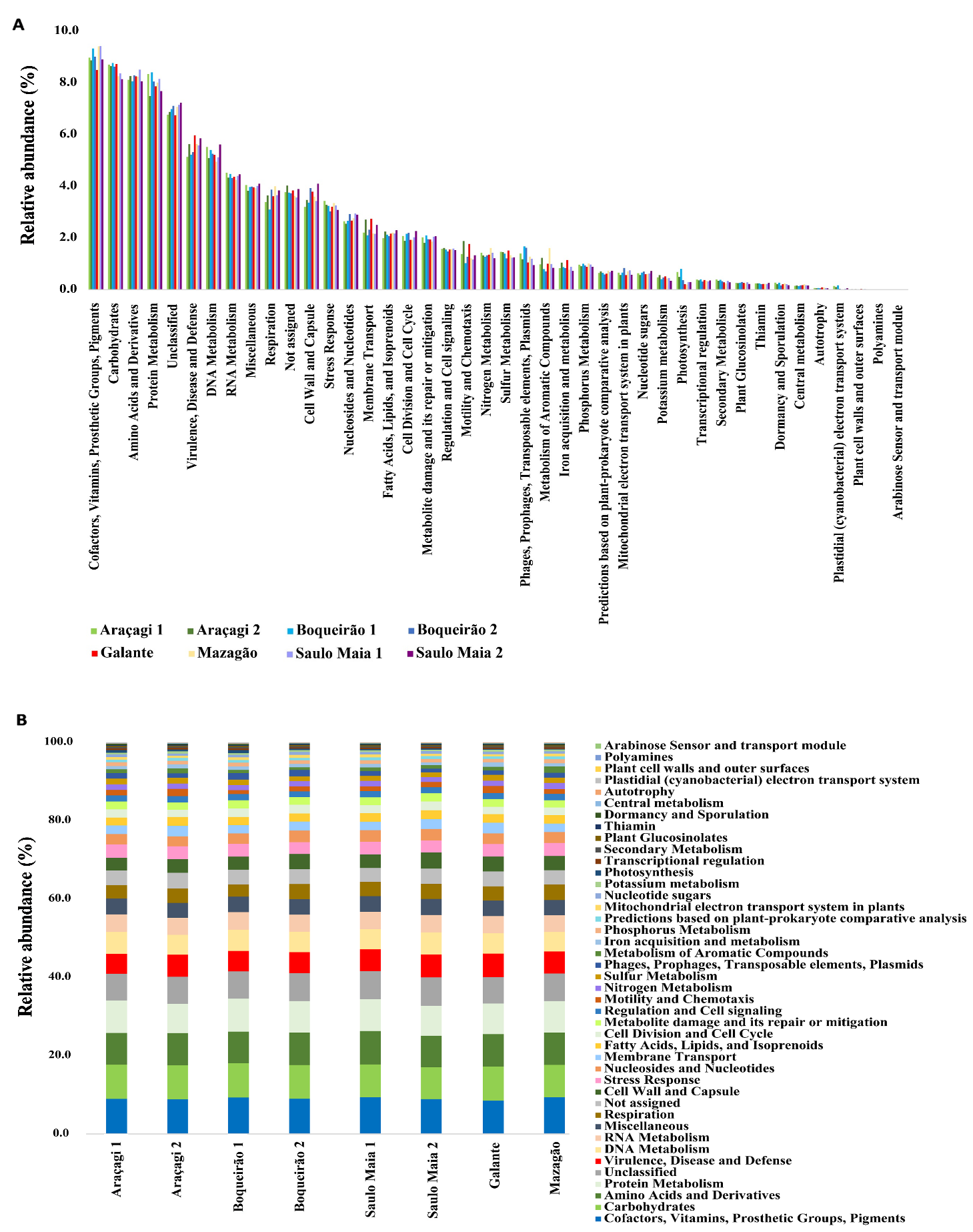

Supplement: Supplementary file 1 [file Data_Sheet_1.docx]
